# The Effect of Exercise Training on Blood Lipids: A Systematic Review and Meta-analysis

**DOI:** 10.1007/s40279-024-02115-z

**Published:** 2024-09-27

**Authors:** Neil A. Smart, David Downes, Tom van der Touw, Swastika Hada, Gudrun Dieberg, Melissa J. Pearson, Mitchell Wolden, Nicola King, Stephen P. J. Goodman

**Affiliations:** 1 Department of Exercise Physiology, School of Science and Technology, University of New England, Armidale, NSW 2351 Australia; 2 Department of Pharmacy, Kathmandu University, JG9Q+PGG, Dhulikhel, 45200 Nepal; 3 Department of Physical Therapy, Jamestown University, Fargo, ND USA; 4 School of Biomedical Sciences, University of Plymouth, Devon, UK

## Abstract

**Background:**

Dyslipidemia is a primary risk factor for cardiovascular disease (CVD). Exercise training (EXTr) improves some lipid markers but not others; the literature is dated and analyses may be underpowered.

**Objectives:**

To clarify which lipid markers are altered with ExTr and establish if information size had yet reached futility.

**Methods:**

We conducted a systematic review/meta-analysis, with meta-regression, to establish expected effect size in lipid profile with aerobic (AT), resistance (RT) and combined (CT = AT + RT) ExTr. We conducted trial sequence analysis (TSA) to control for type I and II error and establish if information size had reached futility.

**Results:**

We included 148 relevant randomized controlled trials (RCTs) of ExTr, with 227 intervention groups, total 8673 participants; exercise 5273, sedentary control 3400. Total cholesterol (TC) MD – 5.90 mg/dL (95% confidence interval (CI) – 8.14, – 3.65), high-density lipoprotein cholesterol (HDL) 2.11 (95% CI 1.43, 2.79), low-density lipoprotein cholesterol (LDL) – 7.22 (95% CI – 9.08, – 5.35), triglycerides – 8.01 (95% CI – 10.45, – 5.58) and very low-density lipoprotein cholesterol (VLDL) – 3.85 (95% CI – 5.49, – 2.22) all showed significant but modest 3.5–11.7%, improvements following ExTr. TSA indicated all analyses exceeded minimum information size to reach futility. CT was optimal for dyslipidemia management. Meta-regression showed every extra weekly aerobic session reduced TC – 7.68 mg/dL and for every extra week of training by – 0.5 mg/dL. Each minute of session time produced an additional 2.11 mg/dL HDL increase.

**Conclusion:**

TSA analysis revealed sufficient data exist to confirm ExTr will improve all five lipid outcomes. CT is optimal for lipid management. The modest effect observed may moderate dyslipidemia medication for primary prevention. Prediction intervals suggest TC, HDL, LDL and TGD are only improved in one-quarter of studies.

**Supplementary Information:**

The online version contains supplementary material available at 10.1007/s40279-024-02115-z.

## Key Points

**Table:** 

This work has clarified which lipid outcomes respond to exercise training and to the extent that the modest effects observed may moderate dyslipidemia medication for primary disease prevention.
This work demonstrates combined aerobic and resistance exercise is optimal for lipid management, but variations to exercise prescriptions are required to manage different types of dyslipidemia.
The Trial Sequence Analysis revealed this to be the first work with sufficient data to declare statistical futility for all five lipid outcomes.

## Introduction

Dyslipidemia (DS) presents in different forms (see Table 1) and is a primary risk factor for cardiovascular disease (CVD) [1]. Historically DS management guidelines cited thresholds for lipid sub-fractions [2]. Current guidelines offer flexible treatment thresholds for co-morbid conditions such as diabetes mellitus and the treatment goal, for example primary prevention [3, 4].

**Table 1:** Different types of dyslipidemia

Type	HDL	LDL	Triglycerides
Hyperlipidemia	Low	High	
Hypoalphalipoproteinemia	Low		
Mixed hyperlipidemia	Low	High	High
Hypertriglyceridemia	Low	High	High

*HDL* high-density lipoprotein cholesterol, *LDL* low-density lipoprotein cholesterol

While medication remains the cornerstone to DS management, in sub-clinical populations lifestyle change may be initially required [5]. The two lifestyle therapies are dietary management and physical activity (PA). Dietary management involves limiting saturated fat intake, which reduces hepatic lipid production, achieves energy balance, and reduces costs and side effects associated with medication. This work focuses on exercise training (ExTr) effects for DS.

A 2001 review suggested exercise can modestly increase high-density lipoprotein cholesterol (HDL) and reduce triglycerides (TGD) with total cholesterol (TC) and low-density lipoprotein cholesterol (LDL) changes seldom observed [6]. Kraus et al. [5] suggested exercise-induced favourable changes in LDL and very low-density lipoprotein cholesterol (VLDL) particle size. Other work [7, 8] suggested 900 kcal, or 120 min, of weekly energy expenditure may modestly raise HDL and lower TGD. Kodama et al. [9] confirmed favourable HDL changes from exercise training were modest and more pronounced with a body mass index below 28 kg.m^−2^ and baseline TC below 220 mg/dL. Kelley and Kelly’s 2009 work urged caution in using resistance training (RT) to manage DS, due to efficacy concerns identified by prediction intervals [10]. Other work noted the paucity of good quality trials [11]. Mann et al. [12] identified exercise mode, intensity and total energy expenditure influences lipid profile, noting the limited comparisons of AT, RT and combined (AT + RT) modalities. Despite some studies showing beneficial effects of exercise on LDL [8], results are conflicting. Wang et al. [13] suggested LDL reductions are more likely when accompanied by weight loss.

Previous works were not systematic [14] or included non-randomized trials [13, 15]; limited pooled data analyses have been previously published [16, 17]. It remains unclear if the minimum information size exists. Igarishi et al. [18] conducted a pooled analysis, reporting AT exhibits the largest participant numbers to date, but even this work has insufficient data to meet statistical futility.

In summary, the literature does not include a contemporary comprehensive synthesis that has reached statistical futility, clarifies which lipid outcomes respond to exercise, the effect size, and if this varies with different types of exercise.

We conducted a systematic review, meta-analysis and subsequent meta-regression of published randomized, controlled trials on the effects of ExTr on DS. The aims of this work were to (i) identify the expected size of change in primary DS markers following different types of ExTr; (ii) clarify if ExTr reduces LDL, TGD and VLDL; (iii) conduct a trial sequential analysis to control the risks of Type I and II errors, and to estimate if the required information size had been reached.

## Methods

### Protocol

This meta-analysis protocol was registered with the Open Science Framework at https://osf.io/d8uty/. As this was a secondary analysis of publicly available de-identified data, ethics approval was not sought.

### Search Strategy

We conducted a systematic literature search in PubMed, Web of Science and the Cochrane Library of Controlled Trials up until 30 September, 2023. The search strategy included key words related to DS, ExTr and related MeSH terms (see Online Supplementary Material (OSM) Table S1). We also manually searched reference lists from systematic reviews and eligible studies for additional works.

### Study Eligibility

#### Included Studies

**Fig. 1:**
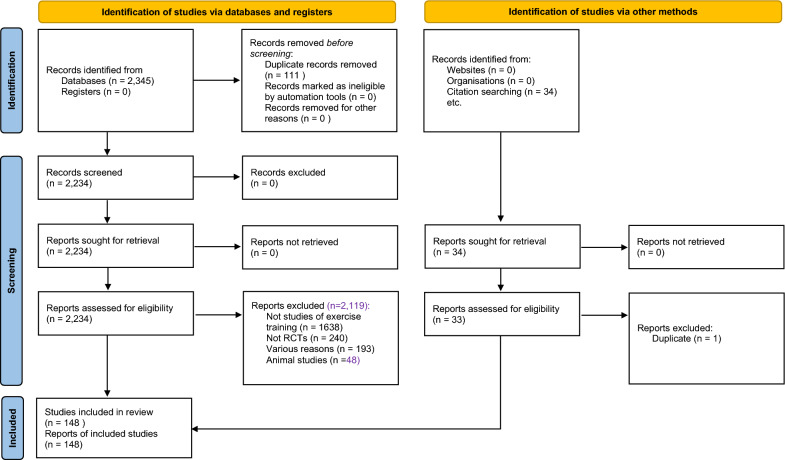
PRISMA (Preferred Reporting Items for Systematic Reviews and Meta-Analyses) flow diagram of study selection process. *RCT* randomized controlled trial

Two authors (TVDT, DD) assessed articles independently for eligibility and consulted a third reviewer (NAS) for resolution of disagreements (see PRISMA (Preferred Reporting Items for Systematic Reviews and Meta-Analyses) diagram Fig. 1). We included randomized controlled trials (RCTs) conducted on adult humans that reported change in TC, HDL, LDL, TGD or VLDL before and after exercise training. Studies of AT, RT or combined training (CT) were considered provided the intervention was for a minimum of 3 weeks’ duration. Crossover studies were only excluded if the washout period was less than 2 weeks. There was no language restriction.

#### Excluded Studies

Studies with participants with known cardiovascular disease, cancer, spinal cord injury or HIV and pregnant women were excluded. We deemed these medical conditions would affect DS or the ability to participate unencumbered in ExTr.

#### Comparisons

Included studies compared ExTr intervention group(s) to a non-exposed, matched health status, control (usual care) group or a sham intervention group.

#### Outcome Measures

The primary outcome measures were change in blood levels of TC, HDL, LDL, TGD and VLDL.

### Data Extraction

From each study we extracted the authors' names and year of publication. In addition, the characteristics of training interventions (i.e., exercise program delivery venue and method, type of exercise, intensity, duration and frequency of the protocol) and medication use. The mean change and standard deviation of the desired outcome variables were recorded. Data were entered into Excel data extraction sheets.

### Statistical Analyses

Meta-analyses were completed in STATA V.18 [19]. We converted all values to mg/dL using appropriate conversion factors [20]. Individual meta-analyses were completed for continuous data by using the mean baseline follow-up change and standard deviation (SD). Where change in SD was not provided we imputed this using established methods [21].

A Der Simonian-Laird, inverse variance, random effects model was employed [22]. Mean differences were used for outcome measures. We supplemented 95% confidence intervals (95% CIs) with 95% prediction intervals (95% PIs) so we could assess the distribution of effect sizes across all studies [23]. In studies with multiple exercise intervention groups, data were separated for each intervention group and the control group participant data were divided evenly providing an intervention comparator.

#### Pooled Data Analyses

Forest plots were not generated as many included studies rendered the font illegible, so summary statistics were instead presented. Both confidence and prediction intervals (95%) were calculated using an on-line calculator [24].

#### Trial Sequence Analysis (TSA)

To control the risks of Type I and II errors, random errors associated with repetitive testing when updating reviews, and to estimate required information size we used methods and TSA software of the Clinical Trials Unit (CTU), Copenhagen, DK [25]. As directed by the CTU Copenhagen, we compared the Der Simonian-Laird and Sidik-Jonkman models and found little difference (OSM Table S2), so we defaulted to the former. By performing both O’Brien-Fleming and Law of Iterated Logarithm (LIL) TSA, we were able to calculate the necessary number of participants and information size (IS) needed in a meta-analysis to detect or reject anticipated intervention effects, thereby controlling Type I and Type II error risk. We investigated possible heterogeneity by calculating inconsistency (*I*^2^).

#### Sensitivity Analyses

We conducted sensitivity analyses for studies scoring 10 or more on the Tool for the assEssment of Study qualiTy and reporting in EXercise (TESTEX) scale [26]. TSA default analysis ignored studies making < 1% addition to cumulative data.

#### Meta-Regression

We performed meta-regression for the significant meta-analyses of TC, HDL, LDL, TGD and VLDL if they had more than ten included studies.

#### Heterogeneity, Risk of Bias, and Publication Bias

Statistical heterogeneity (*I*^2^) was assessed for inconsistency among studies with the values of 25%, 50% and 75%, representing low, moderate and high degrees of heterogeneity, respectively [27], and the 95% PIs. The revised risk of bias (ROB) 2.0 tool for independent and crossover studies was used to assess ROB [28]. Small study effects were established by comparing studies with 20 or less participants with those with > 20 participants. Publication bias, and also possible small study effects, were evaluated by visual inspection of the funnel plot for all outcomes with Egger's regression test, with 10% statistical significance [29].

### Study Quality Assessment

The validated Tool for the assEssment of Study qualiTy and reporting in EXercise (TESTEX) [26] was used to assess the methodological quality of the included studies. This 15-point assessment criteria (5 points for study quality and 10 points for reporting) is designed specifically for use in exercise-training studies. We considered studies with TESTEX scores of 10 or above to be of good quality [16], and conducted sub-analyses accordingly.

## Results

### Search Results

Our initial search yielded 2345 hits. After removing 111 duplicates, 2234 remained. Of these, 1638 were not exercise training trials, 240 were not RCTs, and 48 were animal studies, all of which were removed, leaving 308. We excluded 193 RCTs for various reasons (see OSM Table S3), leaving 115 studies. We found an additional 34 from reference lists of included studies, one of which was excluded as a duplicate, leaving 148 included studies with 227 intervention groups, with a total of 8673 participants: 5273 exercise participants and 3400 sedentary control participants.

### Characteristics of Included Studies

AT was employed by 164 intervention groups, RT by 32 and CT by 31. Median training frequency was three weekly sessions, used in 151 intervention groups. Vigorous-intensity training was most frequently used (103), with moderate (96), high (27) and low intensity used in just three intervention groups. Program duration ranged from 3 to 52 weeks, with 12 weeks being the most common. The mean number of exercise and control participants was 23 and 15, respectively, total 38 per study. OSM Table S4 further summarizes study characteristics.

Of the 148 included studies, 91 did not provide any medication usage information; 41 studies reported participants did not use lipid-lowering medication; 14 studies reported partial use of lipid lowering medication; and 2 studies reported 100% of participants were using lipid lowering medication. The incomplete and skewed nature of these data, which were also provided at a group, not individual level, meant we were not justified in drawing conclusions about the concurrent effects of exercise training and use of lipid lowering medication.

### Overall Pooled Analysis of 95% Confidence Intervals and Prediction Intervals

**Fig. 2:**
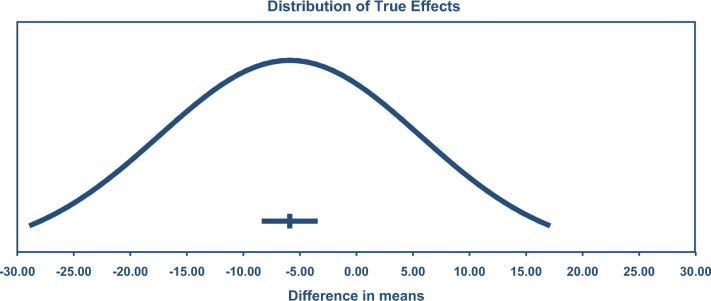
Change in total cholesterol 95% confidence (CIs) versus prediction intervals (PIs). The mean effect size is – 5.90 mg/dL with a 95% CI of – 8.15 to – 3.65 mg/dL. The true effect size in 95% of all comparable populations falls in the interval – 29.72 to 19.92 mg/dL

A combined analysis of all 211 exercise-training studies reported TC (4542 exercise/3073 controls) was lower by – 5.90 mg/dL or 0.15 mmol/L (95% CI – 8.14, – 3.65), see Fig. 2. TSA calculated a minimum information size (IS) of 3777 participants was required to support the findings.

**Fig. 3:**
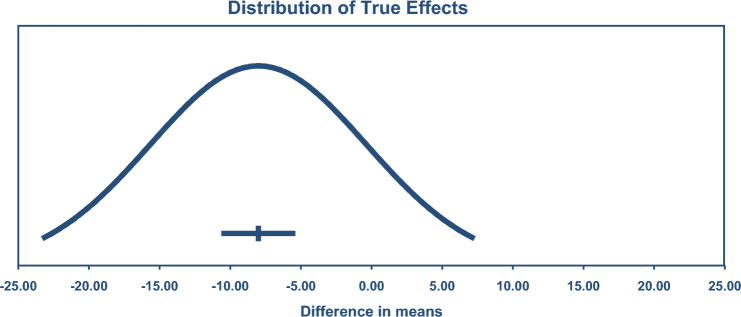
Change in low-density lipoprotein cholesterol (LDL) 95% confidence (CIs) versus prediction intervals (PIs). The mean effect size is – 7.22 mg/dL with a 95% CI of – 9.09 to – 5.35 mg/dL. The true effect size in 95% of all comparable populations falls in the interval – 23.54 to 9.10 mg/dL

LDL was reduced by – 7.22 mg/dL or 0.19 mmol/L (95% CI – 9.08, – 5.35), see Fig. 3 (*n* = 178; exercise 4143/2724 control). TSA calculated a minimum IS of 1,558 participants was required to support the findings.

**Fig. 4:**
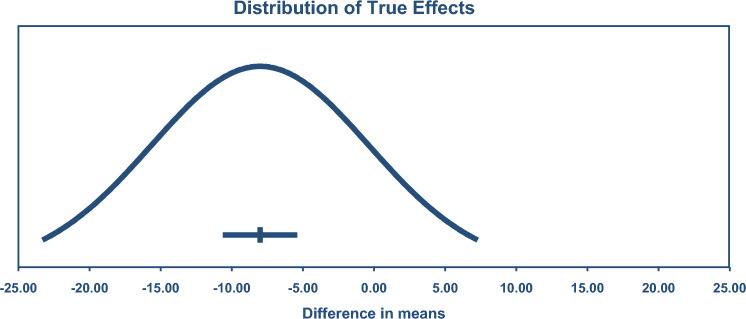
Change in triglycerides 95% confidence (CIs) versus prediction intervals (PIs). The mean effect size is – 8.01 mg/dL with a 95% CI of – 10.44 to – 5.58 mg/dL. The true effect size in 95% of all comparable populations falls in the interval – 23.13 to 7.11 mg/dL

TGD was reduced by – 8.01 mg/dL or 0.09 mmol/L (95% CI – 10.45, – 5.58), see Fig. 4 (*n* = 200; exercise 4,26/control 3,81). The TSA calculated that a minimum IS of 7,69 participants was required to support the findings. This number was exceeded by only 38, meaning the requirement was only just met.

**Fig. 5:**
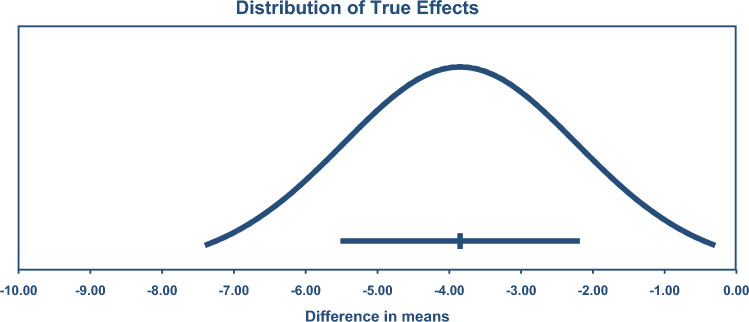
Change in very low-density lipoprotein cholesterol (VLDL) 95% confidence (CIs) versus prediction intervals (PIs). The mean effect size is – 3.85 mg/dL with a 95% CI of – 5.48 to – 2.22 mg/dL. The true effect size in 95% of all comparable populations falls in the interval – 7.37 to – 0.33 mg/dL

VLDL was reduced by – 3.85 mg/dL or 0.10 mmol/L (95% CI – 5.49, – 2.22), see Fig. 5 (*n* = 23; 413 exercise/317 control). TSA calculated a minimum IS of 554 participants was required to support the findings.

**Fig. 6:**
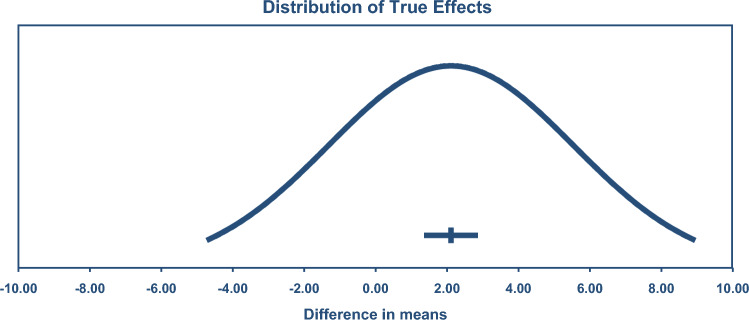
Change in high-density lipoprotein cholesterol (HDL) 95% confidence (CIs) versus prediction intervals (PIs). The mean effect size is 2.11 mg/dL with a 95% CI of 1.43–2.79 mg/dL. The true effect size in 95% of all comparable populations falls in the interval – 4.66 to 8.88 mg/dL

HDL was significantly higher by 2.11 mg/dL or 0.05 mmol/L (95% CI 1.43, 2.79), see Fig. 6 (*n* = 216, 5,018 exercise/3,310 control). TSA calculated a minimum IS of 2,724 participants was required to support the findings.

### Law of Integrated Logarithm (LIL) Analyses

With the exception of VLDL, which exhibited the smallest information size, the LIL supported favourable change findings for TC, HDL, LDL and TGD, using the O’Brien-Fleming technique set at 5% significance.

The weighted percentage change in lipids following exercise training ranged from 3.48% for HDL to 11.68% for VLDL. OSM Figs. S1–S5 show funnel plots for the five lipid outcome measures, containing effect sizes, 95% CIs and 95% PIs. OSM Figs. S6a–S6c show default settings for the TSA analyses. OSM Figs. S7–S16 show the O’Brien-Fleming adjustment boundary and the Law of Iterated Logarithm (LIL) penalized analyses for the TSA analysis of each of the five lipid outcomes.

Prediction intervals suggested only VLDL remained significant. Prediction intervals were (mg/dL): TC 95% PI – 28.72 to 16.92, so 37.1% of the studies showed no beneficial effect of exercise training; HDL 95% PI – 4.66 to 8.88, 34.2% of the studies showed no beneficial effect: LDL 95% PI – 23.54 to 9.10, 27.9% of the studies showed no beneficial effect; TGD 95% PI – 23.13 to 7.11, 23.5% of the studies showed no beneficial effect; VLDL 95% PI – 7.37 to – 0.33, 100% of the studies showed a beneficial effect.

### Exercise Type

**Fig. 7:**
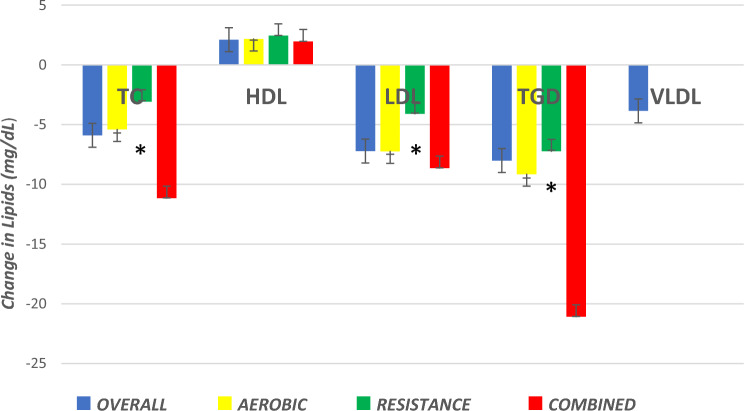
Summary of change in outcome measures with different types of exercise training. *Not significant,* p* > 0.05 for all resistance training except high-density lipoprotein cholesterol (HDL) analysis. As only 23 very low-density lipoprotein cholesterol (VLDL) studies were included, we did not conduct sub-analyses. *TC* total cholesterol, *LDL* low-density lipoprotein cholesterol, *TGD* triglycerides

When separate analyses by exercise type were performed, AT significantly reduced TC, LDL, TGD, and VLDL and raised HDL. CT produced significant reductions in TC, LDL, TGD and VLDL and an increase was observed in HDL. RT only improved HDL (see Fig. 7).

### Study Quality Effects and Sub-analyses

The median study quality TESTEX score was 7. Several study quality items were, in general, rarely conducted; these included: specifying randomization method used (31% of studies); allocation concealment (15%); blinding of assessors (14%); reporting adherence, adverse events and participant withdrawal rates > 15% (all < 30%); performing intention-to-treat analyses (27%); physical activity monitoring in control group (7%). A summary is provided in OSM Table S5.

### Aerobic Sub-analyses

TC and TGD were all favourably changed in studies with TESTEX scores < 10, but not ≥ 10. HDL, LDL and VLDL were significantly improved irrespective of TESTEX score.

### Combined Sub-analyses

TC, HDL and TGD were significantly improved in studies with TESTEX scores < 10, but not ≥ 10. LDL and VLDL were significantly improved irrespective of TESTEX score.

### Resistance Sub-analyses

HDL was only significantly increased with studies with TESTEX < 10.

### Leave-One-Out Meta-analysis

We conducted leave-one-out meta-analysis for TC, HDL, LDL and TGD; due to the larger number of included studies no single study had an effect on statistical significance.

#### Heterogeneity

Heterogeneity was moderate to high for the majority of analyses. Likely causes of heterogeneity were variations in study size, type, modality, session, frequency, intensity and program duration of exercise. These variables were examined using meta-regression to explain heterogeneity.

#### Meta-Regression

Meta-regression models were developed for aerobic exercise for TC, HDL and LDL. The model for TC suggested that for every extra weekly session, a reduction of – 7.68 mg/dL or – 0.20 mmol/L occurred and for every extra week of training the TC reduction was – 0.5 mg/dL or – 0.013 mmol/L. The TC model also showed that for each additional study participant an increase of 0.30 mg/dL or 0.078 mmol/L was observed, indicating a small study effect.

Each minute of session time produced a 2.11 mg/dL or 0.055 mmol/L HDL increase. Each yearly increase in participant age produced a 0.25 mg/dL or 0.27 mmol/L LDL increase, while each additional study participant raised LDL by 0.83 mg/dL or 0.022 mmol/L.

#### Publication Bias

Funnel plots of the five primary outcomes can be seen in OSM Figs. S1-S5. Visual inspection showed some evidence of publication bias for TC (p = 0.77) and LDL (p = 0.55), but the Egger tests were not significant.

#### Risk of Bias

Risk of bias was completed and summary figures for each of the outcomes are published online due to formatting output size (https://osf.io/dbeh9). For TC, 212 independent intervention groups were appraised. Low risk of bias was found in three, 92 showed some concerns, and the remaining 117 were found to have high risk of bias. Of the 216 intervention groups for HDL, five studies showed low risk of bias, 92 showed some concerns, and 122 contained high risk of bias. Among the 178 intervention groups for LDL, five observations showed low risk of bias, 79 some concerns, and 94 high risk of bias. For VLDL, 23 observations showed some concerns and 11 showed high risk of bias. For TGD, 200 intervention groups were examined, and five of these showed low risk of bias, 88 indicated some concerns, and the remaining 107 studies displayed high risk of bias.

#### Certainty of Evidence

The number of studies and participants in the TC, HDL, LDL and TGD analyses was large enough to exceed the futility threshold in all cases. This adds to the certainty these lipids are improved with exercise. In contrast, the prediction intervals suggest that, aside from VLDL where 100% of studies showed a beneficial effect, ExTr does not appear to exert benefit in 37.1% of TC, 34.2% of HDL, 27.9% of LDL, and 23.5% of TGD studies. Furthermore, a high degree of heterogeneity, some evidence of publication bias, and a hint of small study effects mitigates this certainty.

## Discussion

This is the most comprehensive pooled analysis on ExTr for DS management to date and the first to demonstrate statistical futility for all five outcome measures, utilizing TSA analyses. Our work produced clear recommendations on the optimal exercise prescription for DS management. Data from 148 RCTs with sufficient information size demonstrated favourable, modest benefits ranging from 3.5% to 11.7%.

Changes of 3.5–11.7% following ExTr will offer primary cardiovascular disease (CVD) prevention that may delay DS medication commencement, or reduce dosage. The quality and reporting of published studies on exercise training and lipid management was generally poor, with most studies yielding a TESTEX score < 10. There was minimal evidence of publication bias, only for TC and LDL.

### Total Cholesterol (TC)

AT and CT produced significant TC reductions similar to those previously reported [14]. CT produced the greatest TC reduction, but this was not statistically different from AT; RT showed no TC benefit. CT may be most beneficial because of different mechanisms by which AT and RT exert benefit. In general, if duration is standardized, AT consumes relatively more calories than RT [30], even when considering the post-exercise effects of these two exercise modes [31]. Intuitively AT will generally offer greater fuel utilization than RT. RT will generate small increases in muscle mass that will raise basal metabolism, providing additional calorie burning. during the activity and in the post-exercise phase [31]. A similar, cumulative, beneficial effect from CT has been observed for HbA1C% (glycated haemoglobin) control in people with diabetes [32]. Previous work has shown CT [15] with the aerobic component conducted at vigorous [9] or high intensity [5] to be optimal.

Our analysis found superior TC changes with a combination of aerobic and resistance modalities, which is consistent with previous work [15], although a range of study quality designs were considered in that analysis.

Meta-regression showed every extra weekly session and every extra week of training further reduced TC, while each additional study participant raised TC. These finding suggest that more than three sessions weekly for > 12 weeks and small group exercises classes may be optimal for reducing TC.

### High-Density Lipoprotein Cholesterol (HDL)

Following all exercise training modalities, HDL was increased. Durstine et al. [14] suggested Level 1A/B evidence existed that exercise program volume rather than the frequency or intensity was of primary importance for lowering TC and HDL. Kodama et al. [9] suggested that a weekly expenditure of 900 kcal or 120 min, presumably at moderate intensity, is the energy volume threshold for physical activity or exercise required to elicit favourable changes in HDL. Our findings and those of some others contradict this [9, 13]. Wang et al. [13] found an improved lipid profile is more likely when accompanied by weight loss. The shift of focus away from exercise volume is perhaps partially explained by the increasing number of published studies that have focused on training at vigorous or high intensities [33], acknowledging intensity is a function of exercise volume. There is currently a transition in exercise guideline papers towards increasing the ‘moderate intensity ceiling’ that was historically the cornerstone of exercise programming [34]. Both continuous and intermittent but not mixed aerobic ExTr delivery increased HDL. Session time showed > 30 min produced a statistically significant HDL increase where shorter sessions did not. Meta-regression showed that each additional minute of session time may further improve HDL. With respect to RT, the mechanism by which HDL is raised is not well understood, but may be a similar or shared mechanism with AT [35]. Recent work has shown that skeletal muscle mass is positively correlated with serum HDL levels [35]. Vatani et al. [36] found that RT at higher (80–90% 1 RM (repetition maximum)), but not lower intensity, produced significant HDL increases, but other work has contradicted this [36]. Our own hypothesis is that change in HDL is driven by energy expenditure and the metabolic disruption resulting from RT, and especially that RT exceeding certain intensity or volume thresholds may lead to favourable HDL change.

### Low-Density Lipoprotein Cholesterol (LDL)

Combined and aerobic training produced LDL reductions, while RT did not. Meta-regression showed each yearly increase in participant age and each additional study participant raised LDL. These findings suggest smaller studies produce greater benefit. Small study effects in meta-analysis are well documented [36].

### Triglycerides (TGD)

AT and CT reduced TGD but RT did not.

### Very Low-Density Lipoprotein Cholesterol (VLDL)

AT and CT, but not RT, reduced VLDL.

### Mechanism by Which Different Types of Exercise Effect Serum Lipid Change

Exercise energy expenditure is considered as the primary determinant of change in serum lipid concentration [6]. Recently there have been a number of published trials that have examined the effect of exercise training, above moderate intensity, on cardiovascular risk factors, including lipid profile. Exercise intensity, particularly during aerobic training, is of course one function of exercise energy expenditure, so it would be intuitive to suggest that both intensity and volume could determine change in serum lipids.

Previous summary work on liver enzymes and inter-hepatic fat, in people with non-alcoholic fatty liver disease, has shown that an aggregate exercise program energy expenditure of around 10,000 kcal is required to elicit improvement [37], and this may take several weeks to achieve. The liver is the major organ involved in lipid metabolism, so parallels with this current analysis may be drawn. More often than not AT is likely to utilize more energy expenditure per unit time than RT, due to the latter possessing an inherently intermittent nature. Further, recent work on vigorous to high intensity exercise may have opened up the possibility that energy expenditure thresholds, to elicit lipid profile changes, may also be accomplished with shorter duration, more intense (than moderate) sessions. We hypothesise that exercising at vigorous or high intensity may require post-exercise adjustments in fuel conversion (particularly gluconeogenesis from fat), and this may have a greater impact on stored adipose tissue than exercise at moderate intensity. In turn, this may have a more profound effect on the lipid profile.

Work has shown that, in people who are overweight, attenuation of exercise training-induced changes in lipids may occur [9], while exercise with diet-induced weight loss has been shown to improve the lipid profile [38]. Beneficial effects of RT in older men but not older women have been reported [39]. Together this information suggests a potential link between exercise, weight loss and lipid profile that may explain why RT only had a borderline beneficial effect on HDL, and no effect on other lipids in our analysis.

The specific physiological mechanisms of change may involve increased activity of lipoprotein lipase (LPL) responsible for hydrolysis of chylomicrons, VLDL and TG [13]. PCSK9 is a novel biomarker of LDL clearance and a target of lipid therapy. Previous work reported significant PCSK9 reductions accompanied by lower LDL levels after 3 months of regular exercise training [40].

### Cardiovascular Risk Reduction from Exercise and Lipid-Lowering Medication

European [41] and USA [3] treatment targets suggest LDL should be maintained around or below 115 mg/dL or 3.0 mmol/L [13] and in those with raised LDL a 50% reduction should be achieved, where possible, with lipid-lowering medication. In our analysis, we showed that exercise training reduced LDL by – 7.22 mg/dL or 0.19 mmol/L. The LDL reduction from exercise training was 6.33%, if one assumes a baseline of 115 mg/dL or 3.0 mmol/L, which is much below the 50% target assigned to lipid-lowering medication. Every 38.5 mg/dL or 1 mmol/L reduction in LDL is associated with a reduction in the risk of cardiovascular events by 21–25% [42]. One could therefore expect exercise training to typically reduce the risk of a cardiovascular atherosclerotic event by 4–5%. The question of whether the lipid-lowering effects of medication and exercise training are additive or not cannot be answered as study designs have yet to address this. Despite the poor understanding of the interaction between lipid-lowering medication and exercise, it is reasonable to assume that exercise training should remain a first-line treatment offering up to 5% risk reduction in cardiac events, but potentially, when used as an adjunct to lipid-lowering drugs, a potential risk reduction of up to 30% could be possible. Evidence suggests that atherosclerotic plaques are unlikely to further develop if LDL is at or below 70 mg/dL or 1.8 mmol/L [43]. Using this evidence, the minimal clinically important difference for LDL lowering for someone with a baseline LDL of 115 mg/dL or 3.0 mmol/L is 46 mg/dL or 1.2 mmol/L (3.0–1.2 = 1.8 mmol/L), which was not achieved in this analysis with exercise training alone.

### Strengths and Limitations of This Analysis

The initial protocol did not include TSA analyses, but we felt these were warranted to highlight shortcomings of previous summaries. 116 of the 148 (78%) included studies exhibited a TESTEX score < 10. There was a trend towards larger favourable lipid profile changes in these poor-quality studies for TC and TGD. This suggests, however, that a lack of key study design measures such as investigator blinding and failure to capture control participants’ crossover to exercise may lead to results bias. It is plausible though that smaller studies are easier to manage and more individualized attention is given to participants.

There was variation in the exercise training parameters: exercise type, frequency, intensity, session duration and program duration. We noted that session time and exercise intensity were often increased as studies progressed, presumably because people improved their exercise tolerance. We have taken the information as it was reported in the included studies but we acknowledge there could have been deviations from the stipulated protocols in some studies.

In a similar manner, ROB was relatively high for a large portion of the studies we examined. Future interventions should consider implementing approaches that minimize this bias to ensure higher quality evidence can arise. Heterogeneity was very high (i.e. > 75%) for the majority of the analyses, and this is consistent with the study quality assessment (see Table 2).

**Table 2:** Meta-regression models that predict lipid change

Lipids			*R*^2^	*P*	*I*^2^
TC Aerobic	Session Freq.	– 7.68	33%	0.003	46%
	Program (Wks)	– 0.51			
	Participant No	0.30			
	Constant.	14.39			
HDL Aerobic	Session Time	2.11	11%	0.013	70%
	Constant	– 1.42			
LDL Aerobic	Age	0.25	13%	0.003	79%
	Participant No.	0.83			
	Constant	2.08			

*HDL* high-density lipoprotein cholesterol, *LDL* low-density lipoprotein cholesterol, *TC* total cholesterol

### Recommendations for Further Trial Work on Exercise Training and Dyslipidemia

Despite the TSA revealing adequate information size for all five lipid outcomes, there remains a need for large, RCTs studying ExTr effects on DS. The justification for this lies in the fact that only four studies had > 100 participants in both exercise and control groups and the TSA analysis ignored a number of smaller studies. A novel approach could be a wait-listed control design to mitigate confounding and perhaps avoid overfitting of regression analyses. This research field has flaws because most studies exhibit small sample sizes and generally lacked robust design features. Our most interesting finding is that TC, LDL, TGD and VLDL did not respond to RT while HDL did. Thus future work should focus on optimal exercise programming for different types of DS, such as those shown in Table 3.

**Table 3:** Exercise program recommendations based upon dyslipidemia type

Type	HDL	LDL	Triglycerides	Recommendation
General dyslipidemia	Low	High		Aerobic and resistance training
Hyperlipidemia		High		Aerobic training
Hypoalphalipoproteinemia	Low			Aerobic and resistance training
Mixed hyperlipidemia	Low	High	High	Aerobic and resistance training
Hypertriglyceridemia	Low	High	High	Aerobic and resistance training

*HDL* high-density lipoprotein cholesterol, *LDL* low-density lipoprotein cholesterol

## Conclusions

ExTr produces small, but favourable, changes in lipid profile. TSA revealed the information size is sufficient. Combined training appears optimal. The size of change elicited may be helpful for achieving primary CVD prevention, independent of medical therapy. Different types of DS may require small adjustments to exercise program variables.

## Supplementary Information

Below is the link to the electronic supplementary material.

Supplementary file1 (DOCX 4030 KB)
