# Supplementary material for: The Effect of Exercise Training on Blood Lipids: A Systematic Review and Meta-analysis
Source: Sports Med. 2024 Sep 27;55(1):67–78. doi: 10.1007/s40279-024-02115-z (PMC11787149; doi:10.1007/s40279-024-02115-z)
Supplement: Supplementary file 1 — Supplementary file1 (DOCX 4030 KB) [file 40279_2024_2115_MOESM1_ESM.docx]

**Supplementary Files**

**The effect of exercise training on blood lipids: A systematic review and meta-analysis.**

**Running Title: Exercise Training for Dyslipdiemia**

Neil A. Smart^1^, David Downes ^1^, Tom van der Touw ^1^, Swastika Hada ^2^, Gudrun Dieberg^1^, Melissa J Pearson^1^, Mitchell Wolden^1,3^, Nicola King^4^, Stephen PJ Goodman^1^.

1. Dept. Exercise Physiology, University of New England, Armidale NSW 2351, Australia

2. Department of Pharmacy, Kathmandu University, JG9Q+PGG, Dhulikhel 45200, Nepal

3. Jamestown University, Dept. Physical Therapy, Fargo ND, USA

4. University of Plymouth, School of Biomedical Sciences, Devon, UK

**Address for Correspondence:**

**Professor Neil A. Smart**

School of Science and Technology, University of New England, Armidale NSW 2351, Australia

Email:nsmart54@gmail.com

**CONTENTS**

**FIGURES**

**Figures S1-S5 Funnel Plots**

**Figures S6a Trial Sequence Analysis (TSA) for Conventional Boundary Analyses**

**Figure S6b. Default Settings for TSA Adjusted Boundary Analyses**

**Figure S6c. Default Settings for TSA Law of Iterated Logarithm (LIL) Analyses**

**Figures S7-S15. Adjusted Boundary and LIL Analyses for TC, HDL, LDL, VLDL and TGD**

**TABLES**

**Table S1. Search Terms**

**Table S2. Comparison of Random Effects Models**

**Table S3. Excluded randomized, controlled trials**

**Table S4. Included Study Characteristics Studies reporting each outcome, all exercise types.**

**Table S5. Study quality assessment using TESTEX scale**

**References for Excluded and Included Studies**

**NB**

Risk of bias figures can be found via the following link [https://osf.io/dbeh9](HTTPS://OSF.IO/DBEH9) as the number of studies meant the font was too small to be presented here.

**FUNNEL PLOTS**

**Figure S1. Total Cholesterol**

**
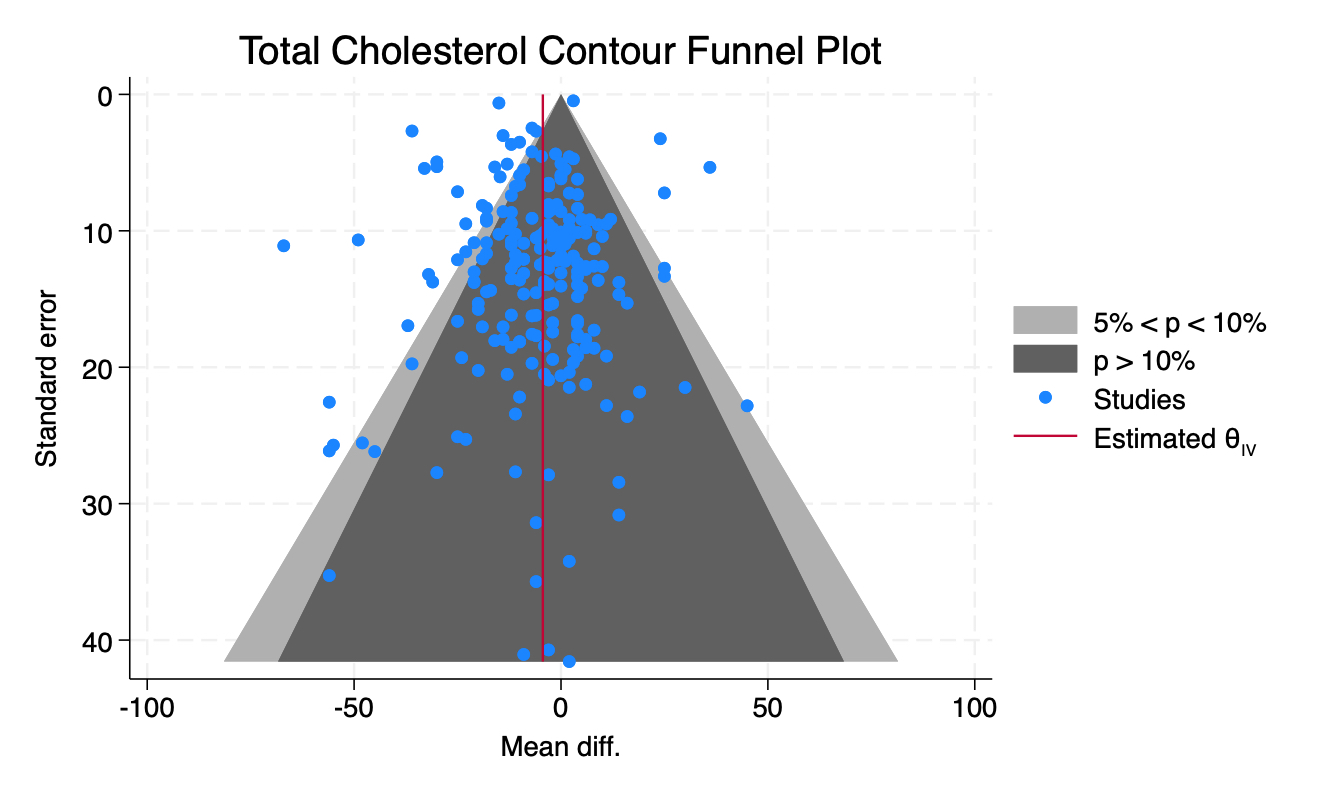
**

**Figure S2. HDL Funnel Plot**

**
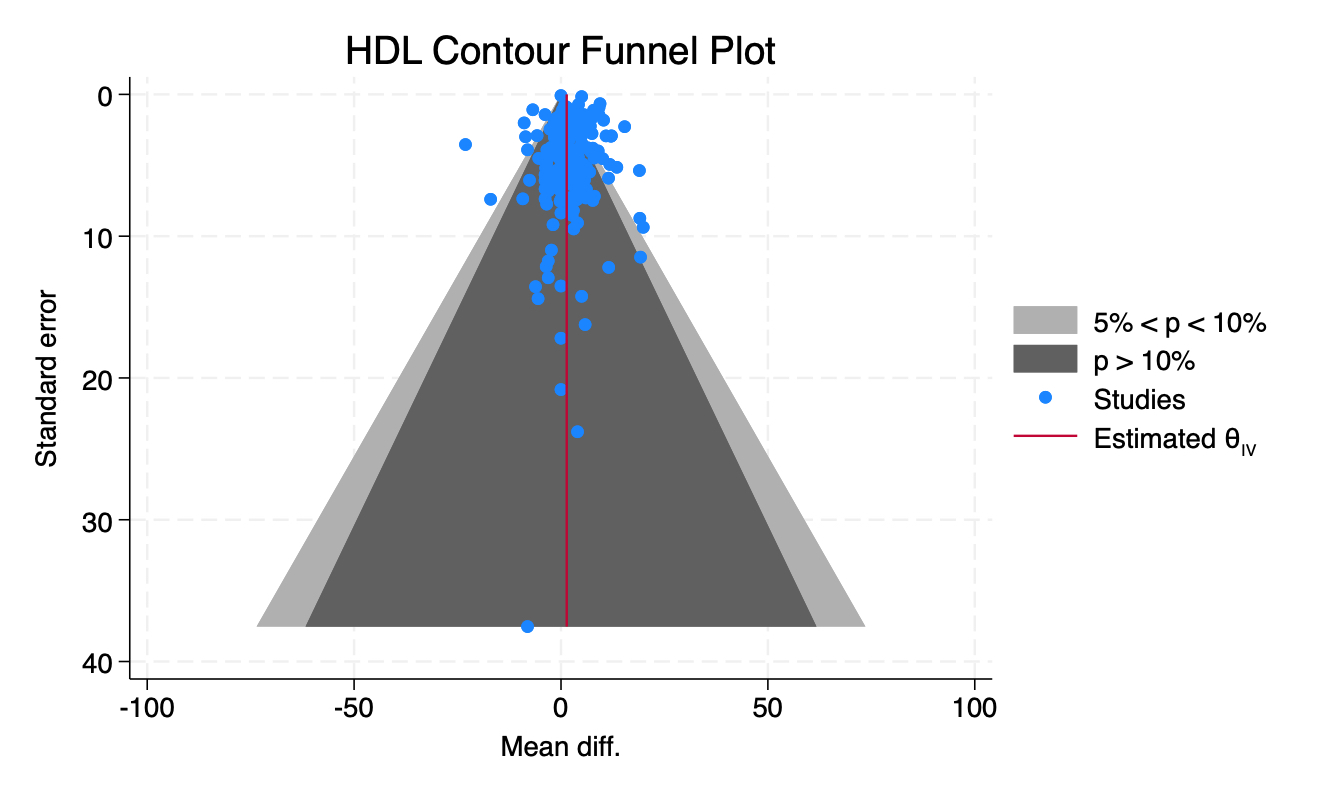
**

**Figure S3. LDL Funnel Plot**

**
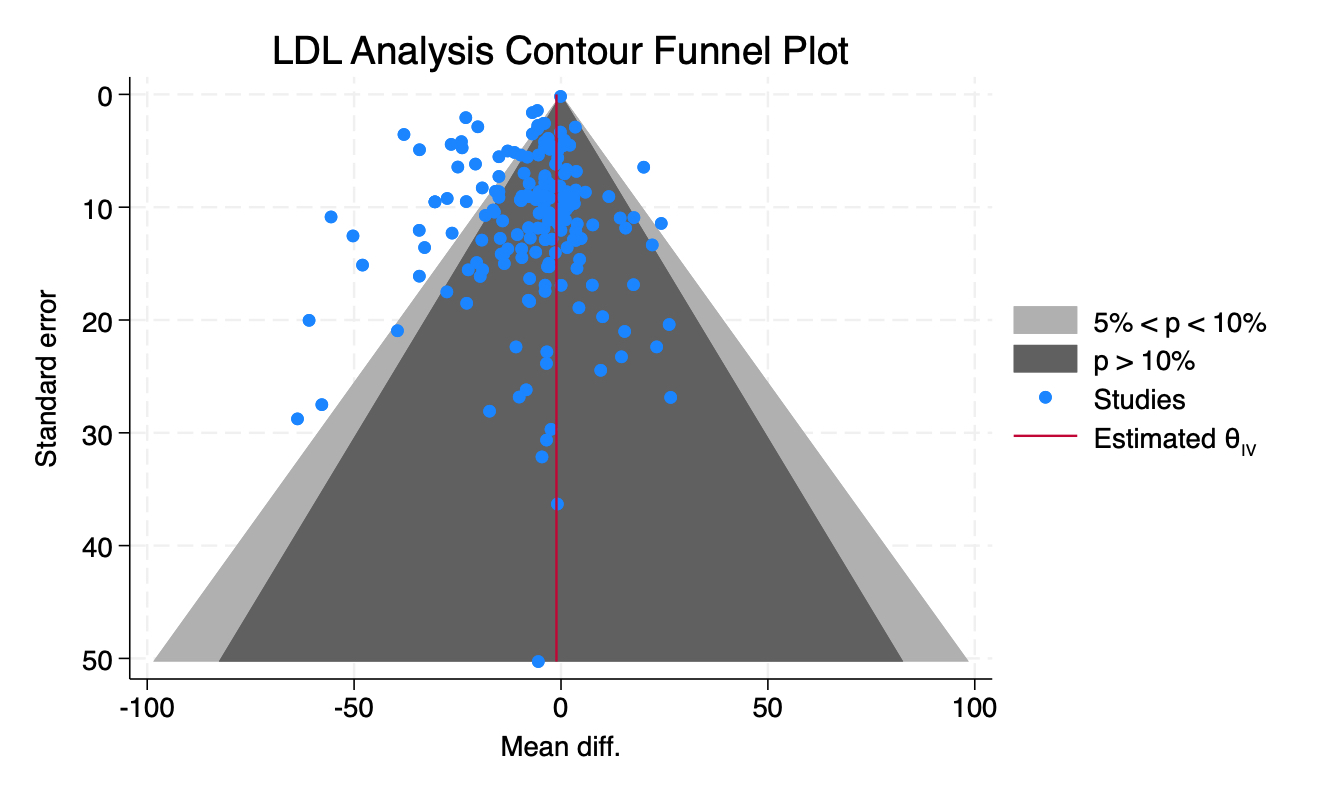
**

**Figure S4. Triglycerides Funnel Plot**

**
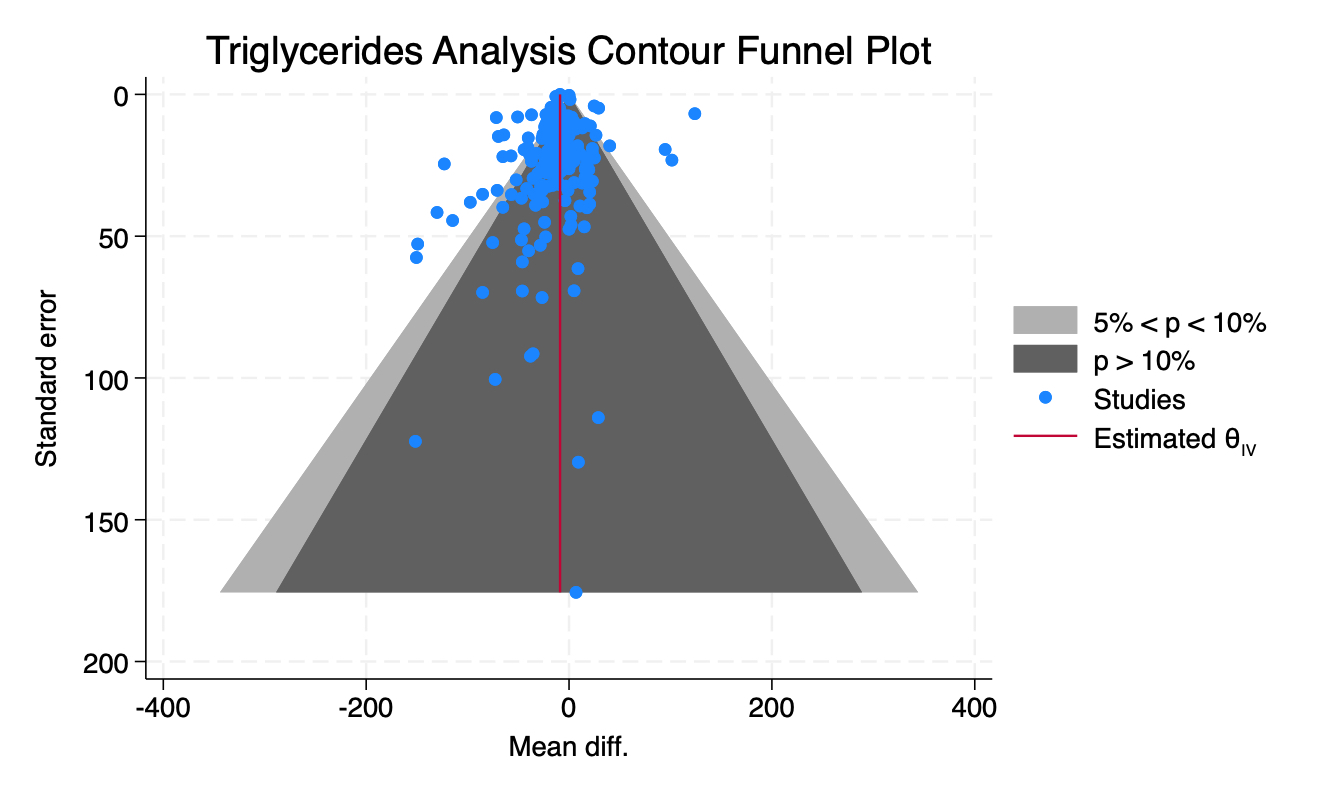
**

**Figure S5. VLDL Funnel Plot**

**
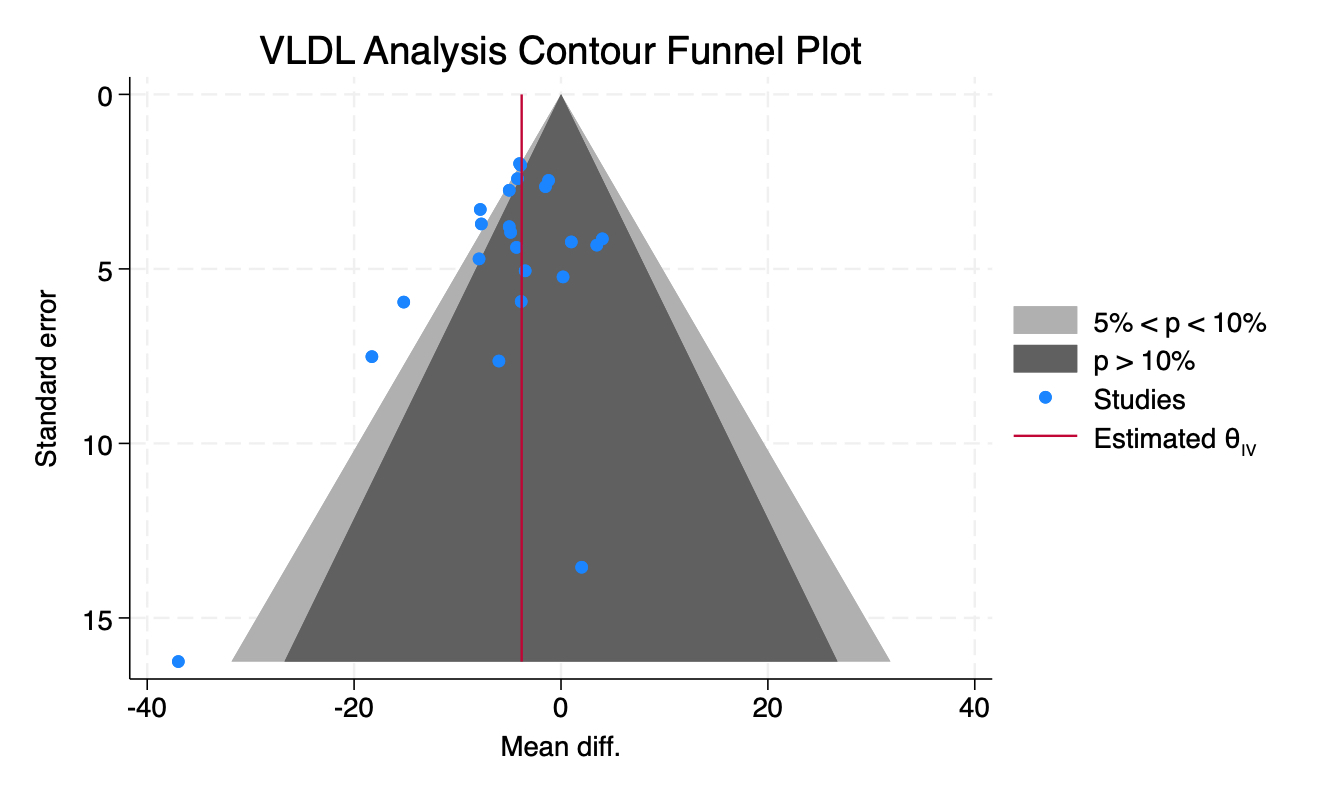
**

**Figure S6a. Default Settings for TSA Conventional Boundary Analyses**

**
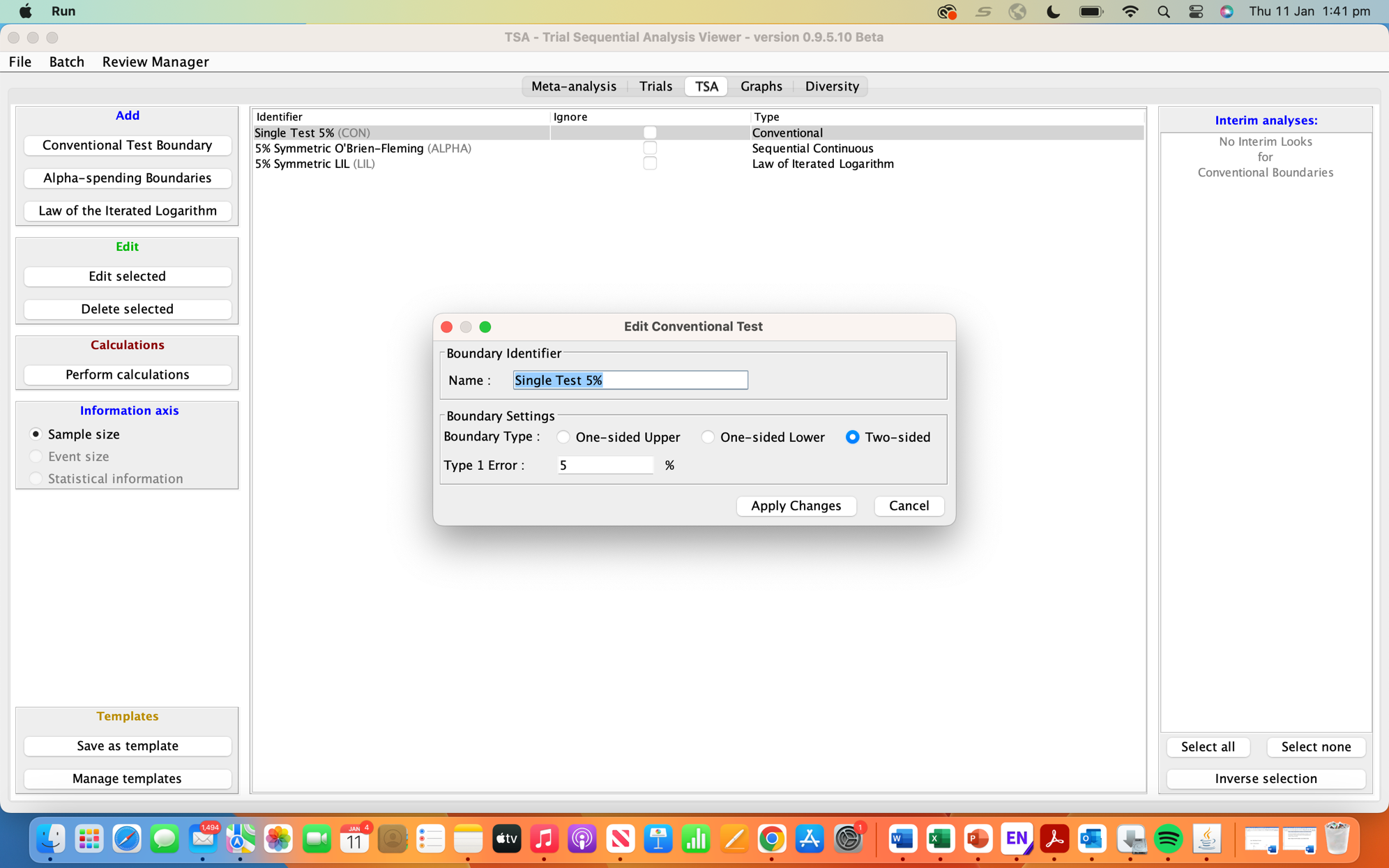
**

**Figure S6b. Default Settings for TSA Adjusted Boundary Analyses**

**
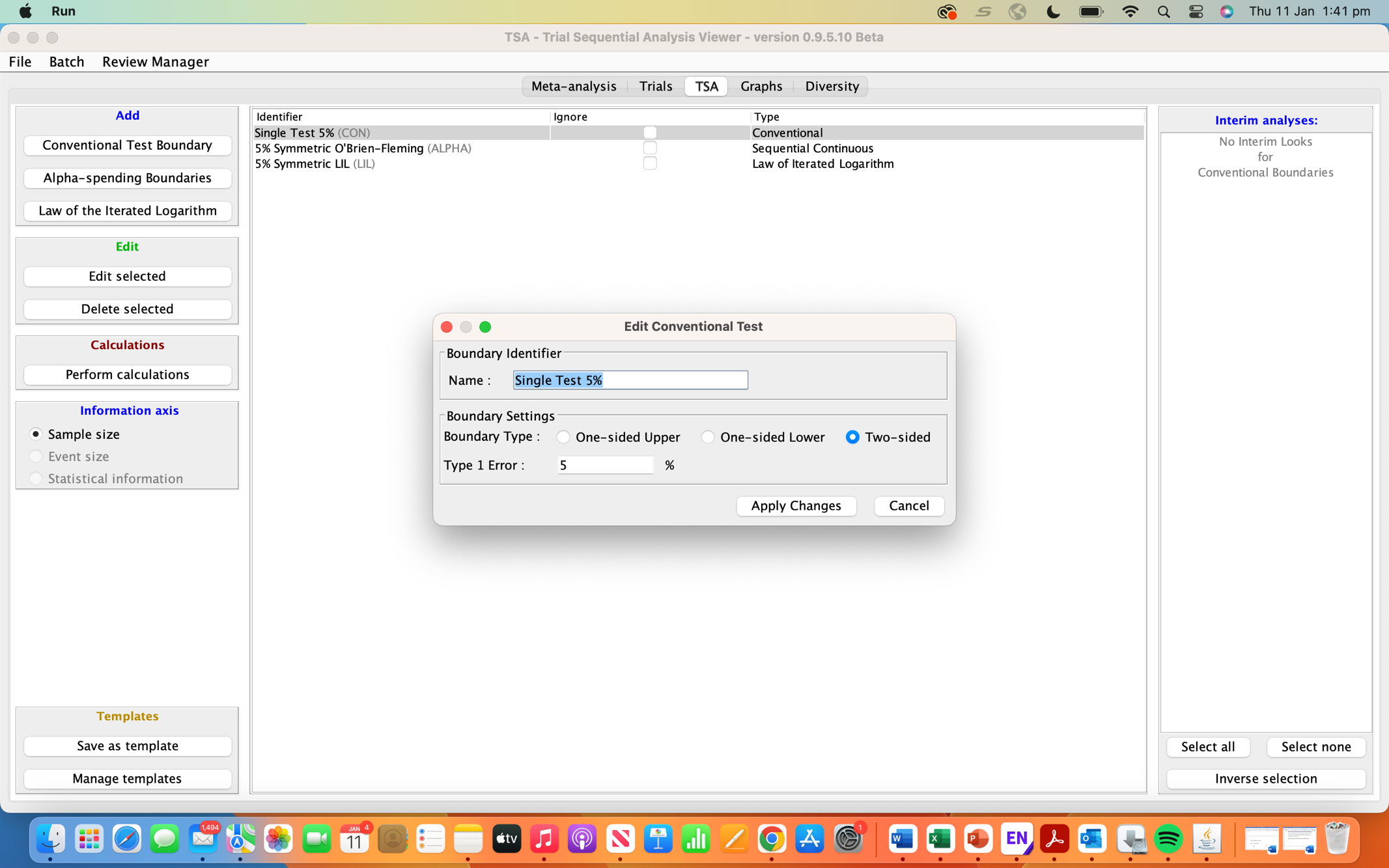
**

**Figure S6c. Default Settings for TSA Law of Iterated Logarithm (LIL) Analyses**

**
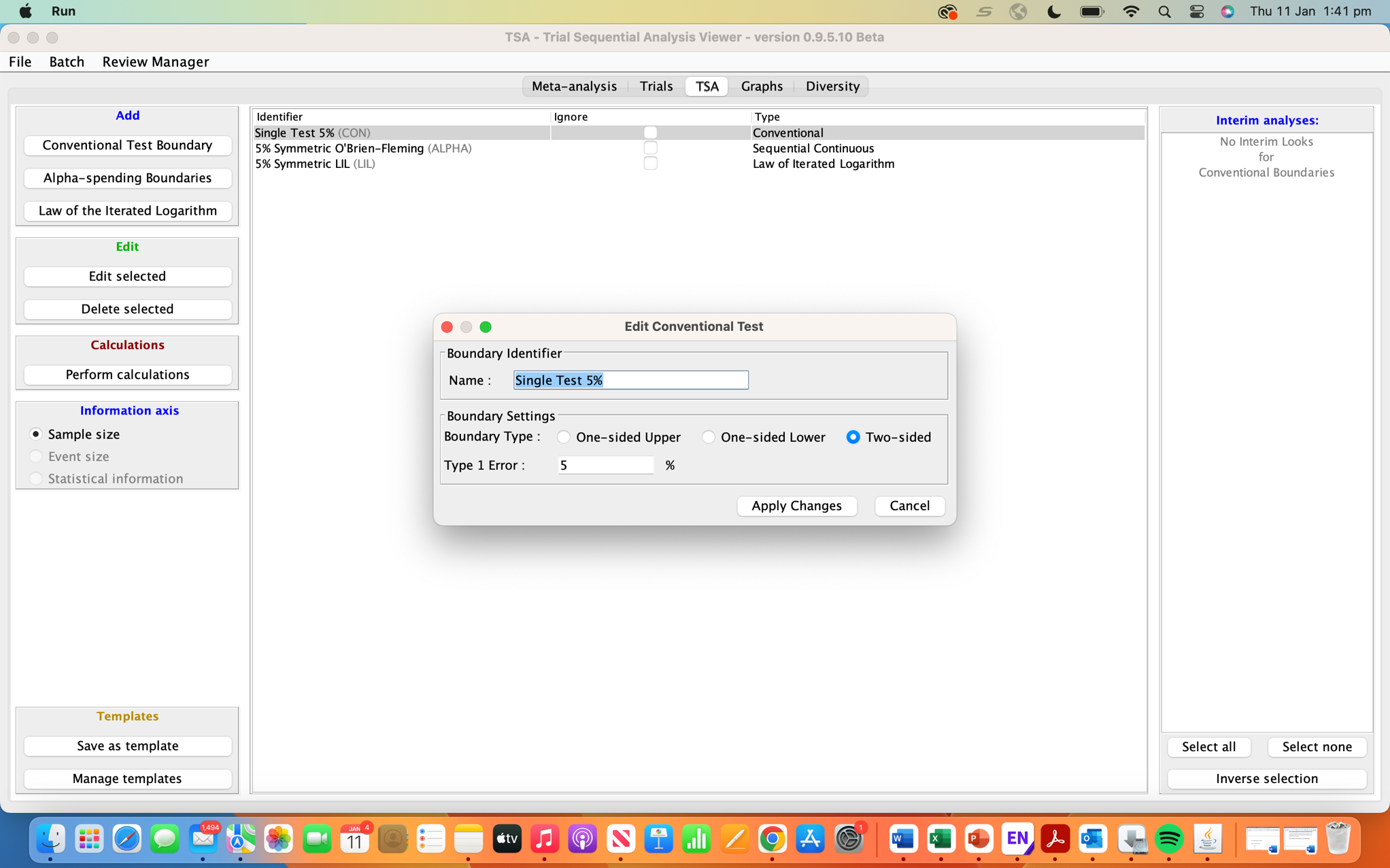
**

**Figure S7. TC Adjusted Boundaries**

**
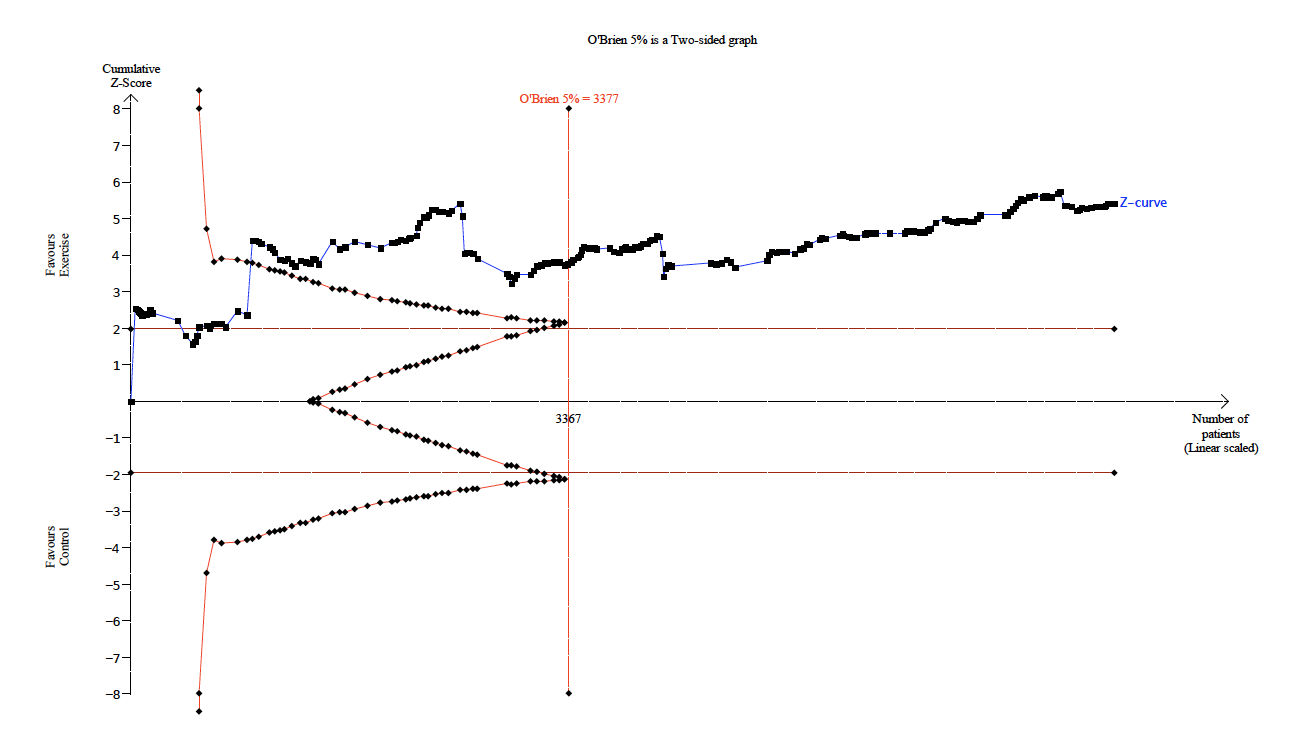
**

**Figure S8. TC Penalized Tests**

**
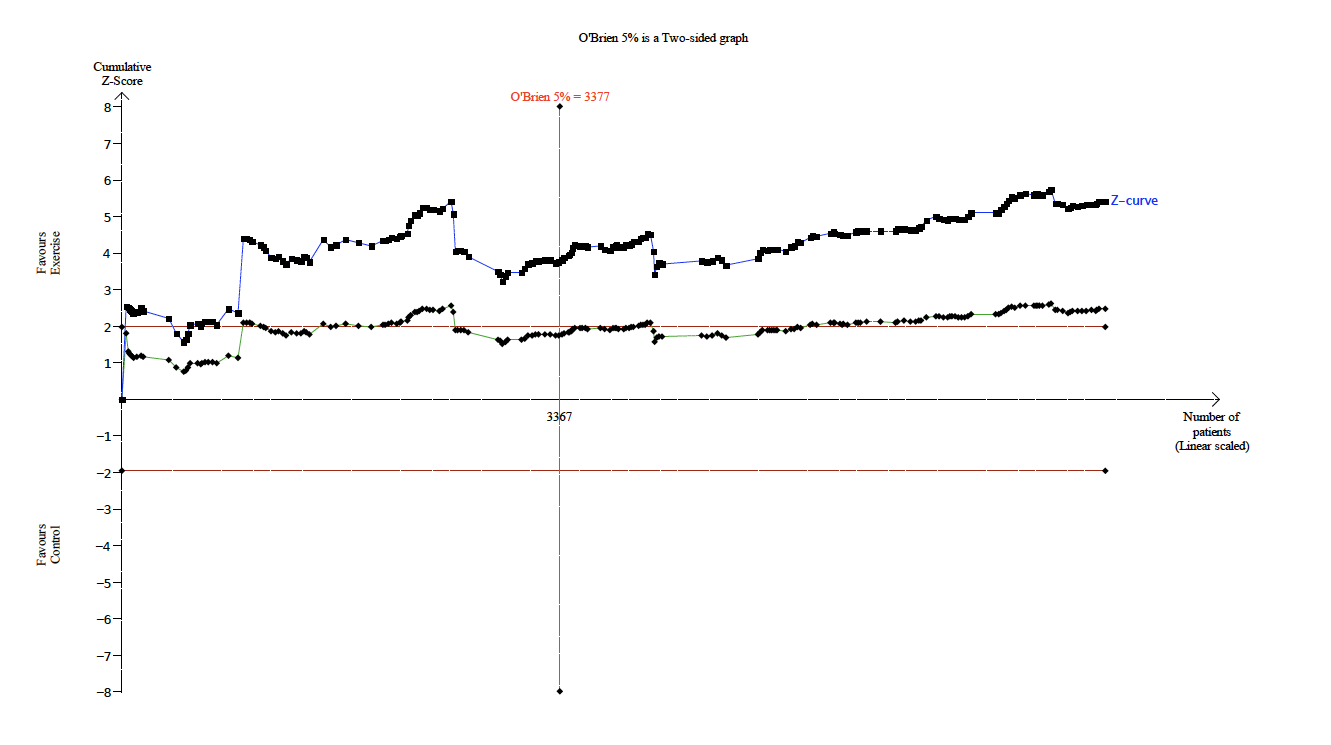
**

**Figure S9. HDL Adjusted Boundaries**

**
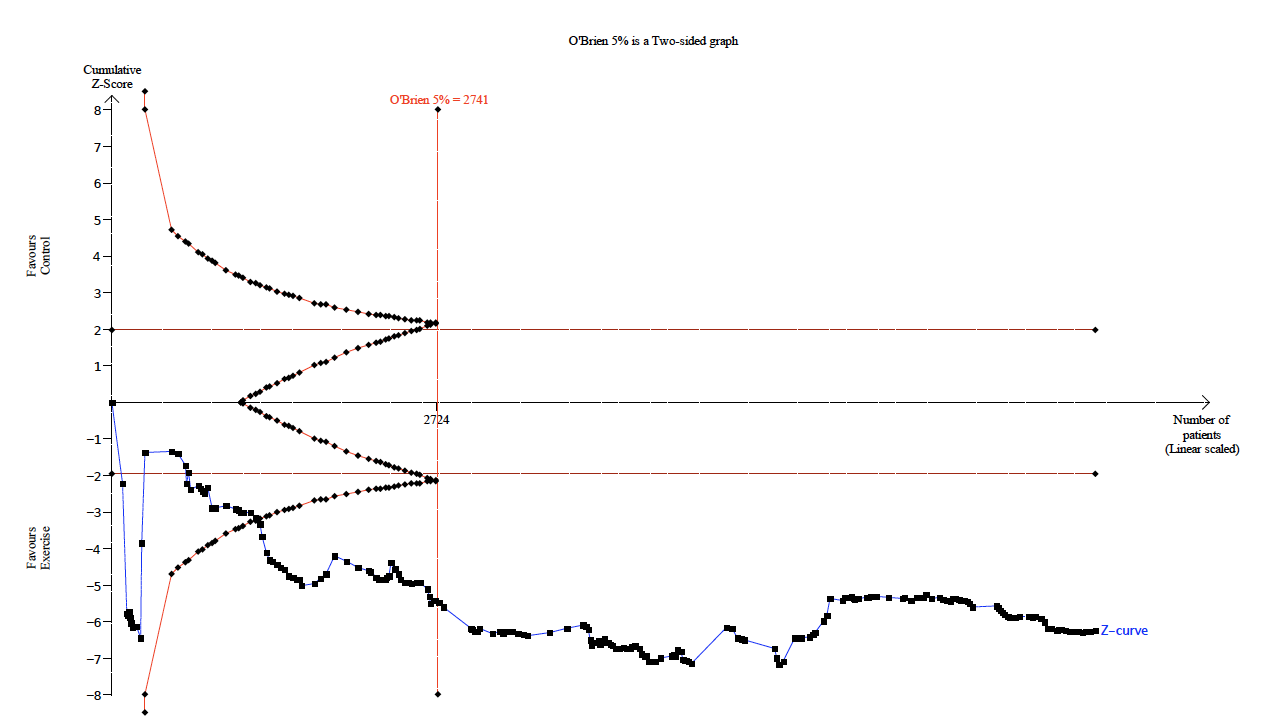
**

**Figure S10. HDL Penalized Tests**

**
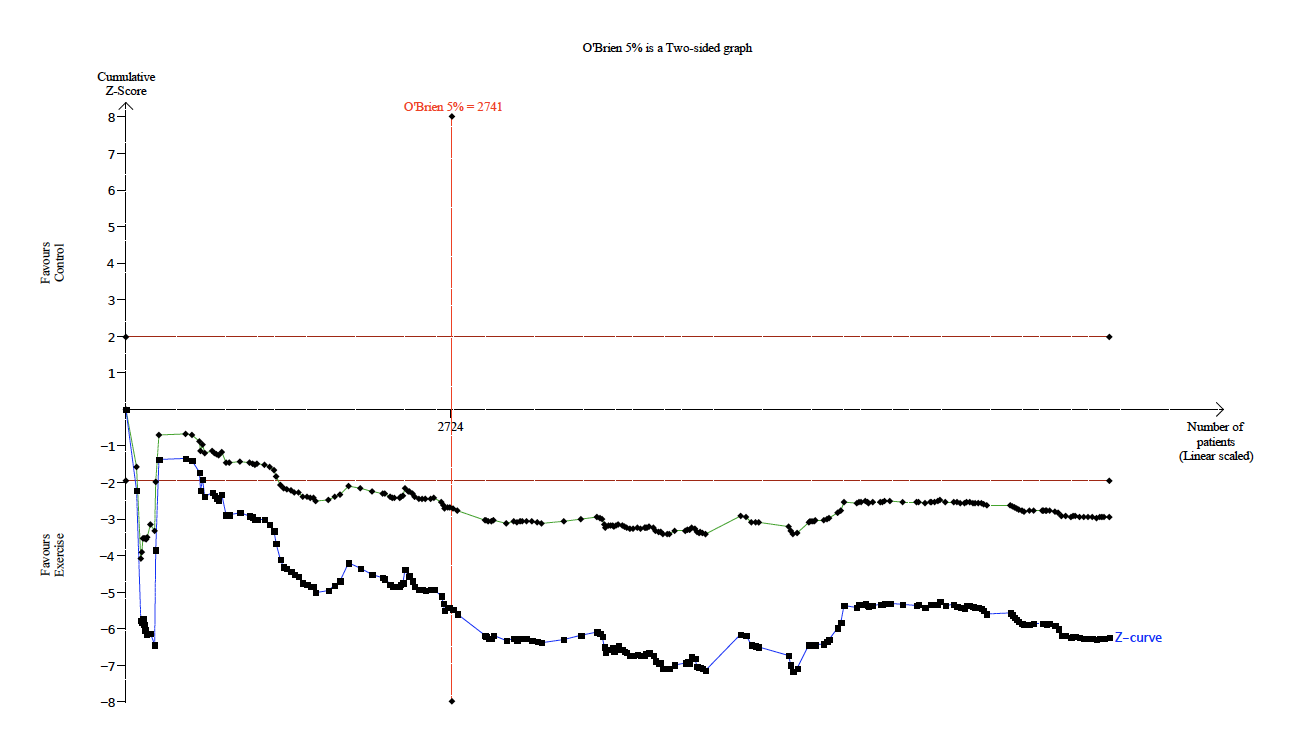
**

**Figure S11. LDL Adjusted Boundaries**

**
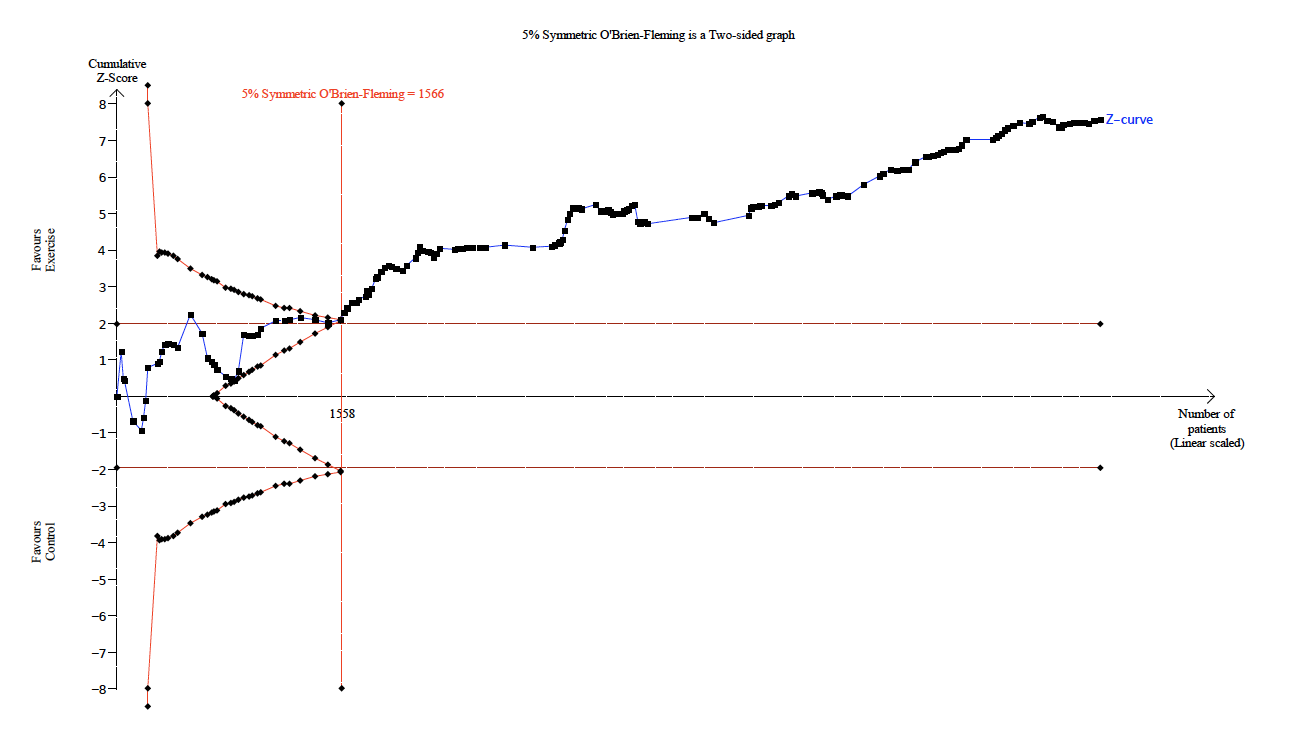
**

**Figure S12. LDL Penalized Tests**

**
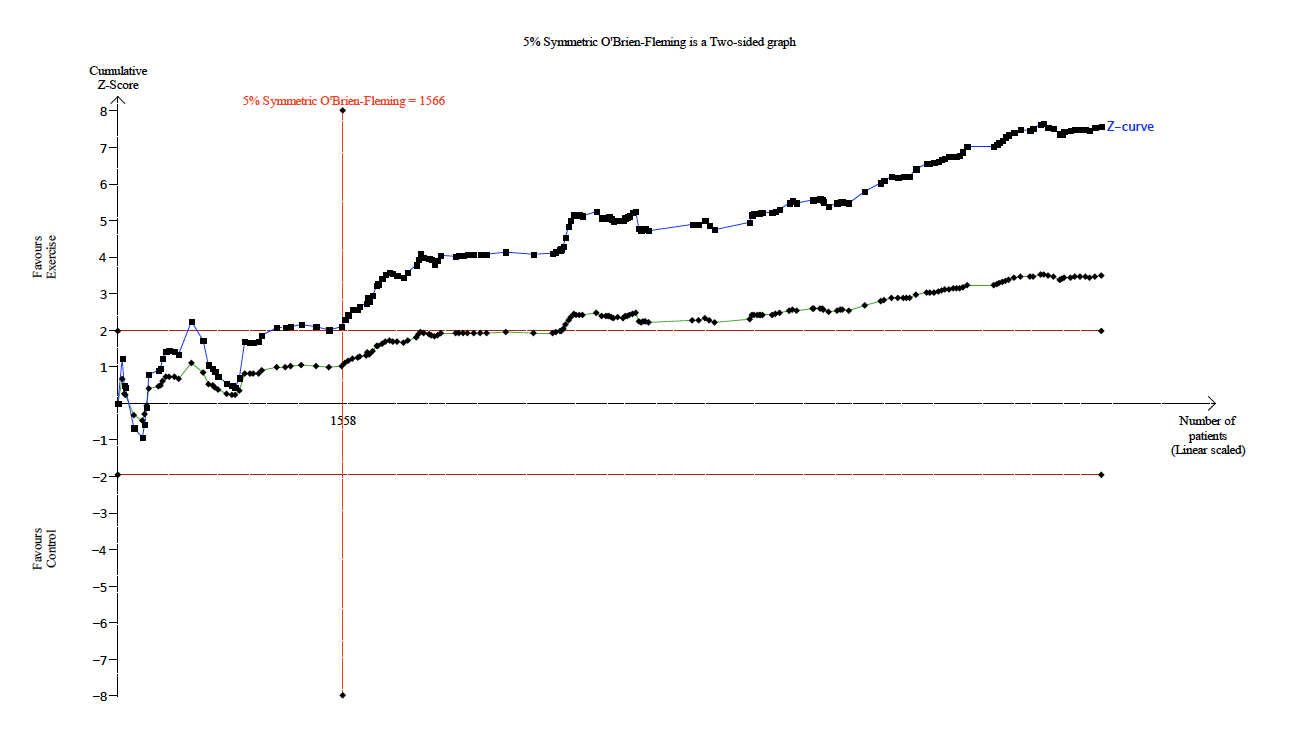
**

**Figure S13. VLDL Adjusted Boundaries**

**
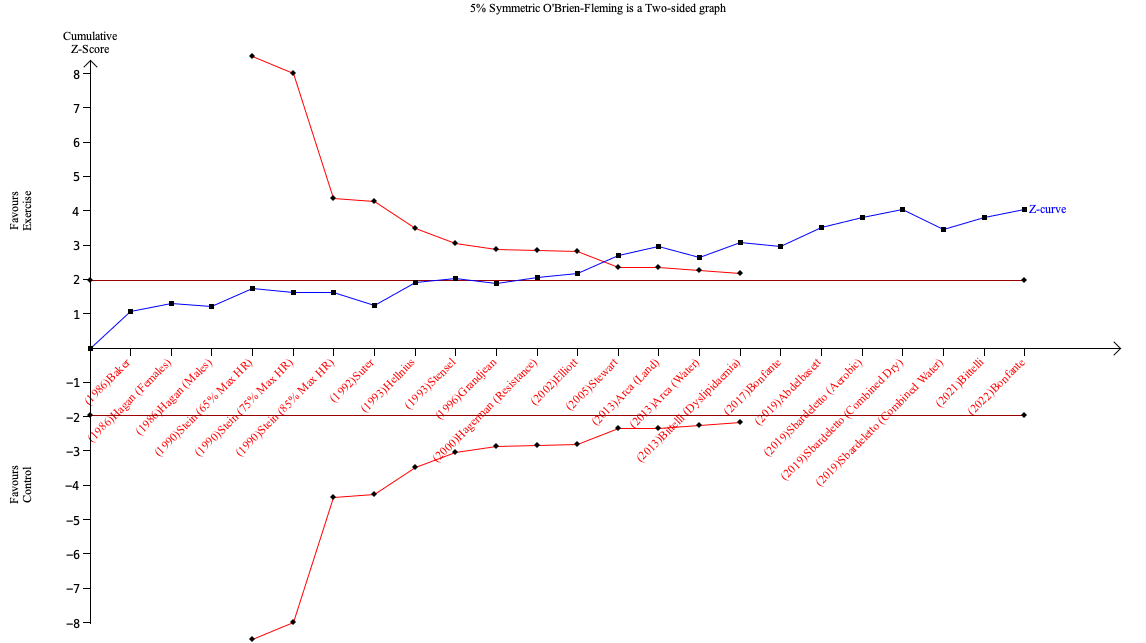
**

**Figure S14. VLDL Penalized Tests**

**
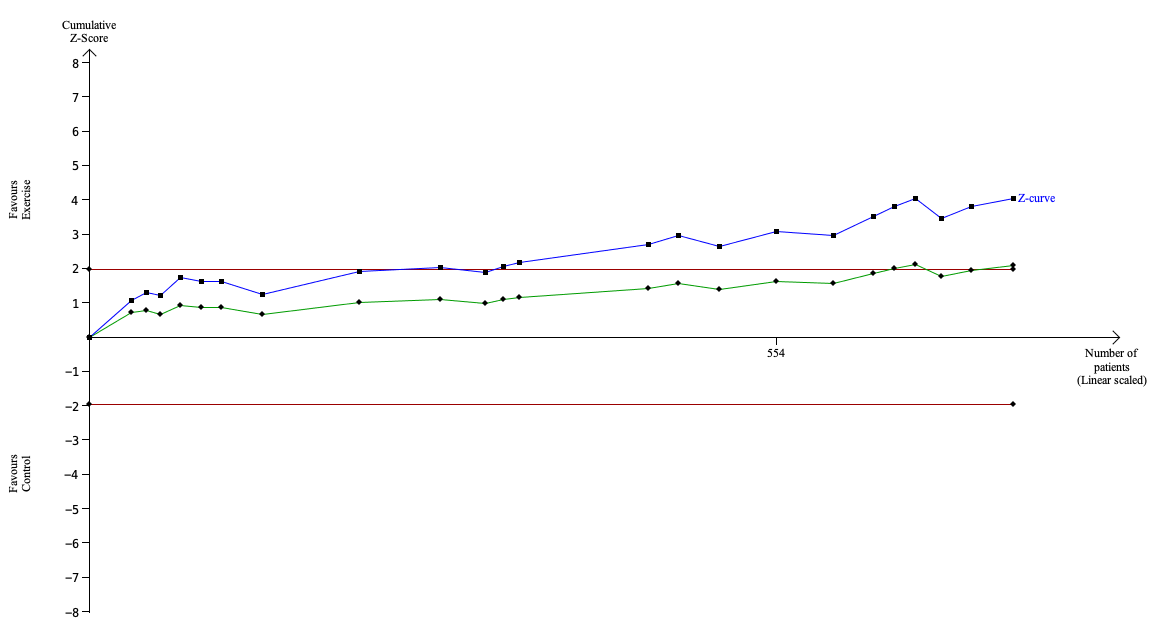
**

**Figure S15. TGD Adjusted Boundaries**

**
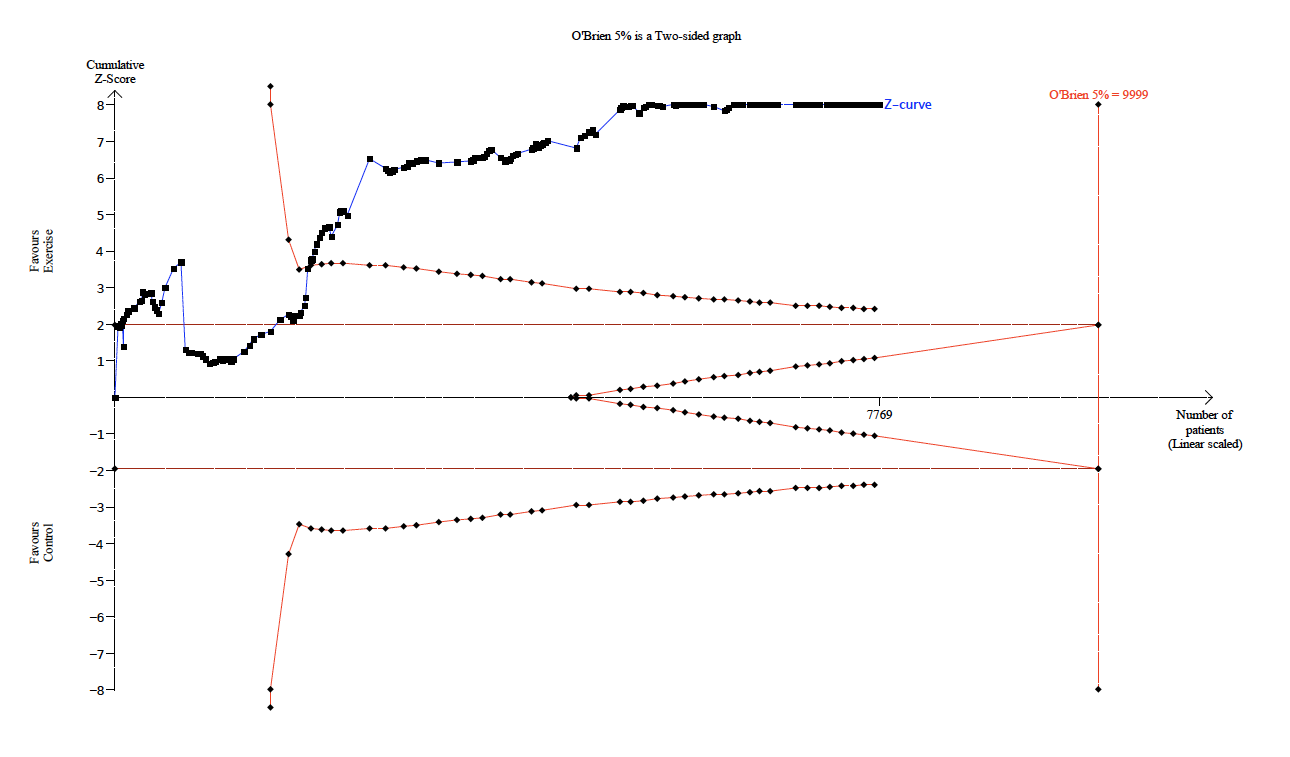
**

**Figure S16. TGD Penalized Tests**

**
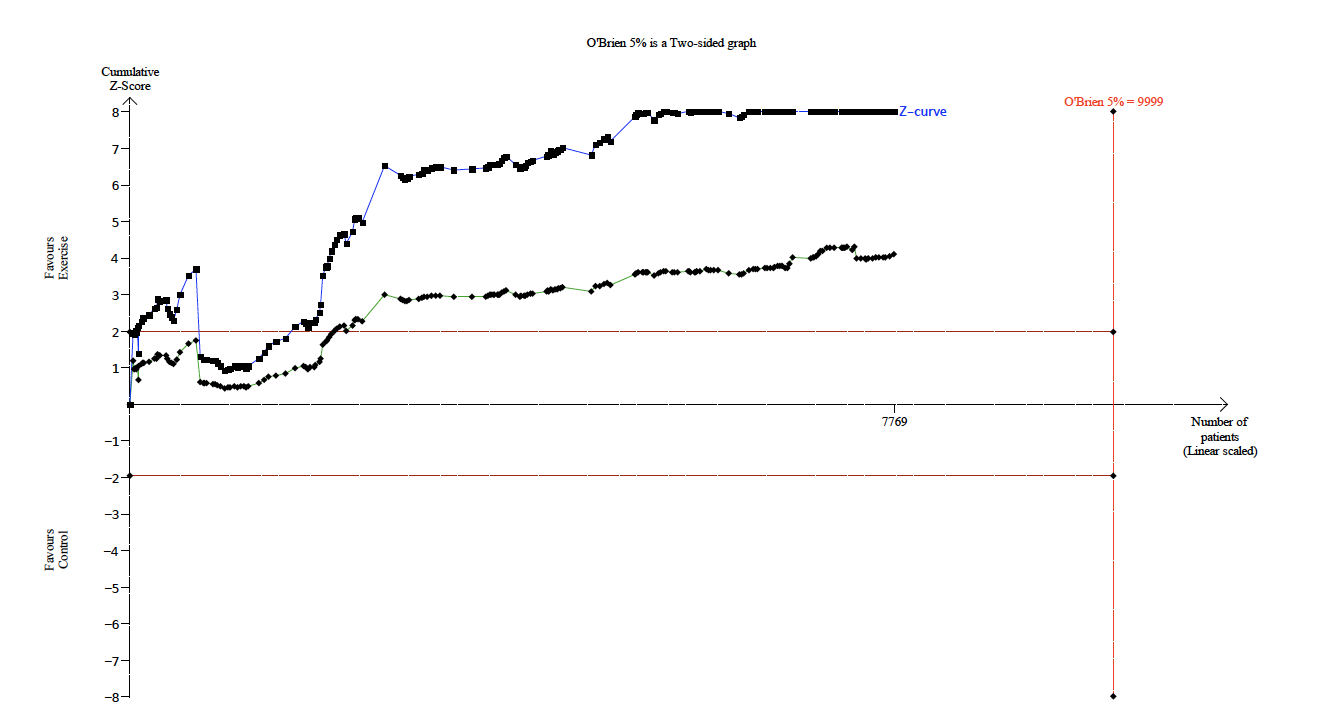
**

**TABLES**

**TABLE S1. SEARCH TERMS**

| Items | Query |
| --- | --- |
| #5 | Search: ((((((((aerobic exercise[Title/Abstract]) OR (resistance exercise[Title/Abstract])) OR (exercise[Title/Abstract])) OR (training[Title/Abstract])) OR (physical activity[Title/Abstract])) OR (isometric exercise[Title/Abstract])) OR (endurance training[Title/Abstract])) OR (strength training[Title/Abstract])) AND (dyslipidemia[Title/Abstract])) OR (hypercholesterolemia [Title/Abstract])) OR (hypertriglyceremia [Title/Abstract])) |
| #4 | Search: ((((((((aerobic exercise[Title/Abstract]) OR (resistance exercise[Title/Abstract])) OR (exercise[Title/Abstract])) OR (training[Title/Abstract])) OR (physical activity[Title/Abstract])) OR (endurance training[Title/Abstract])) OR (strength training[Title/Abstract])) AND (((((dyslipidemia [Title/Abstract]) OR (hypercholesterolemia[Title/Abstract])) OR (hypertriglyceremia [Title/Abstract])) |
| #3 | Search: ((((((((aerobic exercise[Title/Abstract]) OR (resistance exercise[Title/Abstract])) OR (exercise[Title/Abstract])) OR (training[Title/Abstract])) OR (physical activity[Title/Abstract])) OR (endurance training[Title/Abstract])) OR (strength training[Title/Abstract])) AND (((((dyslipidemia[Title/Abstract]) OR (hypercholesterolemia[Title/Abstract])) OR (hypertriglyceremia[Title/Abstract])) |
| #2 | Search: ((((dyslipidemia[Title/Abstract]) OR (hypercholesterolemia[Title/Abstract])) OR (renin[Title/Abstract])) OR (hypertiglyceremia[Title/Abstract])) |
| #1 | Search: (((((((aerobic exercise[Title/Abstract]) OR (resistance exercise[Title/Abstract])) OR (exercise[Title/Abstract])) OR (training[Title/Abstract])) OR (physical activity[Title/Abstract])) OR (endurance training[Title/Abstract])) OR (strength training[Title/Abstract]) |

**TABLE S2. Comparison of the Der Simonian-Larid and Sidik-Jonkman Random Effects Models.**

| **Model** | **Total Cholesterol** | **HDL-C** | **LDL-C** | **VLDL-C** | **TGD** |
| --- | --- | --- | --- | --- | --- |
| **Der-Simonian-Laird** | **-6.32 (-6.32, -6.32)** | **2.16 (1.48, 2.83)** | **-7.05 (-8.87, -5.22)** | **-4.13 (-6.13, -2.13)** | **-10.57 (-12.83, -8.2)** |
| **Sidik- Jonkman** | **-6.32 (-6.32, -6.32)** | **2.13 (1.4, 2.86)** | **-6.98 (-9.2, -4.77)** | **-4.64 (-7.71, -1.51)** | **-12.52 (-16.37, -8.65)** |

**TABLE S3. EXCLUDED RANDOMIZED, CONTROLLED TRIALS OF EXERCISE TRAINING IN ADULTS.**

| **Study** | **Reason(s)** |
| --- | --- |
| Aekplakorn 2019(1) | Periodic exercise sessions. Cluster randomization. |
| Akkurt 2017 (2) | Subjects had spinal cord injury |
| Aller 2015 (3) | Control group given supplements and dietary intervention |
| Armitage 2021(4) | No sedentary control group |
| Aro 2019(5) | Control group received dietary intervention |
| Balducci 2010(6) | Control group received counselling. Cluster randomization. |
| Balk-Moller 2017(7) | Study tested a phone app. Cluster randomization. |
| Baltic 2015(8) | Subjects had peripheral vascular disease |
| Banz 2003(9) | No sedentary control |
| Barr 1991(10) | No sedentary control |
| Behall 2003(11) | No sedentary control |
| Benatti 2017(12) | Acute study, no training program |
| Besnier 2015(13) | No sedentary control |
| Bidwell 2014(14) | Combined dietary and exercise intervention |
| Blackford 2016(15) | Combined dietary and exercise intervention |
| Bombardier 2020 (16) | Spinal cord injury. |
| Bonato 2020 (17) | Participants had HIV |
| Boer 2014(18) | Subjects had intellectual disability |
| Borges 2019(19) | No control group. Dietary intervention control group |
| Botero 2014(20) | No sedentary control group |
| Bozetto 2013 (21) | No sedentary or diet free control group |
| Brobakken 2019 (22) | Participants had schizophrenia |
| Buchan 2013(23) | Child Participants |
| Butelli 2021(24) | Not aerobic or resistance exercise |
| Cardenas-Sanchez 2016(25) | Child Participants |
| Cardoso 2020(26) | No lipid data |
| Canuto 2019(27) | Concurrent dietary intervention. Education Only. |
| Chang 2020(28) | Participants had breast cancer |
| Charactcahroenwitthaya 2021(29) | No sedentary control. Concurrent dietary intervention. |
| Chiavarino 2016(30) | No exercise intervention |
| Choo 2014(31) | No sedentary control |
| Chung 2017(32) | Single session exercise |
| Ciolac 2011(33) | No lipid data |
| Clark 2019(34) | Pregnant participants |
| Coen 2015(35) | Post-gastric by-pass participants |
| Cox 1993(36) | Concurrent alcohol restriction intervention |
| Cox 2010(37) | No sedentary control group. |
| Crouter 2015(38) | Child participants |
| Cullberg 2010(39) | No sedentary control |
| Da Silva Soares 2022(40) | Concurrent dietary intervention |
| Dai 2019(41) | No sedentary control |
| Dawson 2018(42) | Prostate cancer participants |
| De Meirelles 2014(43) | Heart disease participants |
| De Sousa 2014(44) | Concurrent dietary therapy |
| De Strijker 2018(45) | No sedentary control |
| Del Pozo-Cruz 2014(46) | Whole body vibration, no exercise training |
| Delevatti 2015(47) | No sedentary control |
| Delgado-Floody 2019(48) | No sedentary control |
| Dennis 2013(49) | Child participants |
| Dipietro 2006(50) | No lipid data |
| Duncan 2005(51) | No sedentary control |
| Ehret 2021(52) | Breast cancer participants |
| Elme 2013(53) | Breast cancer participants |
| Espeland 2013(54) | Concurrent dietary therapy |
| Farag 2019(55) | Concurrent dietary therapy |
| Faria 2020(56) | Child participants |
| Faulkener 2022(57) | Stroke participants |
| Fernandez-Ruiz 2021(58) | Child participants |
| Fisher 2015(59) | No sedentary control |
| Frederix 2015(60) | Heart disease participants |
| Freedland 2019(61) | Prostate cancer participants |
| Galvao 2013(62) | Prostate cancer, androgen suppression treatment |
| Garavelo 2019(63) | No lipid data |
| Garner 2017(64) | Dietary or exercise education only |
| Giallauria 2014(65) | Breast cancer participants |
| Gonzalez-Ruiz 2021(66) | Child participants |
| Gouveia 2021(67) | No sedentary control |
| Hagner-Derengowska 2015(68) | No lipid data |
| Halverstadt 2003(69) | No sedentary control |
| Harreiter 2019(70) | Pregnant participants |
| Hiruntrakul 2010(71) | Only one week of exercise training |
| Hojan 2017(72) | Prostate cancer participants |
| Howden 2013(73) | Chronic kidney disease participants |
| Hsu 2021(74) | No sedentary control |
| Hvid 2015(75) | Participants with prostate cancer |
| Inoue 2014(76) | Child participants |
| Jackson 2017(77) | Selective reporting, no post-exercise lipid control data reported |
| Jones 2014(78) | Prostate cancer participants |
| Joseph 1999(79) | No sedentary control |
| Joubert 2020(80) | Stroke participants |
| Juliana 2017(81) | Consecutive pre-dietary therapy |
| Jurczak 2014(82) | Heart disease participants |
| Kanaya 2014(83) | Not traditional aerobic or resistance exercise |
| Karstoft 2013(84) | Control participants re-randomized to exercise |
| Katzel 1997(85) | No sedentary control |
| Keadle 2014(86) | Control participants re-randomized to exercise |
| Kerling 2015(87) | No sedentary control |
| Kilen 2021(87) | No sedentary control |
| Kileen 2022(88) | Pregnant participants |
| Kim 2015(89) | Spinal cord injury participants |
| Kim 2022(90) | Pregnant participants |
| King 1995(91) | No sedentary control |
| Kirk 2014(92) | Stroke participants |
| Kleinloog 2022(93) | Crossover design meant relevant lipid data not available |
| Kokkinos 1998(94) | No lipid data |
| Kong 2016(95) | No sedentary control, only moderate v high intensity |
| Kono 2013(96) | Stroke participants |
| Kraemer 1997(97) | Concurrent dietary therapy |
| Kraemer 1999(98) | Concurrent dietary therapy |
| Krogh 2012(99) | No sedentary control |
| Kuo 2020(100) | Inspiratory muscle training |
| Lee 2005(101) | Article in Korean, cannot extract data |
| Lima 2020(102) | Child participants |
| Lindheim 1994(103) | Abstract only |
| Liu 2021(104) | No lipid data |
| Liu 2022(105) | Cardiovascular disease participants |
| Lockard 2022(106) | No sedentary control |
| Lopes 2016(107) | Child participants |
| Mackay-Lyons 2022(108) | Cardiovascular disease participants |
| Malmo 2016(109) | Cardiovascular disease participants |
| Marcotte-Chenard 2021(110) | No sedentary control |
| Matsugaki 2017(111) | No sedentary control, comparison of two exercise groups |
| Matsuo 2015(112) | No sedentary control |
| McAuley 2002(113) | No sedentary control |
| McCormack 2014(114) | Child Participants/Combined dietary/exercise |
| Meng 2022(115) | Child Participants |
| Michael 2021(116) | No sedentary control |
| Moholdt 2021(117) | Less than 2 weeks exercise program |
| Moore 2015(118) | Participants post-stroke |
| Moro 2020(119) | No sedentary control, comparison of two exercise groups |
| Muros 2013(120) | Child participants |
| Nah 2019(121) | Concurrent dietary therapy |
| Nicklas 2009(122) | Concurrent dietary therapy |
| Nieman 1998(123) | Concurrent dietary therapy |
| Noites 2020(124) | No sedentary control |
| Nono 2022(125) | No lipid data |
| Nooijen 2017(126) | Subjects had spinal cord injury |
| Nybacka 2017(127) | Concurrent dietary therapy |
| O’Doherty 2018(128) | Acute study, no exercise training program |
| Onagbiye et al 2016(129) | No control group. Pre- and post-training comparison only |
| Ordonez 2013(130) | Subjects had spinal cord injury |
| Pablos 2017(131) | Child participants |
| Paschali 2020(132) | No sedentary control |
| Pedersen 2015(133) | No sedentary control group |
| Pedersen 2019(134) | No sedentary control group |
| Poeta 2013(135) | Child participants |
| Ponjee 1995(136) | No training data |
| Prince 2018(137) | No sedentary control |
| Qazi 2021(138) | Concurrent dietary intervention |
| Ramirez-Velez 2020(139) | No sedentary control |
| Ratajczk 2019(140) | No sedentary control, comparison of two exercise groups |
| Rech 2019(141) | No sedentary control |
| Resaland 2017(142) | Child participants |
| Rivas 2019(143) | Less than 2 weeks exercise training |
| Rodriguez 2022(144) | Dietary intervention control |
| Rodriguez-Krause 2019(145) | No sedentary control, active control |
| Rosa 2021(146) | Participants with chronic kidney disease |
| Rosenberg 2018(147) | Concurrent diet/other therapy |
| Ruffino 2016(148) | No sedentary control |
| Ryan 2020(149) | No sedentary control, comparison of two exercise groups |
| Salahshoornezhad 2022(150) | Child participants |
| Scott 2018(151) | Exercise supplemented with testosterone |
| Seguin-Fowler 2020(152) | No lipid data |
| Seyam 2022(153) | Concurrent dietary intervention |
| Sheikholeslami 2011(154) | No sedentary control |
| Shepherd 2015(155) | No sedentary control |
| Siddiqui 2016(156) | Concurrent diet/other therapy |
| Sigal 2014(157) | Child participants |
| Sillanpaa 2010(158) | No lipid data/Concurrent dietary therapy |
| Simpson 2016(159) | Not exercise intervention |
| Sjoros 2018(160) | No sedentary control |
| Slentz 2005(161) | No lipid data |
| Slentz 2016(162) | No sedentary control |
| Solomon 2018(163) | Concurrent diet/other therapy |
| Song 2020(164) | Participants had cardiovascular disease |
| Sopko 1985(165) | Concurrent dietary intervention |
| Sparks 2013(166) | No lipid measures |
| Staiano 2018(167) | Child participants |
| Stavnsbo 2020(168) | Child participants |
| Sung 2012(169) | Not exercise training |
| Suzuki 2013(170) | Participants with cognitive impairment |
| Swoboda 2016(171) | Concurrent dietary intervention |
| Tamburus 2016(172) | People with cardiovascular disease |
| Tang 2014(173) | Stroke participants |
| Tarp 2018(174) | Child participants |
| Telford 2013(175) | Child participants |
| Terada 2012(176) | No sedentary control |
| Tillin 2019(177) | No sedentary control |
| Toledo 2008(178) | Concurrent dietary therapy |
| Toscano 2018(179) | Child participants |
| Uth 2018(180) | Prostate cancer participants |
| Uth 2020(181) | Breast cancer participants |
| Van Aggel-Leijssen 2001a(182) | No lipid data, concurrent dietary intervention |
| Van Aggel-Leijssen 2001b(183) | No lipid data, concurrent dietary intervention |
| Van Ryckeghem 2022(184) | No sedentary control, comparison of 2 exercise groups |
| Van Schijndel-Speet 2017(185) | Subjects had intellectual disability, cluster randomization |
| Vasconcellos 2016(186) | Child participants |
| Vella 2017(187) | No sedentary control, comparison of two exercise groups |
| Vissers 2010(188) | Used whole body vibration not exercise |
| Volpe 2008(189) | No sedentary control, stratified interventions |
| Wallman 2009b(190) | No sedentary control, concurrent dietary intervention |
| Warner 1989(191) | Concurrent dietary supplementation |
| Wasenius 2020(192) | No sedentary control |
| Weiss 2016(193) | Concurrent weight loss therapy |
| Wilund 2002a(194) | Matched controls assigned after initial randomization |
| Willund 2002b(195) | No randomization |
| Winn 2018(196) | No lipid data |
| Yates 2014(197) | Exercise education not exercise program |
| Yoshizawa 2009(198) | Concurrent drug therapy |
| Zaman 2021(199) | Study conducted at high altitude |
| Zanetti 2020(200) | HIV participants |
| Zheng 2015(201) | Post-stroke participants |
| Zou 2015(202) | Post-stroke participants |
| Zourladani 2015(203) | Pregnant women |

**TABLE S4. INCLUDED STUDY CHARACTERISTICS**

| **STUDY** | **YEAR** | **Mode** | **Freq./Week** | **Intensity** | **Session Time (Mins)** | **Program Time (Weeks)** |
| --- | --- | --- | --- | --- | --- | --- |
| Abdelbasett(204) | 2019 | Bike | 3 | High | 38 | 8 |
| Abdelbasset moderate (205) | 2020 | Bike | 3 | Moderate | 45 | 8 |
| Afzalpour vigorous(206) | 2008 | Run | 3 | Vigorous | 37.5 | 8 |
| Afzalpour moderate(206) | 2008 | Run | 3 | Moderate | 37.5 | 8 |
| Agner(207) | 2018 | Walk | 2 | Vigorous | 90 | 12 |
| Akinci supervised(208) | 2018 | Circuit | 3 | Moderate | 55 | 8 |
| Akinci internet(208) | 2018 | Circuit | 3 | Moderate | 35 | 8 |
| Aldred(209) | 1995 | Walk | 5 | Moderate | 35 | 12 |
| Alvarez(210) | 2019 | Walk | 3 | moderate | 80 | 20 |
| Arca water(211) | 2013 | Aqua | 3 | Moderate | 50 | 12 |
| Arca land(211) | 2013 | Bike | 3 | Moderate | 50 | 12 |
| Babu(212) | 2022 | Bike | 3 | High | 30 | 12 |
| Baker(213) | 1986 | Walk | 3 | Vigorous | 48 | 20 |
| Biddle(214) | 2011 | Walk | 3 | Vigorous | 45 | 4 |
| Binder(215) | 1996 | Walk | 4 | Vigorous | 30 | 38 |
| Bitelli normolipid(216) | 2021 | Walk | 3 | Moderate | 75 | 20 |
| Bitelli dyslipidemia(216) | 2021 | Walk | 3 | Moderate | 75 | 20 |
| Blumenthal aerobic(217) | 1991 | Bike | 3 | Moderate | 60 | 18 |
| Boardley combined(218) | 2006 | Walk | 3 | Moderate | 20 | 16 |
| Boardley strength(218) | 2007 | Strength | 3 | Moderate | 30 | 16 |
| Boardley aerobic(218) | 2007 | Walk | 3 | Moderate | 35 | 16 |
| Bobeuf(219) | 2011 | Strength | 3 | Vigorous | 60 | 26 |
| Bock(220) | 2019 | Walk/Bike | 3 | Moderate | 30 | 12 |
| Bonfante(221) | 2017 | Walk/Strength | 3 | Vigorous | 60 | 24 |
| Bonfante(222) | 2022 | Walk/Strength | 3 | Moderate | 75 | 16 |
| Boreham(223) | 2000 | Stairclimb | 5 | Vigorous | 20 | 7 |
| Boyden(224) | 1993 | Strength | 3 | Vigorous | 30 | 22 |
| Busby(225) | 1985 | Strength | 3 | Vigorous | 50 | 12 |
| Cho low(226) | 2011 | Walk | 3 | Moderate | 60 | 12 |
| Cho high(226) | 2011 | Walk | 3 | Moderate | 60 | 12 |
| Church 4(227) | 2007 | Walk | 3.5 | Moderate | 30 | 26 |
| Church 8(227) | 2007 | Walk | 3.5 | Moderate | 30 | 26 |
| Church 12(227) | 2007 | Walk | 3.5 | Moderate | 30 | 26 |
| Colado aquatic(228) | 2009 | Strength | 3 | Moderate | 30 | 24 |
| Colado elastic(228) | 2009 | Strength | 3 | Moderate | 30 | 24 |
| Connolly(229) | 2020 | Walk | 3 | Vigorous | 30 | 12 |
| Costa aquatic(230) | 2018 | Aqua | 2 | Vigorous | 45 | 12 |
| Cunningham(231) | 1987 | Walk | 3 | Vigorous | 55 | 52 |
| Dalleck 30min(232) | 2009 | Walk | 5 | Moderate | 30 | 12 |
| Dalleck 45 min(232) | 2009 | Walk | 5 | Moderate | 45 | 12 |
| Dardashti(233) | 2021 | Walk | 3 | Vigorous | 45 | 26 |
| Duncan strollers(234) | 1991 | Walk | 5 | Moderate | 60 | 24 |
| Duncan brisk(234) | 1991 | Walk | 5 | High | 36 | 24 |
| Duncan aerobic(234) | 1991 | Walk | 5 | Vigorous | 45 | 24 |
| Eguchi interval(235) | 2012 | Bike | 3 | Vigorous | 30 | 12 |
| Eguchi continuous(235) | 2012 | Bike | 3 | Vigorous | 30 | 12 |
| Elliott(236) | 2002 | Strength | 3 | Vigorous | 45 | 8 |
| Fahlman(237) | 2002 | Walk | 3 | Vigorous | 50 | 10 |
| Fahlman(237) | 2002 | Strength | 3 | Vigorous | 50 | 10 |
| Fang(238) | 2018 | Walk/Bike | 3 | Moderate | 60 | 12 |
| Farinha mod(239) | 2022 | Walk | 2 | Moderate | 45 | 28 |
| Farinha high(239) | 2022 | Walk | 2 | Vigorous | 45 | 28 |
| Farinha combined(239) | 2022 | Walk/Strength | 2 | Moderate | 45 | 28 |
| Fenkci aerobic(240) | 2006 | Walk | 3 | Vigorous | 45 | 12 |
| Fenkci resistance(240) | 2006 | Strength | 3 | Vigorous | 45 | 12 |
| Finucane(241) | 2010 | Bike | 3 | Vigorous | 60 | 12 |
| Foulds 10minx1(242) | 2014 | Walk | 1 | Vigorous | 10 | 13 |
| Foulds 10minx3(242) | 2014 | Walk | 3 | Vigorous | 10 | 13 |
| Foulds 30minx3(242) | 2014 | Run | 3 | High | 30 | 13 |
| Foulds 60minx3(242) | 2014 | Walk | 3 | Vigorous | 60 | 13 |
| Foulds 30min run x3(242) | 2014 | Run | 3 | High | 30 | 13 |
| Frank(243) | 2005 | Walk/Bike | 5 | Vigorous | 45 | 52 |
| Gram nordic(244) | 2010 | Walk | 1.5 | Moderate | 45 | 17 |
| Gram combined(244) | 2010 | Walk | 1.5 | Moderate | 45 | 17 |
| Gram bike(244) | 2018 | Bike | 5 | Moderate | 35 | 26 |
| Gram moderate(245) | 2018 | Walk | 5 | Moderate | 35 | 26 |
| Gram vigorous(245) | 2018 | Walk | 5 | Vigorous | 35 | 26 |
| Grandjean(246) | 1996 | Walk/Bike | 3 | Vigorous | 35 | 24 |
| Ha(247) | 2018 | Aqua | 3 | Moderate | 50 | 12 |
| Hagan males(248) | 1986 | Walk | 5 | Moderate | 30 | 12 |
| Hagan females(248) | 1986 | Walk | 5 | Moderate | 30 | 12 |
| Hagerman rt(249) | 2000 | Strength | 3 | High | 45 | 16 |
| Hallsworth(250) | 2015 | Bike | 3 | High | 35 | 12 |
| Hellnius(251) | 1993 | Walk | 3 | Vigorous | 37.5 | 26 |
| Hersey aerobic(252) | 1994 | Walk | 3 | Vigorous | 40 | 26 |
| Hersey resistance(252) | 1994 | Walk/Strength | 3 | Vigorous | 40 | 26 |
| Herzig(253) | 2014 | Walk | 3 | Moderate | 60 | 13 |
| Hinkleman(254) | 1993 | Walk | 5 | Vigorous | 45 | 15 |
| Ho aerobic(255) | 2012 | Walk | 5 | Moderate | 30 | 12 |
| Ho resistance(255) | 2012 | Walk/Strength | 5 | Moderate | 30 | 12 |
| Ho combined(255) | 2012 | Walk/Strength | 5 | Moderate | 30 | 12 |
| Hornstrup(256) | 2018 | Walk/Strength | 2 | High | 61 | 12 |
| Houghton(257) | 2017 | Walk/Strength | 3 | High | 52.5 | 12 |
| Hsieh(258) | 2018 | Walk/Strength | 3 | Moderate | 60 | 12 |
| Hurley(259) | 1998 | Walk/Strength | 3 | High | 60 | 16 |
| Huttunen(260) | 1979 | Walk/Strength | 2 | Moderate | 55 | 16 |
| Isler(261) | 2001 | Aerobic | 3 | Vigorous | 45 | 8 |
| Jennings(262) | 2015 | Walk | 3 | Moderate | 80 | 24 |
| Juneau men(263) | 1987 | Walk | 3 | Moderate | 47 | 24 |
| Juneau women(263) | 1987 | Walk | 3 | Moderate | 54 | 24 |
| Kantyka(264) | 2015 | Aqua | 3 | Vigorous | 45 | 14 |
| Kiens(265) | 1980 | Walk | 4 | Vigorous | 45 | 12 |
| Kim(266) | 2017 | Aqua | 3 | High | 50 | 12 |
| Knight(267) | 2015 | Walk/Strength | 5 | Vigorous | 30 | 12 |
| Kraus low/mod(268) | 2002 | Walk | 3 | Vigorous | 75 | 38 |
| Kraus low/high/int(268) | 2002 | Jog | 3 | Vigorous | 30 | 38 |
| Kraus high/high(268) | 2002 | Jog | 3 | vigorous | 40 | 38 |
| Krustrup soccer(269) | 2009 | Run | 3 | Vigorous | 60 | 12 |
| Krustrup running(269) | 2009 | Run | 3 | High | 60 | 12 |
| Kukkonen-harjula(270) | 1998 | Walk | 4 | Vigorous | 50 | 15 |
| Laaksonen(271) | 2000 | Run | 4 | Vigorous | 25 | 14 |
| Lamina(272) | 2013 | Bike | 3 | Vigorous | 52.5 | 8 |
| Lemura aerobic(273) | 2000 | Walk/Bike | 3 | Moderate | 50 | 16 |
| Lemura resistance(273) | 2000 | Strength | 3 | Moderate | 50 | 16 |
| Lemura x-training(273) | 2000 | Walk/Strength | 3 | Moderate | 50 | 16 |
| Leon(274) | 1996 | Walk | 5 | Vigorous | 52 | 12 |
| Liao(275) | 2015 | Walk | 5 | Moderate | 30 | 12 |
| Libardi resistance(276) | 2012 | Strength | 3 | Moderate | 60 | 16 |
| Libardi aerobic(276) | 2012 | Walk/Strength | 3 | Moderate | 60 | 16 |
| Libardi combined(276) | 2012 | Walk | 3 | Moderate | 60 | 16 |
| Magahles moderate(277) | 2020 | Walk/Strength | 3 | Vigorous | 25 | 52 |
| Magahles hiit(277) | 2020 | Walk/Strength | 3 | Moderate | 25 | 52 |
| Manning(278) | 1991 | Strength | 3 | Moderate | 40 | 12 |
| Mashnafi(279) | 2021 | Bike | 3 | Moderate | 30 | 8 |
| Mendham(280) | 2015 | Walk/Strength | 2 | Vigorous | 45 | 12 |
| Miyaki(281) | 2012 | Walk/Bike | 4 | Vigorous | 40 | 8 |
| Mohr(282) | 2014 | Run | 3 | High | 60 | 15 |
| Morey(283) | 2012 | Walk | 5 | Moderate | 30 | 38 |
| Morgan(284) | 2010 | walk | 7 | Low | 70 | 43 |
| Motoyama(285) | 1995 | walk | 5 | Moderate | 30 | 39 |
| Murphy(286) | 2006 | walk | 2 | Moderate | 45 | 8 |
| Murtagh single bout(287) | 2005 | walk | 3 | Moderate | 20 | 12 |
| Murtagh accumulated(287) | 2005 | walk | 3 | Moderate | 20 | 12 |
| Musa(288) | 2009 | run | 3 | High | 20 | 8 |
| Neves(289) | 2017 | walk/str | 3 | Moderate | 56 | 16 |
| Niederseer(290) | 2011 | skiing | 2 | Vigorous | 210 | 12 |
| Nielsen(291) | 2022 | circuit | 1 | Vigorous | 90 | 15 |
| Nieman(292) | 1993 | walk | 5 | Moderate | 35 | 12 |
| Nieman(293) | 2002 | walk | 5 | Moderate | 45 | 12 |
| Nualnim(294) | 2012 | swim | 3 | Moderate | 40 | 12 |
| Nuttamonwarakul(295) | 2012 | aqua | 3 | Moderate | 30 | 12 |
| Nybo interval(296) | 2010 | run | 2 | High | 10 | 12 |
| Nybo prolonged(296) | 2010 | run | 3 | Vigorous | 60 | 12 |
| Nybo strength(296) | 2010 | strength | 2 | Vigorous | 60 | 12 |
| O’donovan(297) | 2005 | Bike | 3 | Moderate | 60 | 24 |
| O'donovan(297) | 2005 | Bike | 3 | Vigorous | 50 | 24 |
| Ohta(298) | 2015 | Step | 7 | Moderate | 45 | 16 |
| Park aerobic(299) | 2003 | Walk | 6 | Moderate | 60 | 24 |
| Park combined(299) | 2003 | Combined | 6 | Moderate | 60 | 24 |
| Park(300) | 2015 | Circuit | 3 | Moderate | 60 | 12 |
| Patterson(301) | 2017 | Badminton | 3 | Vigorous | 60 | 8 |
| Patterson(301) | 2017 | Running | 3 | Vigorous | 60 | 8 |
| Pereira(302) | 2020 | Handball | 2 | Vigorous | 60 | 16 |
| Prabhakaran(303) | 1999 | Strength | 3 | Vigorous | 45 | 14 |
| Rahimi (304) | 2013 | Aqua | 3 | Moderate | 25 | 6 |
| Rahami (304) | 2013 | Walking | 3 | Moderate | 25 | 6 |
| Ranasinghe(305) | 2021 | Walking | 2 | Vigorous | 75 | 12 |
| Ranasinghe(305) | 2021 | Strength | 2 | Vigorous | 75 | 12 |
| Raz(306) | 1998 | Run | 2 | Vigorous | 45 | 9 |
| Ready 3 times(307) | 1996 | Walk | 3 | Moderate | 60 | 24 |
| Ready 5 times(307) | 1996 | Walk | 5 | Moderate | 60 | 24 |
| Rowland(308) | 1998 | Bike | 5 | Moderate | 30 | 12 |
| Santiago(309) | 1995 | Walk | 4 | Moderate | 53 | 40 |
| Sbardelotto(310) | 2019 | Aerobic | 3 | Vigorous | 60 | 8 |
| Sbardelotto(310) | 2019 | Combined | 3 | Vigorous | 60 | 8 |
| Sbardelotto(310) | 2019 | Combined | 3 | Vigorous | 60 | 8 |
| Schroeder(311) | 2018 | Walk/Bike | 3 | Vigorous | 60 | 8 |
| Schroeder(311) | 2018 | Strength | 3 | Vigorous | 60 | 8 |
| Schroeder(311) | 2018 | Combined | 3 | Vigorous | 60 | 8 |
| Schuit(312) | 1998 | Bike | 4 | Moderate | 45 | 26 |
| Schuit(312) | 1998 | Combined | 4 | Moderate | 30 | 26 |
| Shaw(313) | 2009 | Bike | 3 | Vigorous | 45 | 16 |
| Shaw(313) | 2009 | Combined | 3 | Vigorous | 45 | 16 |
| Sian(314) | 2022 | Circuit | 3 | High | 5 | 4 |
| Sian(314) | 2022 | Circuit | 3 | High | 5 | 4 |
| Sillinpaa(315) | 2009 | Bike | 2 | Vigorous | 45 | 21 |
| Sillinpaa(315) | 2009 | Strength | 2 | Vigorous | 75 | 21 |
| Sillinpaa(315) | 2009 | Combined | 2 | Vigorous | 120 | 21 |
| Smith-Ryan 2min hitt(316) | 2015 | Bike | 3 | High | 10 | 3 |
| Smith-Ryan 1 min hitt(316) | 2015 | Bike | 3 | High | 10 | 3 |
| Smutok resistance(317) | 1993 | Strength | 3 | Vigorous | 30 | 20 |
| Smutok aerobic(317) | 1993 | Walking | 3 | Vigorous | 30 | 20 |
| So(318) | 2013 | Strength | 3 | Moderate | 60 | 12 |
| Sousa aerobic(319) | 2014 | Walk | 3 | Vigorous | 60 | 32 |
| Sousa combined(319) | 2014 | Combined | 3 | Vigorous | 60 | 32 |
| Steffanick(320) | 1998 | Walking | 3 | Moderate | 60 | 52 |
| Stein moderate(321) | 1990 | Bike | 3 | Moderate | 24 | 12 |
| Stein vigorous(321) | 1990 | Bike | 3 | Vigorous | 24 | 12 |
| Stein high(321) | 1990 | Bike | 3 | High | 24 | 12 |
| Stensel(322) | 1993 | Walking | 2 | Moderate | 28 | 52 |
| Stensvold hiit(323) | 2020 | Walking | 3 | High | 16 | 12 |
| Stensvold strength(323) | 2020 | Strength | 3 | High | 43 | 12 |
| Stensvold combined(323) | 2020 | Combined | 3 | High | 25 | 12 |
| Stewart(324) | 2005 | Combined | 3 | Vigorous | 45 | 26 |
| Sunami(325) | 1999 | Walking | 3 | Low | 60 | 24 |
| Suter(326) | 1992 | Walking | 4 | Moderate | 30 | 17 |
| Suter jogging(327) | 1994 | Run | 4 | Vigorous | 30 | 26 |
| Suter walking(327) | 1994 | Walking | 6 | Moderate | 30 | 26 |
| Swift aerobic(328) | 2021 | Walking | 3 | Vigorous | 47 | 24 |
| Swift steps(328) | 2021 | Walking | 7 | Moderate | 45 | 24 |
| Takeshima(329) | 2002 | Aqua | 3 | Moderate | 70 | 12 |
| Takeshima(330) | 2004 | Combined | 3 | Moderate | 50 | 12 |
| Taniguchi(331) | 2015 | Bike | 3 | Vigorous | 39 | 5 |
| Thomas 4-m(332) | 1984 | Run | 3 | Vigorous | 31 | 12 |
| Thomas 4-f(332) | 1984 | Run | 3 | Vigorous | 31 | 12 |
| Thomas 2-m(332) | 1984 | Run | 3 | Vigorous | 31 | 12 |
| Thomas 2-f(332) | 1984 | Run | 3 | Vigorous | 31 | 12 |
| Thomas i-m(332) | 1984 | Run | 3 | Vigorous | 32 | 12 |
| Thomas i-f(332) | 1984 | Run | 3 | Vigorous | 32 | 12 |
| Thompson(333) | 2010 | Bike | 4 | Moderate | 45 | 24 |
| Tjonna mod(334) | 2008 | Walking | 3 | Moderate | 47 | 16 |
| Tjonna high(334) | 2008 | Walking | 3 | High | 40 | 16 |
| Tokudome(335) | 2004 | Strength | 3 | Vigorous | 45 | 12 |
| Trajkovic(336) | 2020 | Run | 3 | Vigorous | 90 | 10 |
| Verissimo(337) | 2002 | Combined | 3 | Vigorous | 60 | 32 |
| Vesterbekkmo(338) | 2022 | Combined | 2 | Vigorous | 40 | 24 |
| Vincent low(339) | 2003 | Strength | 3 | Vigorous | 45 | 24 |
| Vincent high(339) | 2003 | Strength | 3 | High | 45 | 24 |
| Vincent normal wt.(340) | 2006 | Strength | 3 | High | 45 | 24 |
| Vincent overwt.(340) | 2006 | Strength | 3 | High | 45 | 24 |
| Vincente-campos(341) | 2012 | Combined | 3 | Moderate | 50 | 28 |
| Vinetti(342) | 2019 | Combined | 3 | Vigorous | 45 | 52 |
| Watkins(343) | 2003 | Combined | 3.5 | Vigorous | 26 | 26 |
| Wen(344) | 2016 | Dancing | 3 | Vigorous | 10 | 10 |
| Whitehurst(345) | 1991 | Walking | 3 | Vigorous | 8 | 8 |
| Williams low(346) | 1994 | Combined | 4 | Vigorous | 52 | 52 |
| Williams interval(346) | 1994 | Combined | 4 | Vigorous | 52 | 52 |
| Williams high(346) | 1994 | Combined | 4 | vigorous | 52 | 52 |
| Woolf-may lw(347) | 1998 | Walking | 3.5 | Vigorous | 18 | 18 |
| Woolf-may rsw(347) | 1998 | Walking | 3.5 | Vigorous | 18 | 18 |
| Wooten(348) | 2011 | Combined | 3 | Vigorous | 12 | 12 |
| Wu women(349) | 2011 | Combined | 5 | Vigorous | 36 | 13 |
| Wu men(349) | 2011 | Combined | 5 | Vigorous | 36 | 13 |
| Zelber-sagi(350) | 2015 | Strength | 3 | Vigorous | 45 | 13 |
| Zhang(351) | 2016 | Walking | 5 | Low | 30 | 52 |
| Zhang(351) | 2016 | Combined | 5 | Moderate | 30 | 52 |

**TABLE S5. STUDY QUALITY ASSESSMENT USING TESTEX SCALE**

| **Study** | **Eligibility criteria specified** | **Randomization specified** | **Allocation concealment** | **Groups similar at baseline** | **Blinding of assessor** | **Outcome measures assessed >85% of participants^#^** | **Intention to treat analysis** | **Between group statistical comparisons reported^*^** | **Point measures & measures of variability reported** | **Activity monitoring in control group** | **Relative exercise intensity review** | **Exercise volume & energy expenditure** | **Overall TESTEX** |  |
| --- | --- | --- | --- | --- | --- | --- | --- | --- | --- | --- | --- | --- | --- | --- |
| Abdelbassett 2019 (204) | 1 | 1 | 1 | 1 | 1 | 1 | 1 | 1 | 1 | 0 | 1 | 0 | 10 |  |
| Abdelbassett 2020 (205) | 1 | 1 | 1 | 1 | 1 | 1 | 0 | 1 | 1 | 0 | 0 | 0 | 8 |  |
| Afzalpour et al. 2008 (206) | 1 | 0 | 0 | 1 | 0 | 0 | 0 | 2 | 1 | 0 | 0 | 1 | 6 |  |
| Agner et al. 2018 (207) | 1 | 1 | 0 | 1 | 1 | 0 | 1 | 1 | 1 | 0 | 1 | 0 | 8 |  |
| Akinci al. 2018 (208) | 1 | 1 | 1 | 1 | 1 | 2 | 1 | 2 | 1 | 0 | 1 | 0 | 12 |  |
| Aldred 1995 (209) | 1 | 0 | 1 | 0 | 0 | 1 | 0 | 2 | 1 | 0 | 1 | 0 | 7 |  |
| Alvarez 2019 (210) | 1 | 1 | 0 | 1 | 0 | 0 | 0 | 2 | 1 | 0 | 1 | 0 | 7 |  |
| Arca 2013 (211) | 1 | 0 | 0 | 1 | 0 | 1 | 1 | 2 | 1 | 0 | 1 | 0 | 8 |  |
| Babu 2022 (212) | 1 | 1 | 0 | 1 | 0 | 1 | 0 | 2 | 1 | 0 | 1 | 1 | 9 |  |
| Baker 1986 (213) | 1 | 0 | 0 | 1 | 0 | 1 | 0 | 2 | 1 | 0 | 1 | 1 | 8 |  |
| Biddle 2011 (214) | 1 | 1 | 1 | 1 | 0 | 0 | 1 | 2 | 1 | 0 | 0 | 1 | 9 |  |
| Binder 1996 (215) | 1 | 0 | 0 | 1 | 0 | 1 | 1 | 2 | 1 | 0 | 1 | 0 | 8 |  |
| Bitelli 2021 (216) | 1 | 0 | 0 | 1 | 0 | 1 | 1 | 2 | 1 | 0 | 1 | 0 | 8 |  |
| Blumenthal 1991(217) | 1 | 0 | 0 | 1 | 0 | 1 | 1 | 2 | 1 | 0 | 1 | 1 | 9 |  |
| Boardley 2007 (218) | 1 | 0 | 0 | 0 | 0 | 1 | 0 | 1 | 1 | 0 | 1 | 0 | 5 |  |
| Bobeuf 2011 (219) | 1 | 0 | 0 | 1 | 0 | 1 | 1 | 2 | 1 | 0 | 0 | 0 | 7 |  |
| Bock 2019 (220) | 1 | 1 | 0 | 1 | 0 | 0 | 1 | 2 | 1 | 0 | 1 | 0 | 8 |  |
| Bonfante 2017 (221) | 1 | 0 | 0 | 1 | 0 | 1 | 1 | 2 | 1 | 0 | 1 | 1 | 9 |  |
| Bonfante 2022 (222) | 1 | 0 | 0 | 1 | 0 | 1 | 1 | 2 | 1 | 0 | 1 | 1 | 9 |  |
| Boreham 2000 (223) | 1 | 0 | 0 | 1 | 0 | 0 | 0 | 2 | 1 | 0 | 1 | 0 | 6 |  |
| Boyden 1993 (224) | 1 | 0 | 0 | 1 | 0 | 0 | 0 | 2 | 1 | 0 | 1 | 1 | 7 |  |
| Busby 1985 (225) | 1 | 0 | 0 | 1 | 0 | 1 | 0 | 2 | 1 | 0 | 1 | 0 | 7 |  |
| Cho 2011 (226) | 1 | 1 | 1 | 1 | 1 | 0 | 0 | 2 | 1 | 0 | 1 | 1 | 10 |  |
| Church 2007 (227) | 1 | 1 | 1 | 1 | 1 | 2 | 1 | 2 | 1 | 1 | 1 | 1 | 14 |  |
| Colado 2009 (228) | 1 | 0 | 0 | 1 | 0 | 0 | 0 | 2 | 1 | 0 | 1 | 0 | 6 |  |
| Connolly 2020 (229) | 1 | 1 | 1 | 1 | 0 | 0 | 0 | 2 | 1 | 0 | 0 | 0 | 7 |  |
| Costa 2018 (230) | 1 | 1 | 1 | 1 | 0 | 0 | 0 | 2 | 1 | 0 | 0 | 0 | 7 |  |
| Cunningham 1987 (231) | 1 | 0 | 0 | 1 | 0 | 1 | 0 | 2 | 1 | 0 | 1 | 1 | 8 |  |
| Dalleck 2009 (232) | 1 | 0 | 0 | 1 | 0 | 1 | 0 | 2 | 1 | 0 | 1 | 1 | 8 |  |
| Dardashti 2021 (233) | 1 | 0 | 0 | 1 | 1 | 0 | 0 | 2 | 1 | 0 | 1 | 1 | 8 |  |
| Duncan 1991 (234) | 1 | 0 | 0 | 1 | 0 | 0 | 0 | 2 | 1 | 0 | 1 | 1 | 7 |  |
| Eguchi 2012 (235) | 1 | 0 | 0 | 1 | 0 | 1 | 0 | 2 | 1 | 0 | 1 | 1 | 8 |  |
| Elliott 2002 (236) | 1 | 0 | 0 | 1 | 0 | 1 | 1 | 2 | 1 | 0 | 1 | 0 | 8 |  |
| Fahlman 2002 (237) | 0 | 1 | 0 | 0 | 0 | 1 | 0 | 1 | 1 | 0 | 1 | 1 | 6 |  |
| Fang 2018 (238) | 1 | 0 | 0 | 1 | 0 | 0 | 0 | 2 | 1 | 0 | 0 | 0 | 5 |  |
| Farinha 2022 (239) | 1 | 0 | 0 | 1 | 1 | 1 | 0 | 1 | 1 | 0 | 1 | 0 | 7 |  |
| Fencki 2006 (240) | 1 | 0 | 0 | 1 | 0 | 1 | 0 | 1 | 1 | 0 | 1 | 0 | 6 |  |
| Finucane 2010 (241) | 1 | 1 | 0 | 1 | 0 | 2 | 1 | 1 | 1 | 0 | 1 | 0 | 9 |  |
| Foulds 2014 (242) | 1 | 0 | 0 | 1 | 0 | 1 | 0 | 1 | 1 | 0 | 0 | 0 | 5 |  |
| Frank 2005 (243) | 1 | 1 | 0 | 1 | 1 | 2 | 1 | 1 | 1 | 1 | 1 | 1 | 12 |  |
| Gram 2010 (244) | 1 | 1 | 0 | 1 | 0 | 1 | 1 | 1 | 1 | 0 | 0 | 1 | 8 |  |
| Gram 2018 (245) | 1 | 0 | 0 | 1 | 0 | 0 | 0 | 2 | 1 | 0 | 1 | 1 | 7 |  |
| Grandjean 1996 (246) | 1 | 0 | 0 | 1 | 0 | 0 | 0 | 2 | 1 | 0 | 0 | 1 | 6 |  |
| Ha 2017 (247) | 1 | 0 | 0 | 1 | 0 | 1 | 1 | 2 | 1 | 0 | 0 | 0 | 7 |  |
| Hagan 1986 (248) | 1 | 0 | 0 | 1 | 0 | 0 | 0 | 1 | 1 | 0 | 0 | 1 | 5 |  |
| Hagerman 2000 (249) | 1 | 0 | 0 | 1 | 0 | 0 | 0 | 2 | 1 | 0 | 0 | 1 | 6 |  |
| Hallsworth, 2015 (250) | 1 | 1 | 0 | 1 | 0 | 0 | 0 | 1 | 1 | 0 | 0 | 0 | 5 |  |
| Hellnius 1993 (251) | 1 | 0 | 0 | 1 | 0 | 1 | 0 | 2 | 1 | 0 | 0 | 0 | 6 |  |
| Hersey 1994 (252) | 1 | 0 | 0 | 1 | 0 | 0 | 0 | 1 | 1 | 0 | 0 | 1 | 5 | |
| Herzig 2014 (253) | 1 | 1 | 0 | 0 | 0 | 1 | 0 | 2 | 1 | 0 | 0 | 1 | 7 | |
| Hinkleman 1993 (254) | 1 | 0 | 0 | 1 | 0 | 0 | 0 | 2 | 1 | 1 | 0 | 0 | 6 | |
| Ho 2012 (255) | 1 | 1 | 1 | 1 | 0 | 0 | 0 | 1 | 1 | 0 | 1 | 1 | 8 | |
| Hornstrup 2019 (256) | 1 | 0 | 0 | 1 | 0 | 0 | 0 | 2 | 1 | 0 | 0 | 1 | 6 | |
| Houghton 2017 (257) | 1 | 1 | 0 | 1 | 0 | 1 | 0 | 1 | 1 | 0 | 1 | 0 | 7 | |
| Hsieh 2018 (258) | 1 | 1 | 0 | 1 | 1 | 1 | 1 | 2 | 1 | 0 | 1 | 1 | 11 | |
| Hurley 1988 (259) | 0 | 0 | 0 | 1 | 0 | 1 | 0 | 1 | 1 | 0 | 1 | 1 | 6 | |
| Huttunen 1979 (260) | 0 | 0 | 0 | 1 | 0 | 1 | 0 | 2 | 1 | 0 | 0 | 1 | 6 | |
| Isler 2001 (261) | 1 | 0 | 0 | 1 | 0 | 0 | 0 | 1 | 1 | 0 | 1 | 0 | 5 | |
| Jennings 2015 (262) | 1 | 1 | 0 | 1 | 1 | 1 | 1 | 2 | 1 | 0 | 0 | 0 | 9 | |
| Juneau 1987 (263) | 1 | 0 | 0 | 1 | 0 | 1 | 0 | 1 | 1 | 0 | 0 | 1 | 6 | |
| Kantyka 2015 (264) | 1 | 0 | 0 | 1 | 0 | 0 | 0 | 1 | 1 | 0 | 1 | 0 | 5 | |
| Kiens 1980 (265) | 1 | 0 | 0 | 1 | 0 | 0 | 0 | 1 | 1 | 0 | 0 | 1 | 5 | |
| Kim 2017 (266) | 1 | 0 | 0 | 1 | 0 | 0 | 0 | 1 | 1 | 0 | 1 | 0 | 5 | |
| Knight 2014 (267) | 0 | 1 | 0 | 1 | 0 | 1 | 0 | 1 | 1 | 0 | 0 | 1 | 6 | |
| Kraus 2002 (268) | 1 | 0 | 0 | 1 | 0 | 0 | 1 | 1 | 1 | 0 | 0 | 0 | 5 | |
| Krustrup 2009 (269) | 1 | 0 | 0 | 1 | 0 | 1 | 0 | 1 | 1 | 0 | 0 | 1 | 6 | |
| Kukkone-Harjule 1998 (270) | 1 | 0 | 0 | 1 | 0 | 1 | 1 | 2 | 1 | 0 | 0 | 1 | 8 | |
| Laaksonen 2000 (271) | 1 | 0 | 0 | 1 | 0 | 0 | 0 | 1 | 1 | 0 | 0 | 1 | 5 | |
| Lamina 2013 (272) | 1 | 0 | 1 | 1 | 0 | 0 | 0 | 1 | 1 | 0 | 0 | 1 | 6 | |
| LeMura 2000 (273) | 1 | 0 | 0 | 1 | 0 | 1 | 0 | 1 | 1 | 1 | 1 | 1 | 8 | |
| Leon 1996 (274) | 1 | 0 | 0 | 1 | 0 | 0 | 0 | 1 | 1 | 1 | 0 | 1 | 5 | |
| Liao 2015 (275) | 1 | 0 | 0 | 1 | 0 | 1 | 0 | 1 | 1 | 0 | 0 | 0 | 5 | |
| Libardi 2012 (276) | 1 | 0 | 0 | 1 | 0 | 1 | 1 | 2 | 1 | 0 | 0 | 0 | 7 |  |
| Magalhaes 2020 (277) | 1 | 1 | 0 | 1 | 1 | 2 | 1 | 2 | 1 | 0 | 1 | 1 | 12 |  |
| Manning 1991 (278) | 1 | 0 | 0 | 1 | 0 | 1 | 0 | 2 | 1 | 0 | 1 | 0 | 7 |  |
| Mashnafi 2021 (279) | 1 | 1 | 0 | 0 | 0 | 1 | 0 | 1 | 1 | 0 | 1 | 1 | 7 |  |
| Mendham 2015 (280) | 1 | 1 | 0 | 0 | 0 | 1 | 0 | 2 | 1 | 0 | 1 | 1 | 8 |  |
| Miyaki 2012 (281) | 1 | 0 | 0 | 1 | 0 | 2 | 0 | 0 | 1 | 0 | 1 | 1 | 7 |  |
| Mohr 2014 (282) | 1 | 0 | 0 | 1 | 0 | 3 | 0 | 2 | 0 | 0 | 0 | 1 | 8 |  |
| Morey 2012 (283) | 1 | 1 | 0 | 0 | 0 | 3 | 1 | 2 | 1 | 0 | 0 | 0 | 9 |  |
| Morgan 2010 (284) | 1 | 0 | 0 | 0 | 0 | 0 | 1 | 0 | 2 | 0 | 0 | 0 | 4 |  |
| Motoyama 1995 (285) | 1 | 0 | 0 | 1 | 0 | 0 | 0 | 2 | 1 | 0 | 1 | 1 | 7 |  |
| Murphy 2006 (286) | 1 | 0 | 0 | 1 | 0 | 2 | 0 | 2 | 1 | 1 | 1 | 1 | 10 |  |
| Murtagh 2005 (287) | 1 | 0 | 0 | 0 | 0 | 2 | 0 | 0 | 1 | 0 | 0 | 1 | 5 |  |
| Musa 2009 (288) | 1 | 0 | 0 | 1 | 0 | 1 | 0 | 2 | 0 | 0 | 0 | 1 | 6 |  |
| Neves 2017 (289) | 1 | 0 | 1 | 1 | 0 | 1 | 0 | 2 | 1 | 0 | 1 | 0 | 8 |  |
| Niederseer 2011 (290) | 1 | 0 | 0 | 1 | 0 | 2 | 0 | 2 | 1 | 0 | 0 | 0 | 7 |  |
| Nielsen 2022 (291) | 1 | 1 | 0 | 0 | 0 | 2 | 1 | 2 | 0 | 0 | 0 | 0 | 7 |  |
| Nieman 1993 (292) | 1 | 0 | 1 | 0 | 0 | 3 | 0 | 2 | 1 | 1 | 1 | 1 | 11 |  |
| Nieman 2002 (293) | 1 | 0 | 0 | 1 | 0 | 2 | 0 | 2 | 1 | 0 | 1 | 1 | 9 |  |
| Nualnim 2012 (294) | 1 | 0 | 0 | 1 | 0 | 1 | 0 | 0 | 1 | 0 | 1 | 1 | 6 |  |
| Nuttamonwarakul 2012 (295) | 0 | 0 | 0 | 1 | 0 | 0 | 0 | 2 | 1 | 0 | 0 | 1 | 5 |  |
| Nybo 2010 (296) | 1 | 0 | 0 | 0 | 0 | 2 | 0 | 0 | 1 | 0 | 0 | 1 | 5 |  |
| O’Donovan 2005 (297) | 1 | 1 | 0 | 1 | 1 | 1 | 0 | 2 | 1 | 0 | 1 | 1 | 10 |  |
| Ohta 2015 (298) | 0 | 0 | 0 | 1 | 0 | 1 | 1 | 2 | 1 | 0 | 0 | 1 | 7 |  |
| Park 2003 (299) | 0 | 0 | 0 | 1 | 0 | 0 | 0 | 2 | 1 | 0 | 0 | 1 | 5 |  |
| Park 2015 (300) | 1 | 1 | 0 | 0 | 1 | 2 | 0 | 2 | 1 | 0 | 1 | 0 | 9 |  |
| Patterson 2016 (301) | 1 | 0 | 0 | 0 | 0 | 3 | 0 | 0 | 1 | 0 | 0 | 0 | 5 |  |
| Pereira 2020 (302) | 0 | 0 | 0 | 0 | 0 | 2 | 0 | 2 | 1 | 0 | 0 | 1 | 6 |  |
| Prabhakaran 1999 (303) | 1 | 0 | 0 | 1 | 0 | 1 | 0 | 2 | 1 | 0 | 1 | 0 | 7 |  |
| Rahimi 2013 (304) | 1 | 0 | 0 | 0 | 0 | 0 | 0 | 0 | 1 | 0 | 1 | 1 | 4 |  |
| Ranasinghe 2021 (305) | 1 | 1 | 0 | 1 | 1 | 1 | 0 | 2 | 1 | 1 | 0 | 1 | 10 |  |
| Raz 1998 (306) | 1 | 0 | 0 | 0 | 0 | 2 | 0 | 2 | 1 | 1 | 1 | 1 | 9 |  |
| Ready 1996 (307) | 1 | 0 | 0 | 1 | 0 | 2 | 0 | 0 | 1 | 0 | 1 | 1 | 7 |  |
| Rowland 1998 (308) | 1 | 1 | 0 | 1 | 0 | 1 | 1 | 0 | 1 | 0 | 0 | 0 | 6 |  |
| Santiago 1995 (309) | 1 | 1 | 0 | 0 | 0 | 1 | 0 | 1 | 1 | 0 | 1 | 1 | 7 |  |
| Sbardelotto 2019 (310) | 1 | 1 | 1 | 0 | 0 | 0 | 0 | 2 | 1 | 0 | 1 | 1 | 8 |  |
| Schroeder 2018 (311) | 1 | 1 | 1 | 0 | 1 | 3 | 0 | 2 | 1 | 0 | 1 | 1 | 12 |  |
| Schuit 1998 (312) | 1 | 0 | 1 | 0 | 0 | 2 | 0 | 2 | 1 | 0 | 0 | 0 | 7 |  |
| Shaw 2009 (313) | 1 | 0 | 0 | 0 | 0 | 1 | 0 | 2 | 1 | 0 | 1 | 1 | 7 |  |
| Sian 2022 (314) | 1 | 1 | 1 | 1 | 0 | 3 | 0 | 2 | 1 | 0 | 1 | 1 | 12 |  |
| Sillanpaa 2009 (315) | 1 | 0 | 1 | 1 | 0 | 1 | 0 | 2 | 1 | 0 | 0 | 1 | 8 |  |
| Smith-Ryan 2015 (316) | 1 | 0 | 1 | 0 | 0 | 2 | 0 | 1 | 1 | 0 | 1 | 1 | 8 |  |
| Smutok 1993 (317) | 1 | 0 | 0 | 1 | 0 | 1 | 0 | 2 | 1 | 0 | 0 | 1 | 7 |  |
| So 2013 (318) | 1 | 0 | 0 | 0 | 1 | 2 | 0 | 1 | 1 | 0 | 0 | 0 | 6 |  |
| Sousa 2014 (319) | 1 | 0 | 1 | 1 | 0 | 2 | 0 | 2 | 1 | 0 | 1 | 0 | 9 |  |
| Steffanick 1998 (320) | 1 | 1 | 1 | 1 | 0 | 1 | 1 | 2 | 1 | 0 | 0 | 0 | 9 |  |
| Stein 1990 (321) | 1 | 0 | 0 | 1 | 0 | 2 | 0 | 1 | 1 | 0 | 0 | 0 | 6 |  |
| Stensel 1993 (322) | 1 | 0 | 0 | 0 | 0 | 2 | 0 | 1 | 1 | 0 | 1 | 1 | 7 |  |
| Stensvold 2010 (323) | 1 | 1 | 1 | 0 | 0 | 2 | 0 | 1 | 1 | 0 | 1 | 1 | 9 |  |
| Stewart 2005 (324) | 1 | 0 | 0 | 1 | 0 | 1 | 0 | 1 | 1 | 0 | 1 | 1 | 7 |  |
| Sunami 1999 (325) | 1 | 1 | 0 | 1 | 0 | 2 | 0 | 1 | 1 | 0 | 1 | 0 | 8 |  |
| Suter 1992 (326) | 1 | 0 | 0 | 1 | 0 | 1 | 1 | 1 | 0 | 0 | 0 | 0 | 5 |  |
| Suter 1994 (327) | 1 | 0 | 0 | 1 | 0 | 2 | 1 | 1 | 1 | 0 | 0 | 1 | 8 |  |
| Swift 2021 (328) | 1 | 0 | 1 | 1 | 1 | 3 | 0 | 2 | 1 | 0 | 1 | 1 | 12 |  |
| Takeshima 2002 (329) | 1 | 0 | 0 | 1 | 0 | 3 | 1 | 2 | 1 | 0 | 1 | 1 | 11 |  |
| Takeshima 2004 (330) | 1 | 0 | 0 | 1 | 0 | 3 | 1 | 2 | 1 | 0 | 1 | 1 | 11 |  |
| Taniguchi 2015 (331) | 1 | 0 | 0 | 1 | 0 | 2 | 1 | 2 | 1 | 0 | 1 | 1 | 10 |  |
| Thomas 1984 (332) | 1 | 0 | 0 | 1 | 0 | 0 | 0 | 0 | 1 | 0 | 1 | 1 | 5 |  |
| Thompson 2010 (333) | 1 | 1 | 0 | 1 | 0 | 0 | 0 | 2 | 1 | 0 | 1 | 1 | 8 |  |
| Tjonna 2008 (334) | 1 | 0 | 0 | 1 | 0 | 3 | 0 | 2 | 1 | 0 | 1 | 1 | 10 |  |
| Tokudome 2004 (335) | 1 | 0 | 0 | 1 | 0 | 3 | 0 | 0 | 1 | 1 | 1 | 0 | 8 |  |
| Trajkovic 2020 (336) | 1 | 0 | 0 | 1 | 0 | 3 | 1 | 2 | 1 | 0 | 0 | 1 | 10 |  |
| Verissimo 2002 (337) | 1 | 0 | 0 | 1 | 0 | 2 | 0 | 0 | 1 | 0 | 1 | 0 | 6 |  |
| Vesterbekkmo 2022 (338) | 1 | 1 | 0 | 1 | 0 | 0 | 0 | 2 | 1 | 0 | 1 | 1 | 8 |  |
| Vincent 2003 (339) | 1 | 1 | 0 | 1 | 0 | 1 | 1 | 2 | 1 | 0 | 1 | 1 | 10 |  |
| Vincent 2007 (340) | 1 | 0 | 0 | 1 | 0 | 3 | 1 | 0 | 1 | 0 | 1 | 1 | 9 |  |
| Vincente-Campos 2012 (341) | 1 | 0 | 0 | 1 | 0 | 0 | 0 | 0 | 1 | 0 | 1 | 1 | 5 |  |
| Vinetti 2019 (342) | 1 | 0 | 0 | 1 | 0 | 1 | 1 | 2 | 1 | 0 | 1 | 1 | 9 |  |
| Watkins 2003 (343) | 1 | 0 | 0 | 1 | 0 | 0 | 0 | 2 | 1 | 0 | 1 | 1 | 7 |  |
| Wen 2016 (344) | 1 | 0 | 0 | 1 | 0 | 3 | 0 | 2 | 1 | 0 | 1 | 1 | 10 |  |
| Whitehurst 1991 (345) | 1 | 0 | 0 | 1 | 0 | 1 | 0 | 2 | 1 | 0 | 1 | 1 | 8 |  |
| Williams 1994 (346) | 1 | 0 | 0 | 0 | 0 | 1 | 1 | 2 | 1 | 0 | 1 | 1 | 8 |  |
| Woolf-May 1998 (347) | 1 | 0 | 0 | 1 | 1 | 0 | 0 | 0 | 1 | 0 | 1 | 1 | 6 |  |
| Wooten 2011 (348) | 1 | 0 | 0 | 1 | 0 | 1 | 0 | 0 | 1 | 0 | 1 | 0 | 5 |  |
| Wu 2011 (349) | 1 | 1 | 0 | 1 | 0 | 3 | 1 | 2 | 1 | 0 | 0 | 0 | 10 |  |
| Zelber-Sagi 2021 (350) | 1 | 0 | 0 | 1 | 1 | 2 | 1 | 0 | 1 | 0 | 1 | 0 | 8 |  |
| Zhang 2016 (351) | 1 | 1 | 0 | 1 | 0 | 3 | 0 | 2 | 1 | 0 | 1 | 1 | 11 |  |
| **Median Score** | | | | | | | | | | | | |  |  |

Total out of 15 Points

# Three points possible: 1 point – if adherence >85%, 1 point – if adverse events reported, 1 point – if exercise attendance is reported

* Two points possible: 1 point – if between-group statistical comparisons are reported for the primary outcome measure of interest, 1 point – if between-group statistical comparisons are reported for at least one secondary outcome measure

**REFERENCES**

1. Aekplakorn W, Tantayotai V, Numsangkul S, Tatsato N, Luckanajantachote P, Himathongkam T. Evaluation of a Community-Based Diabetes Prevention Program in Thailand: A Cluster Randomized Controlled Trial. J Prim Care Community Health. 2019;10:2150132719847374.

2. Akkurt H, Karapolat HU, Kirazli Y, Kose T. The effects of upper extremity aerobic exercise in patients with spinal cord injury: a randomized controlled study. Eur J Phys Rehabil Med. 2017;53(2):219-27.

3. Aller R, Izaola O, Gomez S, Tafur C, Gonzalez G, Berroa E, et al. Effect of silymarin plus vitamin E in patients with non-alcoholic fatty liver disease. A randomized clinical pilot study. Eur Rev Med Pharmacol Sci. 2015;19(16):3118-24.

4. Armitage NH, Kramer MK, Nelson MS, Hopkins D, Langeslay R, Thornton JA. Effectiveness of Lifestyle Interventions in an Active Duty Air Force Population. Am J Health Promot. 2021;35(6):784-93.

5. Aro A, Kauppinen A, Kivinen N, Selander T, Kinnunen K, Tuomilehto J, et al. Life Style Intervention Improves Retinopathy Status-The Finnish Diabetes Prevention Study. Nutrients. 2019;11(7).

6. Balducci S, Zanuso S, Nicolucci A, De Feo P, Cavallo S, Cardelli P, et al. Effect of an intensive exercise intervention strategy on modifiable cardiovascular risk factors in subjects with type 2 diabetes mellitus: a randomized controlled trial: the Italian Diabetes and Exercise Study (IDES). Arch Intern Med. 2010;170(20):1794-803.

7. Balk-Moller NC, Poulsen SK, Larsen TM. Effect of a Nine-Month Web- and App-Based Workplace Intervention to Promote Healthy Lifestyle and Weight Loss for Employees in the Social Welfare and Health Care Sector: A Randomized Controlled Trial. J Med Internet Res. 2017;19(4):e108.

8. Baltic A, Baljic R, Radjo I, Mlaco A. Health Effects of the Programmed Physical Activities on Lipid Profile in Peripheral Arterial Disease of the Lower Extremities. Med Arch. 2015;69(5):311-4.

9. Banz WJ, Maher MA, Thompson WG, Bassett DR, Moore W, Ashraf M, et al. Effects of resistance versus aerobic training on coronary artery disease risk factors. Exp Biol Med (Maywood). 2003;228(4):434-40.

10. Barr SI, Costill DL, Fink WJ, Thomas R. Effect of increased training volume on blood lipids and lipoproteins in male collegiate swimmers. Med Sci Sports Exerc. 1991;23(7):795-800.

11. Behall K.M. HJC, Martel G., Scott W.H., Dooly C.R. Comparsion of resistive to aerobic exercise training on cardiovascular risk factores of sedentary, overweight premenopausal and postmenopausal women. Nutrition Research. 2003;23:607-19.

12. Benatti FB, Larsen SA, Kofoed K, Nielsen ST, Harder-Lauridsen NM, Lyngbaek MP, et al. Intermittent Standing but not a Moderate Exercise Bout Reduces Postprandial Glycemia. Med Sci Sports Exerc. 2017;49(11):2305-14.

13. Besnier F, Lenclume V, Gerardin P, Fianu A, Martinez J, Naty N, et al. Individualized Exercise Training at Maximal Fat Oxidation Combined with Fruit and Vegetable-Rich Diet in Overweight or Obese Women: The LIPOXmax-Reunion Randomized Controlled Trial. PLoS One. 2015;10(11):e0139246.

14. Bidwell AJ, Fairchild TJ, Redmond J, Wang L, Keslacy S, Kanaley JA. Physical activity offsets the negative effects of a high-fructose diet. Med Sci Sports Exerc. 2014;46(11):2091-8.

15. Blackford K, Jancey J, Lee AH, James AP, Waddell T, Howat P. Home-based lifestyle intervention for rural adults improves metabolic syndrome parameters and cardiovascular risk factors: A randomised controlled trial. Prev Med. 2016;89:15-22.

16. Bombardier CH, Dyer JR, Burns P, Crane DA, Takahashi MM, Barber J, et al. A tele-health intervention to increase physical fitness in people with spinal cord injury and cardiometabolic disease or risk factors: a pilot randomized controlled trial. Spinal Cord. 2021;59(1):63-73.

17. Bonato M, Turrini F, V DEZ, Meloni A, Plebani M, Brambilla E, et al. A Mobile Application for Exercise Intervention in People Living with HIV. Med Sci Sports Exerc. 2020;52(2):425-33.

18. Bohr DF, Brodie DC, Cheu DH. Effect of electrolytes on arterial muscle contraction. Circulation. 1958;17(4, Part 2):746-9.

19. Borges JH, Carter SJ, Bryan DR, Hunter GR. Exercise training and/or diet on reduction of intra-abdominal adipose tissue and risk factors for cardiovascular disease. Eur J Clin Nutr. 2019;73(7):1063-8.

20. Botero JP, Prado WL, Guerra RL, Speretta GF, Leite RD, Prestes J, et al. Does aerobic exercise intensity affect health-related parameters in overweight women? Clin Physiol Funct Imaging. 2014;34(2):138-42.

21. Bozzetto L, Annuzzi G, Costabile G, Costagliola L, Giorgini M, Alderisio A, et al. A CHO/fibre diet reduces and a MUFA diet increases postprandial lipaemia in type 2 diabetes: no supplementary effects of low-volume physical training. Acta Diabetol. 2014;51(3):385-93.

22. Brobakken MF, Nygard M, Guzey IC, Morken G, Reitan SK, Heggelund J, et al. Aerobic interval training in standard treatment of out-patients with schizophrenia: a randomized controlled trial. Acta Psychiatr Scand. 2019;140(6):498-507.

23. Buchan DS, Ollis S, Young JD, Cooper SM, Shield JP, Baker JS. High intensity interval running enhances measures of physical fitness but not metabolic measures of cardiovascular disease risk in healthy adolescents. BMC Public Health. 2013;13:498.

24. Buttelli ACK, Costa RR, Farinha JB, Fagundes AO, Vieira AF, Barroso BM, et al. Pilates training improves aerobic capacity, but not lipid or lipoprotein levels in elderly women with dyslipidemia: A controlled trial. J Bodyw Mov Ther. 2021;26:227-32.

25. Ledesma Rios NI, Sepulveda Herrera DM, Cardenas Sanchez DL, Manjarres Correa LM. [Not Available]. Nutr Hosp. 2016;33(5):566.

26. Cardoso DF, Marques EA, Leal DV, Ferreira A, Baker LA, Smith AC, et al. Impact of physical activity and exercise on bone health in patients with chronic kidney disease: a systematic review of observational and experimental studies. BMC Nephrol. 2020;21(1):334.

27. Canuto KJ, Aromataris E, Burgess T, Davy C, McKivett A, Schwartzkopff K, et al. A scoping review of Aboriginal and Torres Strait Islander health promotion programs focused on modifying chronic disease risk factors. Health Promot J Austr. 2021;32(1):46-74.

28. Chang JS, Kim TH, Kong ID. Exercise intervention lowers aberrant serum WISP-1 levels with insulin resistance in breast cancer survivors: a randomized controlled trial. Sci Rep. 2020;10(1):10898.

29. Charatcharoenwitthaya P, Kuljiratitikal K, Aksornchanya O, Chaiyasoot K, Bandidniyamanon W, Charatcharoenwitthaya N. Moderate-Intensity Aerobic vs Resistance Exercise and Dietary Modification in Patients With Nonalcoholic Fatty Liver Disease: A Randomized Clinical Trial. Clin Transl Gastroenterol. 2021;12(3):e00316.

30. Chiavarino C, Cavallero E, Rabellino D, Palumbo L, Bianchino C, Gaita F, et al. Mental Fitness for patients with acute coronary syndrome: A randomized clinical trial. Br J Health Psychol. 2016;21(3):584-99.

31. Choo J, Lee J, Cho JH, Burke LE, Sekikawa A, Jae SY. Effects of weight management by exercise modes on markers of subclinical atherosclerosis and cardiometabolic profile among women with abdominal obesity: a randomized controlled trial. BMC Cardiovasc Disord. 2014;14:82.

32. Chung J, Kim K, Hong J, Kong HJ. Effects of prolonged exercise versus multiple short exercise sessions on risk for metabolic syndrome and the atherogenic index in middle-aged obese women: a randomised controlled trial. BMC Womens Health. 2017;17(1):65.

33. Ciolac EG, Bocchi EA, Greve JM, Guimaraes GV. Heart rate response to exercise and cardiorespiratory fitness of young women at high familial risk for hypertension: effects of interval vs continuous training. Eur J Cardiovasc Prev Rehabil. 2011;18(6):824-30.

34. Clark E, Isler C, Strickland D, McMillan AG, Fang X, Kuehn D, et al. Influence of aerobic exercise on maternal lipid levels and offspring morphometrics. Int J Obes (Lond). 2019;43(3):594-602.

35. Coen PM, Menshikova EV, Distefano G, Zheng D, Tanner CJ, Standley RA, et al. Exercise and Weight Loss Improve Muscle Mitochondrial Respiration, Lipid Partitioning, and Insulin Sensitivity After Gastric Bypass Surgery. Diabetes. 2015;64(11):3737-50.

36. Cox KL, Puddey IB, Morton AR, Beilin LJ, Vandongen R, Masarei JR. The combined effects of aerobic exercise and alcohol restriction on blood pressure and serum lipids: a two-way factorial study in sedentary men. J Hypertens. 1993;11(2):191-201.

37. Cox KL, Burke V, Beilin LJ, Puddey IB. A comparison of the effects of swimming and walking on body weight, fat distribution, lipids, glucose, and insulin in older women--the Sedentary Women Exercise Adherence Trial 2. Metabolism. 2010;59(11):1562-73.

38. Crouter SE, de Ferranti SD, Whiteley J, Steltz SK, Osganian SK, Feldman HA, et al. Effect on Physical Activity of a Randomized Afterschool Intervention for Inner City Children in 3rd to 5th Grade. PLoS One. 2015;10(10):e0141584.

39. Cullberg KB, Christiansen T, Paulsen SK, Bruun JM, Pedersen SB, Richelsen B. Effect of weight loss and exercise on angiogenic factors in the circulation and in adipose tissue in obese subjects. Obesity (Silver Spring). 2013;21(3):454-60.

40. da Silva Soares DB, Shinjo SK, Santos AS, de Cassia Rosa de Jesus J, Schenk S, de Castro GS, et al. Skeletal muscle gene expression in older adults with type 2 diabetes mellitus undergoing calorie-restricted diet and recreational sports training - a randomized clinical trial. Exp Gerontol. 2022;164:111831.

41. Dai X, Zhai L, Chen Q, Miller JD, Lu L, Hsue C, et al. Two-year-supervised resistance training prevented diabetes incidence in people with prediabetes: A randomised control trial. Diabetes Metab Res Rev. 2019;35(5):e3143.

42. Dawson JK, Dorff TB, Todd Schroeder E, Lane CJ, Gross ME, Dieli-Conwright CM. Impact of resistance training on body composition and metabolic syndrome variables during androgen deprivation therapy for prostate cancer: a pilot randomized controlled trial. BMC Cancer. 2018;18(1):368.

43. de Meirelles LR, Matsuura C, Resende Ade C, Salgado AA, Pereira NR, Coscarelli PG, et al. Chronic exercise leads to antiaggregant, antioxidant and anti-inflammatory effects in heart failure patients. Eur J Prev Cardiol. 2014;21(10):1225-32.

44. de Sousa MV, Fukui R, Krustrup P, Pereira RM, Silva PR, Rodrigues AC, et al. Positive effects of football on fitness, lipid profile, and insulin resistance in Brazilian patients with type 2 diabetes. Scand J Med Sci Sports. 2014;24 Suppl 1:57-65.

45. De Strijcker D, Lapauw B, Ouwens DM, Van de Velde D, Hansen D, Petrovic M, et al. High intensity interval training is associated with greater impact on physical fitness, insulin sensitivity and muscle mitochondrial content in males with overweight/obesity, as opposed to continuous endurance training: a randomized controlled trial. J Musculoskelet Neuronal Interact. 2018;18(2):215-26.

46. del Pozo-Cruz B, Alfonso-Rosa RM, del Pozo-Cruz J, Sanudo B, Rogers ME. Effects of a 12-wk whole-body vibration based intervention to improve type 2 diabetes. Maturitas. 2014;77(1):52-8.

47. Delevatti RS, Kanitz AC, Alberton CL, Marson EC, Lisboa SC, Pinho CD, et al. Glucose control can be similarly improved after aquatic or dry-land aerobic training in patients with type 2 diabetes: A randomized clinical trial. J Sci Med Sport. 2016;19(8):688-93.

48. Delgado-Floody P, Alvarez C, Lusa Cadore E, Flores-Opazo M, Caamano-Navarrete F, Izquierdo M. Preventing metabolic syndrome in morbid obesity with resistance training: Reporting interindividual variability. Nutr Metab Cardiovasc Dis. 2019;29(12):1368-81.

49. Dennis BA, Ergul A, Gower BA, Allison JD, Davis CL. Oxidative stress and cardiovascular risk in overweight children in an exercise intervention program. Child Obes. 2013;9(1):15-21.

50. DiPietro L, Dziura J, Yeckel CW, Neufer PD. Exercise and improved insulin sensitivity in older women: evidence of the enduring benefits of higher intensity training. J Appl Physiol (1985). 2006;100(1):142-9.

51. Duncan GE, Anton SD, Sydeman SJ, Newton RL, Jr., Corsica JA, Durning PE, et al. Prescribing exercise at varied levels of intensity and frequency: a randomized trial. Arch Intern Med. 2005;165(20):2362-9.

52. Ehret CJ, Zhou S, Tchou JC, Schmitz KH, Sturgeon KM. Dose-dependent effects of aerobic exercise on clinically relevant biomarkers among healthy women at high genetic risk for breast cancer: A secondary analysis of a randomized controlled study. Cancer Rep (Hoboken). 2022;5(5):e1497.

53. Elme A, Utriainen M, Kellokumpu-Lehtinen P, Palva T, Luoto R, Nikander R, et al. Obesity and physical inactivity are related to impaired physical health of breast cancer survivors. Anticancer Res. 2013;33(4):1595-602.

54. Espeland MA, Rejeski WJ, West DS, Bray GA, Clark JM, Peters AL, et al. Intensive weight loss intervention in older individuals: results from the Action for Health in Diabetes Type 2 diabetes mellitus trial. J Am Geriatr Soc. 2013;61(6):912-22.

55. Farag HAM, Hosseinzadeh-Attar MJ, Muhammad BA, Esmaillzadeh A, Hamid El Bilbeisi A. Effects of vitamin D supplementation along with endurance physical activity on lipid profile in metabolic syndrome patients: A randomized controlled trial. Diabetes Metab Syndr. 2019;13(2):1093-8.

56. Faria WF, Mendonca FR, Santos GC, Kennedy SG, Elias RGM, Stabelini Neto A. Effects of 2 Methods of Combined Training on Cardiometabolic Risk Factors in Adolescents: A Randomized Controlled Trial. Pediatr Exerc Sci. 2020;32(4):217-26.

57. Faulkner J, Lambrick D, Woolley B, Stoner L, Wong LK, McGonigal G. Effects of early exercise engagement on vascular risk in patients with transient ischemic attack and nondisabling stroke. J Stroke Cerebrovasc Dis. 2013;22(8):e388-96.

58. Fernandez-Ruiz VE, Sole-Agusti M, Armero-Barranco D, Cauli O. Weight Loss and Improvement of Metabolic Alterations in Overweight and Obese Children Through the I(2)AO(2) Family Program: A Randomized Controlled Clinical Trial. Biol Res Nurs. 2021;23(3):488-503.

59. Fisher G, Brown AW, Bohan Brown MM, Alcorn A, Noles C, Winwood L, et al. High Intensity Interval- vs Moderate Intensity- Training for Improving Cardiometabolic Health in Overweight or Obese Males: A Randomized Controlled Trial. PLoS One. 2015;10(10):e0138853.

60. Frederix I, Van Driessche N, Hansen D, Berger J, Bonne K, Alders T, et al. Increasing the medium-term clinical benefits of hospital-based cardiac rehabilitation by physical activity telemonitoring in coronary artery disease patients. Eur J Prev Cardiol. 2015;22(2):150-8.

61. Freedland SJ, Howard L, Allen J, Smith J, Stout J, Aronson W, et al. A lifestyle intervention of weight loss via a low-carbohydrate diet plus walking to reduce metabolic disturbances caused by androgen deprivation therapy among prostate cancer patients: carbohydrate and prostate study 1 (CAPS1) randomized controlled trial. Prostate Cancer Prostatic Dis. 2019;22(3):428-37.

62. Galvao DA, Spry N, Denham J, Taaffe DR, Cormie P, Joseph D, et al. A multicentre year-long randomised controlled trial of exercise training targeting physical functioning in men with prostate cancer previously treated with androgen suppression and radiation from TROG 03.04 RADAR. Eur Urol. 2014;65(5):856-64.

63. Garavelo JJ, Altimari LR, Gabardo JM, Ferreira-Junior A, Freitas LAG, Buzzachera CF, et al. Effects of walk training with self-selected intensity on biochemical markers and anthropometric variables in women with obesity. Obes Res Clin Pract. 2019;13(2):211-3.

64. Garner S, Fenton T, Martin L, Creaser C, Johns C, Barnabe C. Personalized diet and exercise recommendations in early rheumatoid arthritis: A feasibility trial. Musculoskeletal Care. 2018;16(1):167-72.

65. Giallauria F, Gentile M, Chiodini P, Berrino F, Mattiello A, Maresca L, et al. Exercise training reduces high mobility group box-1 protein levels in women with breast cancer: findings from the DIANA-5 study. Monaldi Arch Chest Dis. 2014;82(2):61-7.

66. Gonzalez-Ruiz K, Correa-Bautista JE, Izquierdo M, Garcia-Hermoso A, Martinez-Vizcaino V, Lobelo F, et al. Exercise dose on hepatic fat and cardiovascular health in adolescents with excess of adiposity. Pediatr Obes. 2022;17(4):e12869.

67. Gouveia S. GG, Souza M, Costa B, Pinho V et al. The effect of pilates on metabolic control and oxidative stress of diabetics type 2 e A randomized controlled clinical trial. Journal of Bodywork & Movement Therapies. 2021;27(2021):60-6.

68. Hagner-Derengowska M, Kaluzny K, Kochanski B, Hagner W, Borkowska A, Czamara A, et al. Effects of Nordic Walking and Pilates exercise programs on blood glucose and lipid profile in overweight and obese postmenopausal women in an experimental, nonrandomized, open-label, prospective controlled trial. Menopause. 2015;22(11):1215-23.

69. Halverstadt A, Phares DA, Ferrell RE, Wilund KR, Goldberg AP, Hagberg JM. High-density lipoprotein-cholesterol, its subfractions, and responses to exercise training are dependent on endothelial lipase genotype. Metabolism. 2003;52(11):1505-11.

70. Harreiter J, Simmons D, Desoye G, Corcoy R, Adelantado JM, Devlieger R, et al. Nutritional Lifestyle Intervention in Obese Pregnant Women, Including Lower Carbohydrate Intake, Is Associated With Increased Maternal Free Fatty Acids, 3-beta-Hydroxybutyrate, and Fasting Glucose Concentrations: A Secondary Factorial Analysis of the European Multicenter, Randomized Controlled DALI Lifestyle Intervention Trial. Diabetes Care. 2019;42(8):1380-9.

71. Hiruntrakul A, Nanagara R, Emasithi A, Borer KT. Effect of once a week endurance exercise on fitness status in sedentary subjects. Journal of the Medical Association of Thailand. 2011;93(9):1070.

72. Hojan K, Kwiatkowska-Borowczyk E, Leporowska E, Milecki P. Inflammation, cardiometabolic markers, and functional changes in men with prostate cancer. A randomized controlled trial of a 12‑month exercise program. Pol Arch Intern Med. 2017;127(1):25-35.

73. Howden EJ, Leano R, Petchey W, Coombes JS, Isbel NM, Marwick TH. Effects of exercise and lifestyle intervention on cardiovascular function in CKD. Clin J Am Soc Nephrol. 2013;8(9):1494-501.

74. Hsu YI, Chen YC, Lee CL, Chang NJ. Effects of Diet Control and Telemedicine-Based Resistance Exercise Intervention on Patients with Obesity and Knee Osteoarthritis: A Randomized Control Trial. Int J Environ Res Public Health. 2021;18(15).

75. Hvid T, Lindegaard B, Winding K, Iversen P, Brasso K, Solomon TP, et al. Effect of a 2-year home-based endurance training intervention on physiological function and PSA doubling time in prostate cancer patients. Cancer Causes Control. 2016;27(2):165-74.

76. Inoue DS, De Mello MT, Foschini D, Lira FS, De Piano Ganen A, Da Silveira Campos RM, et al. Linear and undulating periodized strength plus aerobic training promote similar benefits and lead to improvement of insulin resistance on obese adolescents. J Diabetes Complications. 2015;29(2):258-64.

77. Jackson M, Fatahi F, Alabduljader K, Jelleyman C, Moore JP, Kubis HP. Exercise training and weight loss, not always a happy marriage: single blind exercise trials in females with diverse BMI. Appl Physiol Nutr Metab. 2018;43(4):363-70.

78. Jones LW, Hornsby WE, Freedland SJ, Lane A, West MJ, Moul JW, et al. Effects of nonlinear aerobic training on erectile dysfunction and cardiovascular function following radical prostatectomy for clinically localized prostate cancer. Eur Urol. 2014;65(5):852-5.

79. Joseph LJ, Davey SL, Evans WJ, Campbell WW. Differential effect of resistance training on the body composition and lipoprotein-lipid profile in older men and women. Metabolism. 1999;48(11):1474-80.

80. Joubert J, Davis SM, Donnan GA, Levi C, Gonzales G, Joubert L, et al. ICARUSS: An effective model for risk factor management in stroke survivors. Int J Stroke. 2020;15(4):438-53.

81. Juliana N, Shahar S, Sahar MA, Ghazali AR, Manaf ZA, Noah RM. 'Her shape' intervention programme for obese women with high breast adiposity. Asia Pac J Clin Nutr. 2017;26(2):278-86.

82. Jurczak I, Kowalski J, Irzmanski R. Impact of cardiac rehabilitation on peripheral circulation as assessed by impedance plethysmography: a randomized clinical trial. Eur J Phys Rehabil Med. 2014;50(6):609-16.

83. Kanaya AM, Araneta MR, Pawlowsky SB, Barrett-Connor E, Grady D, Vittinghoff E, et al. Restorative yoga and metabolic risk factors: the Practicing Restorative Yoga vs. Stretching for the Metabolic Syndrome (PRYSMS) randomized trial. J Diabetes Complications. 2014;28(3):406-12.

84. Karstoft K, Winding K, Knudsen SH, Nielsen JS, Thomsen C, Pedersen BK, et al. The effects of free-living interval-walking training on glycemic control, body composition, and physical fitness in type 2 diabetic patients: a randomized, controlled trial. Diabetes Care. 2013;36(2):228-36.

85. Katzel LI, Bleecker ER, Rogus EM, Goldberg AP. Sequential effects of aerobic exercise training and weight loss on risk factors for coronary disease in healthy, obese middle-aged and older men. Metabolism. 1997;46(12):1441-7.

86. Keadle SK, Shiroma EJ, Freedson PS, Lee IM. Impact of accelerometer data processing decisions on the sample size, wear time and physical activity level of a large cohort study. BMC Public Health. 2014;14:1210.

87. Kerling A, Tegtbur U, Gutzlaff E, Kuck M, Borchert L, Ates Z, et al. Effects of adjunctive exercise on physiological and psychological parameters in depression: a randomized pilot trial. J Affect Disord. 2015;177:1-6.

88. Killeen SL, Yelverton CA, Geraghty AA, Kennelly MA, Eakins S, Farrell L, et al. The Edmonton Obesity Staging System and pregnancy outcomes in women with overweight or obesity: A secondary analysis of a randomized controlled trial. Clin Obes. 2022;12(3):e12510.

89. Kim DI, Lee H, Lee BS, Kim J, Jeon JY. Effects of a 6-Week Indoor Hand-Bike Exercise Program on Health and Fitness Levels in People With Spinal Cord Injury: A Randomized Controlled Trial Study. Arch Phys Med Rehabil. 2015;96(11):2033-40 e1.

90. Kim HB, Hyun AH. Psychological and Biochemical Effects of an Online Pilates Intervention in Pregnant Women during COVID-19: A Randomized Pilot Study. Int J Environ Res Public Health. 2022;19(17).

91. King AC, Haskell WL, Young DR, Oka RK, Stefanick ML. Long-term effects of varying intensities and formats of physical activity on participation rates, fitness, and lipoproteins in men and women aged 50 to 65 years. Circulation. 1995;91(10):2596-604.

92. Kirk H, Kersten P, Crawford P, Keens A, Ashburn A, Conway J. The cardiac model of rehabilitation for reducing cardiovascular risk factors post transient ischaemic attack and stroke: a randomized controlled trial. Clin Rehabil. 2014;28(4):339-49.

93. Kleinloog JPD, Mensink RP, Roodt JO, Thijssen DHJ, Hesselink MKC, Joris PJ. Aerobic exercise training improves not only brachial artery flow-mediated vasodilatation but also carotid artery reactivity: A randomized controlled, cross-over trial in older men. Physiol Rep. 2022;10(16):e15395.

94. Kokkinos PF, Narayan P, Colleran J, Fletcher RD, Lakshman R, Papademetriou V. Effects of moderate intensity exercise on serum lipids in African-American men with severe systemic hypertension. Am J Cardiol. 1998;81(6):732-5.

95. Kong Z, Fan X, Sun S, Song L, Shi Q, Nie J. Comparison of High-Intensity Interval Training and Moderate-to-Vigorous Continuous Training for Cardiometabolic Health and Exercise Enjoyment in Obese Young Women: A Randomized Controlled Trial. PLoS One. 2016;11(7):e0158589.

96. Kono Y, Yamada S, Yamaguchi J, Hagiwara Y, Iritani N, Ishida S, et al. Secondary prevention of new vascular events with lifestyle intervention in patients with noncardioembolic mild ischemic stroke: a single-center randomized controlled trial. Cerebrovasc Dis. 2013;36(2):88-97.

97. Kraemer WJ, Volek JS, Clark KL, Gordon SE, Incledon T, Puhl SM, et al. Physiological adaptations to a weight-loss dietary regimen and exercise programs in women. J Appl Physiol (1985). 1997;83(1):270-9.

98. Kraemer WJ, Volek JS, Clark KL, Gordon SE, Puhl SM, Koziris LP, et al. Influence of exercise training on physiological and performance changes with weight loss in men. Med Sci Sports Exerc. 1999;31(9):1320-9.

99. Krogh J, Videbech P, Thomsen C, Gluud C, Nordentoft M. DEMO-II trial. Aerobic exercise versus stretching exercise in patients with major depression-a randomised clinical trial. PLoS One. 2012;7(10):e48316.

100. Kuo YC, Chang HL, Cheng CF, Mundel T, Liao YH. Six-week inspiratory resistance training ameliorates endurance performance but does not affect obesity-related metabolic biomarkers in obese adults: A randomized controlled trial. Respir Physiol Neurobiol. 2020;273:103285.

101. Lee MA. [A comparative study of how subjects' characteristics and nursing service quality influence on hospital revisiting intent between patients and nurses]. Taehan Kanho Hakhoe Chi. 2005;35(7):1210-20.

102. Lima RA, Andersen LB, Soares FC, Kriemler S. The causal pathway effects of a physical activity intervention on adiposity in children: The KISS Study cluster randomized clinical trial. Scand J Med Sci Sports. 2020;30(9):1685-91.

103. Lindheim SR, Notelovitz M, Feldman EB, Larsen S, Khan FY, Lobo RA. The independent effects of exercise and estrogen on lipids and lipoproteins in postmenopausal women. Obstet Gynecol. 1994;83(2):167-72.

104. Liu L, Ma X, Xu H, Ruan S, Yuan X. Comparing the effects of 12 months aerobic exercise and resistance training on glucose metabolism among prediabetes phenotype: A explorative randomized controlled trial. Prim Care Diabetes. 2021;15(2):340-6.

105. Liu X, Zhou W, Fan W, Li A, Pang J, Chen Z, et al. The benefit of exercise rehabilitation guided by 6-minute walk test on lipoprotein-associated phospholipase A2 in patients with coronary heart disease undergoing percutaneous coronary intervention: a prospective randomized controlled study. BMC Cardiovasc Disord. 2022;22(1):177.

106. Lockard B, Mardock M, Oliver JM, Byrd M, Simbo S, Jagim AR, et al. Comparison of Two Diet and Exercise Approaches on Weight Loss and Health Outcomes in Obese Women. Int J Environ Res Public Health. 2022;19(8).

107. Lopes WA, Leite N, da Silva LR, Brunelli DT, Gaspari AF, Radominski RB, et al. Effects of 12 weeks of combined training without caloric restriction on inflammatory markers in overweight girls. J Sports Sci. 2016;34(20):1902-12.

108. MacKay-Lyons M, Gubitz G, Phillips S, Giacomantonio N, Firth W, Thompson K, et al. Program of Rehabilitative Exercise and Education to Avert Vascular Events After Non-Disabling Stroke or Transient Ischemic Attack (PREVENT Trial): A Randomized Controlled Trial. Neurorehabil Neural Repair. 2022;36(2):119-30.

109. Malmo V, Nes BM, Amundsen BH, Tjonna AE, Stoylen A, Rossvoll O, et al. Aerobic Interval Training Reduces the Burden of Atrial Fibrillation in the Short Term: A Randomized Trial. Circulation. 2016;133(5):466-73.

110. Marcotte-Chenard A, Tremblay D, Mony MM, Brochu M, Dionne IJ, Langlois MF, et al. Low-volume walking HIIT: Efficient strategy to improve physical capacity and reduce the risk of cardiovascular disease in older women with type 2 diabetes. Diabetes Metab Syndr. 2021;15(5):102233.

111. Matsugaki R, Kuhara S, Saeki S, Jiang Y, Michishita R, Ohta M, et al. Effectiveness of workplace exercise supervised by a physical therapist among nurses conducting shift work: A randomized controlled trial. J Occup Health. 2017;59(4):327-35.

112. Matsuo T, So R, Shimojo N, Tanaka K. Effect of aerobic exercise training followed by a low-calorie diet on metabolic syndrome risk factors in men. Nutr Metab Cardiovasc Dis. 2015;25(9):832-8.

113. McAuley KA, Williams SM, Mann JI, Goulding A, Chisholm A, Wilson N, et al. Intensive lifestyle changes are necessary to improve insulin sensitivity: a randomized controlled trial. Diabetes Care. 2002;25(3):445-52.

114. McCormack SE, McCarthy MA, Harrington SG, Farilla L, Hrovat MI, Systrom DM, et al. Effects of exercise and lifestyle modification on fitness, insulin resistance, skeletal muscle oxidative phosphorylation and intramyocellular lipid content in obese children and adolescents. Pediatr Obes. 2014;9(4):281-91.

115. Meng C, Yucheng T, Shu L, Yu Z. Effects of school-based high-intensity interval training on body composition, cardiorespiratory fitness and cardiometabolic markers in adolescent boys with obesity: a randomized controlled trial. BMC Pediatr. 2022;22(1):112.

116. Michael E, White MJ, Eves FF. Home-Based Stair Climbing as an Intervention for Disease Risk in Adult Females; A Controlled Study. Int J Environ Res Public Health. 2021;18(2).

117. Moholdt T, Parr EB, Devlin BL, Debik J, Giskeodegard G, Hawley JA. The effect of morning vs evening exercise training on glycaemic control and serum metabolites in overweight/obese men: a randomised trial. Diabetologia. 2021;64(9):2061-76.

118. Moore SA, Hallsworth K, Jakovljevic DG, Blamire AM, He J, Ford GA, et al. Effects of Community Exercise Therapy on Metabolic, Brain, Physical, and Cognitive Function Following Stroke: A Randomized Controlled Pilot Trial. Neurorehabil Neural Repair. 2015;29(7):623-35.

119. Moro T, Marcolin G, Bianco A, Bolzetta F, Berton L, Sergi G, et al. Effects of 6 Weeks of Traditional Resistance Training or High Intensity Interval Resistance Training on Body Composition, Aerobic Power and Strength in Healthy Young Subjects: A Randomized Parallel Trial. Int J Environ Res Public Health. 2020;17(11).

120. Muros JJ, Zabala M, Oliveras-Lopez MJ, Ocana-Lara FA, Lopez-Garcia de la Serrana H. Results of a 7-week school-based physical activity and nutrition pilot program on health-related parameters in primary school children in southern Spain. Pediatr Exerc Sci. 2013;25(2):248-61.

121. Nah EH, Chu J, Kim S, Cho S, Kwon E. Efficacy of lifestyle interventions in the reversion to normoglycemia in Korean prediabetics: One-year results from a randomised controlled trial. Prim Care Diabetes. 2019;13(3):212-20.

122. Nicklas BJ, Wang X, You T, Lyles MF, Demons J, Easter L, et al. Effect of exercise intensity on abdominal fat loss during calorie restriction in overweight and obese postmenopausal women: a randomized, controlled trial. Am J Clin Nutr. 2009;89(4):1043-52.

123. Nieman DC, Nehlsen-Cannarella SL, Henson DA, Koch AJ, Butterworth DE, Fagoaga OR, et al. Immune response to exercise training and/or energy restriction in obese women. Med Sci Sports Exerc. 1998;30(5):679-86.

124. Noites A, Vale AL, Pereira AS, Morais A, Vilarinho R, Carvalho P, et al. Effect of an aerobic exercise session combined with abdominal radiofrequency on lipolytic activity in women: Randomized control trial. J Cosmet Dermatol. 2020;19(3):638-45.

125. Nono Nankam PA, Mendham AE, van Jaarsveld PJ, Adams K, Fortuin-de Smidt MC, Clamp L, et al. Exercise Training Alters Red Blood Cell Fatty Acid Desaturase Indices and Adipose Tissue Fatty Acid Profile in African Women with Obesity. Obesity (Silver Spring). 2020;28(8):1456-66.

126. Nooijen CF, Stam HJ, Sluis T, Valent L, Twisk J, van den Berg-Emons RJ. A behavioral intervention promoting physical activity in people with subacute spinal cord injury: secondary effects on health, social participation and quality of life. Clin Rehabil. 2017;31(6):772-80.

127. Nybacka A, Hellstrom PM, Hirschberg AL. Increased fibre and reduced trans fatty acid intake are primary predictors of metabolic improvement in overweight polycystic ovary syndrome-Substudy of randomized trial between diet, exercise and diet plus exercise for weight control. Clin Endocrinol (Oxf). 2017;87(6):680-8.

128. O'Doherty AF, Sathyapalan T, Rigby AS, Ingle L, Carroll S. The repeatability of the abbreviated (4-h) Oral Fat Tolerance Test and influence of prior acute aerobic exercise. Eur J Nutr. 2018;57(1):309-18.

129. Onagbiye SO, Moss SJ, Cameron M. Managing Noncommunicable Diseases in an African Community: Effects, Compliance, and Barriers to Participation in a 4-Week Exercise Intervention. Int Q Community Health Educ. 2016;36(3):165-76.

130. Ordonez FJ, Rosety MA, Camacho A, Rosety I, Diaz AJ, Fornieles G, et al. Arm-cranking exercise reduced oxidative damage in adults with chronic spinal cord injury. Arch Phys Med Rehabil. 2013;94(12):2336-41.

131. Pablos A, Nebot V, Vano-Vicent V, Ceca D, Elvira L. Effectiveness of a school-based program focusing on diet and health habits taught through physical exercise. Appl Physiol Nutr Metab. 2018;43(4):331-7.

132. Paschali AA, Peppou L, Benroubi M. Relaxation training significantly reduced blood glucose levels in patients with type 1 diabetes mellitus. Hormones (Athens). 2020;19(2):215-22.

133. Pedersen LR, Olsen RH, Jurs A, Astrup A, Chabanova E, Simonsen L, et al. A randomised trial comparing weight loss with aerobic exercise in overweight individuals with coronary artery disease: The CUT-IT trial. Eur J Prev Cardiol. 2015;22(8):1009-17.

134. Pedersen LR, Olsen RH, Anholm C, Astrup A, Eugen-Olsen J, Fenger M, et al. Effects of 1 year of exercise training versus combined exercise training and weight loss on body composition, low-grade inflammation and lipids in overweight patients with coronary artery disease: a randomized trial. Cardiovasc Diabetol. 2019;18(1):127.

135. Poeta LS, Duarte Mde F, Caramelli B, Jorge M, Giuliano Ide C. Effects of physical exercises and nutritional guidance on the cardiovascular risk profile of obese children. Rev Assoc Med Bras (1992). 2013;59(1):56-63.

136. Ponjee GA, Janssen EM, Hermans J, van Wersch JW. Effects of long-term exercise of moderate intensity on anthropometric values and serum lipids and lipoproteins. Eur J Clin Chem Clin Biochem. 1995;33(3):121-6.

137. Prince SA, Reed JL, Cotie LM, Harris J, Pipe AL, Reid RD. Results of the Sedentary Intervention Trial in Cardiac Rehabilitation (SIT-CR Study): A pilot randomized controlled trial. Int J Cardiol. 2018;269:317-24.

138. Qazi WA, Babur MN, Malik AN, Bokhari NA, Baig MS, Begum R. Effects of structured exercise regime on lipid profile and renal function tests in gestational diabetes mellitus patients:A pilot study. J Pak Med Assoc. 2021;71(2(A)):505-7.

139. Ramirez-Velez R, Castro-Astudillo K, Correa-Bautista JE, Gonzalez-Ruiz K, Izquierdo M, Garcia-Hermoso A, et al. The Effect of 12 Weeks of Different Exercise Training Modalities or Nutritional Guidance on Cardiometabolic Risk Factors, Vascular Parameters, and Physical Fitness in Overweight Adults: Cardiometabolic High-Intensity Interval Training-Resistance Training Randomized Controlled Study. J Strength Cond Res. 2020;34(8):2178-88.

140. Ratajczak M, Skrypnik D, Bogdanski P, Madry E, Walkowiak J, Szulinska M, et al. Effects of Endurance and Endurance-Strength Training on Endothelial Function in Women with Obesity: A Randomized Trial. Int J Environ Res Public Health. 2019;16(21).

141. Rech A, Botton CE, Lopez P, Quincozes-Santos A, Umpierre D, Pinto RS. Effects of short-term resistance training on endothelial function and inflammation markers in elderly patients with type 2 diabetes: A randomized controlled trial. Exp Gerontol. 2019;118:19-25.

142. Resaland GK, Aadland E, Nilsen AKO, Bartholomew JB, Andersen LB, Anderssen SA. The effect of a two-year school-based daily physical activity intervention on a clustered CVD risk factor score-The Sogndal school-intervention study. Scand J Med Sci Sports. 2018;28(3):1027-35.

143. Rivas E, Crandall CG, Suman OE, Moustaid-Moussa N, Ben-Ezra V. Exercise heat acclimation causes post-exercise hypotension and favorable improvements in lipid and immune profiles: A crossover randomized controlled trial. J Therm Biol. 2019;84:266-73.

144. Rodriguez J, Neyrinck AM, Van Kerckhoven M, Gianfrancesco MA, Renguet E, Bertrand L, et al. Physical activity enhances the improvement of body mass index and metabolism by inulin: a multicenter randomized placebo-controlled trial performed in obese individuals. BMC Med. 2022;20(1):110.

145. Rodrigues-Krause J, Farinha JB, Ramis TR, Macedo RCO, Boeno FP, Dos Santos GC, et al. Effects of dancing compared to walking on cardiovascular risk and functional capacity of older women: A randomized controlled trial. Exp Gerontol. 2018;114:67-77.

146. Rosa TS, Correa HL, Deus LA, Stone W, Reis AL, Gadelha AB, et al. Effects of dynamic and isometric resistance training protocols on metabolic profile in hemodialysis patients: a randomized controlled trial. Appl Physiol Nutr Metab. 2021;46(9):1029-37.

147. Rosenberg A, Ngandu T, Rusanen M, Antikainen R, Backman L, Havulinna S, et al. Multidomain lifestyle intervention benefits a large elderly population at risk for cognitive decline and dementia regardless of baseline characteristics: The FINGER trial. Alzheimers Dement. 2018;14(3):263-70.

148. Ruffino JS, Songsorn P, Haggett M, Edmonds D, Robinson AM, Thompson D, et al. A comparison of the health benefits of reduced-exertion high-intensity interval training (REHIT) and moderate-intensity walking in type 2 diabetes patients. Appl Physiol Nutr Metab. 2017;42(2):202-8.

149. Ryan BJ, Schleh MW, Ahn C, Ludzki AC, Gillen JB, Varshney P, et al. Moderate-Intensity Exercise and High-Intensity Interval Training Affect Insulin Sensitivity Similarly in Obese Adults. J Clin Endocrinol Metab. 2020;105(8):e2941-59.

150. Salahshoornezhad S, Sohrabi Z, Mani A, Abdelbasset WK, Mehrabi M, Zare M, et al. Effect of a multi-disciplinary program on anthropometric and biochemical parameters in obese and overweight elementary school girls: A randomized clinical trial. Nutr Metab Cardiovasc Dis. 2022;32(8):1982-9.

151. Scott JM, Martin D, Ploutz-Snyder R, Downs M, Dillon EL, Sheffield-Moore M, et al. Efficacy of Exercise and Testosterone to Mitigate Atrophic Cardiovascular Remodeling. Med Sci Sports Exerc. 2018;50(9):1940-9.

152. Seguin-Fowler R, Graham M, Sriram U, Eldridge G, Kim J, Tom M. Web-Based Dissemination of a Civic Engagement Curriculum to Promote Healthy Eating and Active Living in Rural Towns: The eHEART Study. Int J Environ Res Public Health. 2020;17(7).

153. Seyam MK, Alqahtani M, Sirajudeen MS, Muthusamy H, Kashoo FZ, Salah MM. Effect of circuit training with low-carbohydrate diet on body composition, cardiometabolic indices, and exercise capacity in adults with mild to moderate obesity in Saudi Arabia: A randomized control trial. Medicine (Baltimore). 2022;101(33):e30054.

154. Sheikholeslami Vatani D, Ahmadi S, Ahmadi Dehrashid K, Gharibi F. Changes in cardiovascular risk factors and inflammatory markers of young, healthy, men after six weeks of moderate or high intensity resistance training. J Sports Med Phys Fitness. 2011;51(4):695-700.

155. Shepherd SO, Wilson OJ, Taylor AS, Thogersen-Ntoumani C, Adlan AM, Wagenmakers AJ, et al. Low-Volume High-Intensity Interval Training in a Gym Setting Improves Cardio-Metabolic and Psychological Health. PLoS One. 2015;10(9):e0139056.

156. Siddiqui F, Kurbasic A, Lindblad U, Nilsson PM, Bennet L. Effects of a culturally adapted lifestyle intervention on cardio-metabolic outcomes: a randomized controlled trial in Iraqi immigrants to Sweden at high risk for Type 2 diabetes. Metabolism. 2017;66:1-13.

157. Sigal RJ, Alberga AS, Goldfield GS, Prud'homme D, Hadjiyannakis S, Gougeon R, et al. Effects of aerobic training, resistance training, or both on percentage body fat and cardiometabolic risk markers in obese adolescents: the healthy eating aerobic and resistance training in youth randomized clinical trial. JAMA Pediatr. 2014;168(11):1006-14.

158. Sillanpaa E, Hakkinen A, Laaksonen DE, Karavirta L, Kraemer WJ, Hakkinen K. Serum basal hormone concentrations, nutrition and physical fitness during strength and/or endurance training in 39-64-year-old women. Int J Sports Med. 2010;31(2):110-7.

159. Simpson EJ, Debevec T, Eiken O, Mekjavic I, Macdonald IA. PlanHab: the combined and separate effects of 16 days of bed rest and normobaric hypoxic confinement on circulating lipids and indices of insulin sensitivity in healthy men. J Appl Physiol (1985). 2016;120(8):947-55.

160. Sjoros TJ, Heiskanen MA, Motiani KK, Loyttyniemi E, Eskelinen JJ, Virtanen KA, et al. Increased insulin-stimulated glucose uptake in both leg and arm muscles after sprint interval and moderate-intensity training in subjects with type 2 diabetes or prediabetes. Scand J Med Sci Sports. 2018;28(1):77-87.

161. Slentz CA, Aiken LB, Houmard JA, Bales CW, Johnson JL, Tanner CJ, et al. Inactivity, exercise, and visceral fat. STRRIDE: a randomized, controlled study of exercise intensity and amount. J Appl Physiol (1985). 2005;99(4):1613-8.

162. Slentz CA, Bateman LA, Willis LH, Granville EO, Piner LW, Samsa GP, et al. Effects of exercise training alone vs a combined exercise and nutritional lifestyle intervention on glucose homeostasis in prediabetic individuals: a randomised controlled trial. Diabetologia. 2016;59(10):2088-98.

163. Solomon A, Turunen H, Ngandu T, Peltonen M, Levalahti E, Helisalmi S, et al. Effect of the Apolipoprotein E Genotype on Cognitive Change During a Multidomain Lifestyle Intervention: A Subgroup Analysis of a Randomized Clinical Trial. JAMA Neurol. 2018;75(4):462-70.

164. Song Y, Ren C, Liu P, Tao L, Zhao W, Gao W. Effect of Smartphone-Based Telemonitored Exercise Rehabilitation among Patients with Coronary Heart Disease. J Cardiovasc Transl Res. 2020;13(4):659-67.

165. Sopko G, Leon AS, Jacobs DR, Jr., Foster N, Moy J, Kuba K, et al. The effects of exercise and weight loss on plasma lipids in young obese men. Metabolism. 1985;34(3):227-36.

166. Sparks LM, Johannsen NM, Church TS, Earnest CP, Moonen-Kornips E, Moro C, et al. Nine months of combined training improves ex vivo skeletal muscle metabolism in individuals with type 2 diabetes. J Clin Endocrinol Metab. 2013;98(4):1694-702.

167. Staiano AE, Beyl RA, Guan W, Hendrick CA, Hsia DS, Newton RL, Jr. Home-based exergaming among children with overweight and obesity: a randomized clinical trial. Pediatr Obes. 2018;13(11):724-33.

168. Stavnsbo M, Aadland E, Anderssen SA, Chinapaw M, Steene-Johannessen J, Andersen LB, et al. Effects of the Active Smarter Kids (ASK) physical activity intervention on cardiometabolic risk factors in children: A cluster-randomized controlled trial. Prev Med. 2020;130:105868.

169. Sung K, Bae S. Effects of a regular walking exercise program on behavioral and biochemical aspects in elderly people with type II diabetes. Nurs Health Sci. 2012;14(4):438-45.

170. Suzuki T, Shimada H, Makizako H, Doi T, Yoshida D, Ito K, et al. A randomized controlled trial of multicomponent exercise in older adults with mild cognitive impairment. PLoS One. 2013;8(4):e61483.

171. Swoboda CM, Miller CK, Wills CE. Setting Single or Multiple Goals for Diet and Physical Activity Behaviors Improves Cardiovascular Disease Risk Factors in Adults With Type 2 Diabetes: A Pragmatic Pilot Randomized Trial. Diabetes Educ. 2016;42(4):429-43.

172. Tamburus NY, Kunz VC, Salviati MR, Castello Simoes V, Catai AM, Da Silva E. Interval training based on ventilatory anaerobic threshold improves aerobic functional capacity and metabolic profile: a randomized controlled trial in coronary artery disease patients. Eur J Phys Rehabil Med. 2016;52(1):1-11.

173. Tang A, Eng JJ, Krassioukov AV, Madden KM, Mohammadi A, Tsang MY, et al. Exercise-induced changes in cardiovascular function after stroke: a randomized controlled trial. Int J Stroke. 2014;9(7):883-9.

174. Tarp J, Jespersen E, Moller NC, Klakk H, Wessner B, Wedderkopp N, et al. Long-term follow-up on biological risk factors, adiposity, and cardiorespiratory fitness development in a physical education intervention: a natural experiment (CHAMPS-study DK). BMC Public Health. 2018;18(1):605.

175. Telford RD, Cunningham RB, Waring P, Telford RM, Olive LS, Abhayaratna WP. Physical education and blood lipid concentrations in children: the LOOK randomized cluster trial. PLoS One. 2013;8(10):e76124.

176. Terada T, Friesen A, Chahal BS, Bell GJ, McCargar LJ, Boule NG. Feasibility and preliminary efficacy of high intensity interval training in type 2 diabetes. Diabetes Res Clin Pract. 2013;99(2):120-9.

177. Tillin T, Tuson C, Sowa B, Chattopadhyay K, Sattar N, Welsh P, et al. Yoga and Cardiovascular Health Trial (YACHT): a UK-based randomised mechanistic study of a yoga intervention plus usual care versus usual care alone following an acute coronary event. BMJ Open. 2019;9(11):e030119.

178. Toledo FG, Menshikova EV, Azuma K, Radikova Z, Kelley CA, Ritov VB, et al. Mitochondrial capacity in skeletal muscle is not stimulated by weight loss despite increases in insulin action and decreases in intramyocellular lipid content. Diabetes. 2008;57(4):987-94.

179. Toscano CVA, Carvalho HM, Ferreira JP. Exercise Effects for Children With Autism Spectrum Disorder: Metabolic Health, Autistic Traits, and Quality of Life. Percept Mot Skills. 2018;125(1):126-46.

180. Uth J, Fristrup B, Haahr RD, Brasso K, Helge JW, Rorth M, et al. Football training over 5 years is associated with preserved femoral bone mineral density in men with prostate cancer. Scand J Med Sci Sports. 2018;28 Suppl 1:61-73.

181. Uth J, Fristrup B, Sorensen V, Helge EW, Christensen MK, Kjaergaard JB, et al. Exercise intensity and cardiovascular health outcomes after 12 months of football fitness training in women treated for stage I-III breast cancer: Results from the football fitness After Breast Cancer (ABC) randomized controlled trial. Prog Cardiovasc Dis. 2020;63(6):792-9.

182. van Aggel-Leijssen DP, Saris WH, Homan M, van Baak MA. The effect of exercise training on beta-adrenergic stimulation of fat metabolism in obese men. Int J Obes Relat Metab Disord. 2001;25(1):16-23.

183. van Aggel-Leijssen DP, Saris WH, Hul GB, van Baak MA. Short-term effects of weight loss with or without low-intensity exercise training on fat metabolism in obese men. Am J Clin Nutr. 2001;73(3):523-31.

184. Van Ryckeghem L, Keytsman C, De Brandt J, Verboven K, Verbaanderd E, Marinus N, et al. Impact of continuous vs. interval training on oxygen extraction and cardiac function during exercise in type 2 diabetes mellitus. Eur J Appl Physiol. 2022;122(4):875-87.

185. van Schijndel-Speet M, Evenhuis HM, van Wijck R, van Montfort KC, Echteld MA. A structured physical activity and fitness programme for older adults with intellectual disabilities: results of a cluster-randomised clinical trial. J Intellect Disabil Res. 2017;61(1):16-29.

186. Vasconcellos F, Seabra A, Cunha F, Montenegro R, Penha J, Bouskela E, et al. Health markers in obese adolescents improved by a 12-week recreational soccer program: a randomised controlled trial. J Sports Sci. 2016;34(6):564-75.

187. Vella CA, Taylor K, Drummer D. High-intensity interval and moderate-intensity continuous training elicit similar enjoyment and adherence levels in overweight and obese adults. Eur J Sport Sci. 2017;17(9):1203-11.

188. Vissers D, Verrijken A, Mertens I, Van Gils C, Van de Sompel A, Truijen S, et al. Effect of long-term whole body vibration training on visceral adipose tissue: a preliminary report. Obes Facts. 2010;3(2):93-100.

189. Volpe SL, Kobusingye H, Bailur S, Stanek E. Effect of diet and exercise on body composition, energy intake and leptin levels in overweight women and men. J Am Coll Nutr. 2008;27(2):195-208.

190. Wallman K, Plant LA, Rakimov B, Maiorana AJ. The effects of two modes of exercise on aerobic fitness and fat mass in an overweight population. Res Sports Med. 2009;17(3):156-70.

191. Warner JG, Jr., Ullrich IH, Albrink MJ, Yeater RA. Combined effects of aerobic exercise and omega-3 fatty acids in hyperlipidemic persons. Med Sci Sports Exerc. 1989;21(5):498-505.

192. Wasenius NS, Isomaa BA, Ostman B, Soderstrom J, Forsen B, Lahti K, et al. Low-cost exercise interventions improve long-term cardiometabolic health independently of a family history of type 2 diabetes: a randomized parallel group trial. BMJ Open Diabetes Res Care. 2020;8(2).

193. Weiss EP, Albert SG, Reeds DN, Kress KS, McDaniel JL, Klein S, et al. Effects of matched weight loss from calorie restriction, exercise, or both on cardiovascular disease risk factors: a randomized intervention trial. Am J Clin Nutr. 2016;104(3):576-86.

194. Wilund KR, Colvin PL, Phares D, Goldberg AP, Hagberg JM. The effect of endurance exercise training on plasma lipoprotein AI and lipoprotein AI: AII concentrations in sedentary adults. Metabolism-Clinical and Experimental. 2002;51(8):1053-60.

195. Wilund KR, Ferrell RE, Phares DA, Goldberg AP, Hagberg JM. Changes in high-density lipoprotein-cholesterol subfractions with exercise training may be dependent on cholesteryl ester transfer protein (CETP) genotype. Metabolism. 2002;51(6):774-8.

196. Winn NC, Liu Y, Rector RS, Parks EJ, Ibdah JA, Kanaley JA. Energy-matched moderate and high intensity exercise training improves nonalcoholic fatty liver disease risk independent of changes in body mass or abdominal adiposity - A randomized trial. Metabolism. 2018;78:128-40.

197. Yates T, Davies MJ, Edwardson C, Bodicoat DH, Biddle SJ, Khunti K. Adverse responses and physical activity: secondary analysis of the PREPARE trial. Med Sci Sports Exerc. 2014;46(8):1617-23.

198. Yoshizawa M, Maeda S, Miyaki A, Misono M, Saito Y, Tanabe K, et al. Effect of 12 weeks of moderate-intensity resistance training on arterial stiffness: a randomised controlled trial in women aged 32-59 years. Br J Sports Med. 2009;43(8):615-8.

199. Zaman GS, Abohashrh M, Ahmad I, Dera AA, Alshahrani MS, Ahmad I, et al. The Impact of Body Resistance Training Exercise on Biomedical Profile at High Altitude: A Randomized Controlled Trial. Biomed Res Int. 2021;2021:6684167.

200. Zanetti HR, Mendes EL, Goncalves A, Lopes LT, Roever L, Silva-Vergara ML, et al. Effects of exercise training and statin on hemodynamic, biochemical, inflammatory and immune profile of people living with HIV: a randomized, double-blind, placebo-controlled trial. J Sports Med Phys Fitness. 2020;60(9):1275-82.

201. Zheng G, Xia R, Zhou W, Tao J, Chen L. Aerobic exercise ameliorates cognitive function in older adults with mild cognitive impairment: a systematic review and meta-analysis of randomised controlled trials. Br J Sports Med. 2016;50(23):1443-50.

202. Zou J, Wang Z, Qu Q, Wang L. Resistance training improves hyperglycemia and dyslipidemia, highly prevalent among nonelderly, nondiabetic, chronically disabled stroke patients. Arch Phys Med Rehabil. 2015;96(7):1291-6.

203. Zourladani A, Zafrakas M, Chatzigiannis B, Papasozomenou P, Vavilis D, Matziari C. The effect of physical exercise on postpartum fitness, hormone and lipid levels: a randomized controlled trial in primiparous, lactating women. Arch Gynecol Obstet. 2015;291(3):525-30.

204. Abdelbasset WK, Tantawy SA, Kamel DM, Alqahtani BA, Soliman GS. A randomized controlled trial on the effectiveness of 8-week high-intensity interval exercise on intrahepatic triglycerides, visceral lipids, and health-related quality of life in diabetic obese patients with nonalcoholic fatty liver disease. Medicine. 2019;98(12).

205. Abdelbasset WK, Tantawy SA, Kamel DM, Alqahtani BA, Elnegamy TE, Soliman GS, et al. Effects of high-intensity interval and moderate-intensity continuous aerobic exercise on diabetic obese patients with nonalcoholic fatty liver disease: a comparative randomized controlled trial. Medicine. 2020;99(10).

206. Afzalpour M, Gharakhanlou R, Gaeini A, Mohebbi H, Hedayati M, Khazaei M. The effects of aerobic exercises on the serum oxidized LDL and total antioxidant capacity in non-active men. CVD prevention and control. 2008;3(2):77-82.

207. Agner VFC, Garcia MC, Taffarel AA, Mourão CB, da Silva IP, da Silva SP, et al. Effects of concurrent training on muscle strength in older adults with metabolic syndrome: A randomized controlled clinical trial. Archives of gerontology and geriatrics. 2018;75:158-64.

208. Akinci B, Yeldan I, Satman I, Dirican A, Ozdincler AR. The effects of Internet-based exercise compared with supervised group exercise in people with type 2 diabetes: a randomized controlled study. Clinical Rehabilitation. 2018;32(6):799-810.

209. Aldred H, Hardman A, Taylor S. Influence of 12 weeks of training by brisk walking on postprandial lipemia and insulinemia in sedentary middle-aged women. Metabolism. 1995;44(3):390-7.

210. Álvarez C, Ramírez‐Campillo R, Lucia A, Ramírez‐Vélez R, Izquierdo M. Concurrent exercise training on hyperglycemia and comorbidities associated: Non‐responders using clinical cutoff points. Scandinavian Journal of Medicine & Science in Sports. 2019;29(7):952-67.

211. Arca EA, Martinelli B, Martin LC, Waisberg CB, Franco RJdS. Aquatic exercise is as effective as dry land training to blood pressure reduction in postmenopausal hypertensive women. Physiotherapy Research International. 2014;19(2):93-8.

212. Babu AF, Csader S, Männistö V, Tauriainen M-M, Pentikäinen H, Savonen K, et al. Effects of exercise on NAFLD using non-targeted metabolomics in adipose tissue, plasma, urine, and stool. Scientific Reports. 2022;12(1):6485.

213. Baker TT, Allen D, Lei K, Willcox KK. Alterations in lipid and protein profiles of plasma lipoproteins in middle-aged men consequent to an aerobic exercise program. Metabolism. 1986;35(11):1037-43.

214. Biddle M, Vincent G, McCambridge A, Britton G, Dewes O, Elley CR, et al. Randomised controlled trial of informal team sports for cardirespiratory fitness and health benefit in Pacific Adults. Journal of Primary Health Care. 2011;3(4):269-77.

215. Binder EF, Birge SJ, Kohrt WM. Effects of endurance exercise and hormone replacement therapy on serum lipids in older women. Journal of the American Geriatrics Society. 1996;44(3):231-6.

216. Biteli P, Barbalho SM, Detregiachi CRP, dos Santos Haber JF, Chagas EFB. Dyslipidemia influences the effect of physical exercise on inflammatory markers on obese women in post-menopause: A randomized clinical trial. Experimental Gerontology. 2021;150:111355.

217. Blumenthal JA, Matthews K, Fredrikson M, Rifai N, Schniebolk S, German D, et al. Effects of exercise training on cardiovascular function and plasma lipid, lipoprotein, and apolipoprotein concentrations in premenopausal and postmenopausal women. Arterioscler Thromb. 1991;11(4):912-7.

218. Boardley D, Fahlman M, Topp R, Morgan AL, McNevin N. The impact of exercise training on blood lipids in older adults. Am J Geriatr Cardiol. 2007;16(1):30-5.

219. Bobeuf F, Labonte M, Dionne I, Khalil A. Combined effect of antioxidant supplementation and resistance training on oxidative stress markers, muscle and body composition in an elderly population. The journal of nutrition, health & aging. 2011;15:883-9.

220. Bock BC, Dunsiger SI, Ciccolo JT, Serber ER, Wu W-C, Tilkemeier P, et al. Exercise videogames, physical activity, and health: wii heart fitness: a randomized clinical trial. American journal of preventive medicine. 2019;56(4):501-11.

221. Bonfante ILP, Chacon-Mikahil MPT, Brunelli DT, Gaspari AF, Duft RG, Lopes WA, et al. Combined training, FNDC5/irisin levels and metabolic markers in obese men: A randomised controlled trial. European journal of sport science. 2017;17(5):629-37.

222. Bonfante ILP, Monfort-Pires M, Duft RG, da Silva Mateus KC, de Lima Júnior JC, dos Santos Trombeta JC, et al. Combined training increases thermogenic fat activity in patients with overweight and type 2 diabetes. Int J Obes. 2022;46(6):1145-54.

223. Boreham CA, Wallace WF, Nevill A. Training effects of accumulated daily stair-climbing exercise in previously sedentary young women. Preventive Medicine. 2000;30(4):277-81.

224. Boyden TW, Pamenter RW, Going SB, Lohman TG, Hall MC, Houtkooper LB, et al. Resistance exercise training is associated with decreases in serum low-density lipoprotein cholesterol levels in premenopausal women. Archives of internal medicine. 1993;153(1):97-100.

225. Busby J, Notelovitz M, Putney K, Grow T. Exercise, high-density lipoprotein-cholesterol, and cardiorespiratory function in climacteric women. Southern medical journal. 1985;78(7):769-73.

226. Cho J-K, Lee S-H, Lee J-Y, Kang H-S. Randomized controlled trial of training intensity in adiposity. International journal of sports medicine. 2011:468-75.

227. Church TS, Earnest CP, Skinner JS, Blair SN. Effects of different doses of physical activity on cardiorespiratory fitness among sedentary, overweight or obese postmenopausal women with elevated blood pressure: a randomized controlled trial. JAMA. 2007;297(19):2081-91.

228. Colado JC, Triplett NT, Tella V, Saucedo P, Abellan J. Effects of aquatic resistance training on health and fitness in postmenopausal women. Eur J Appl Physiol. 2009;106(1):113-22.

229. Connolly LJ, Scott S, Morencos CM, Fulford J, Jones AM, Knapp K, et al. Impact of a novel home-based exercise intervention on health indicators in inactive premenopausal women: a 12-week randomised controlled trial. European Journal of Applied Physiology. 2020;120:771-82.

230. Costa RR, Pilla C, Buttelli ACK, Barreto MF, Vieiro PA, Alberton CL, et al. Water-based aerobic training successfully improves lipid profile of dyslipidemic women: a randomized controlled trial. Research quarterly for exercise and sport. 2018;89(2):173-82.

231. Cunningham D, Rechnitzer P, Howard J, Donner A. Exercise training of men at retirement: a clinical trial. Journal of Gerontology. 1987;42(1):17-23.

232. Dalleck LC, Allen BA, Hanson BA, Borresen EC, Erickson ME, De Lap SL. Dose-response relationship between moderate-intensity exercise duration and coronary heart disease risk factors in postmenopausal women. J Womens Health (Larchmt). 2009;18(1):105-13.

233. Dardashti pour E, Yaghobian F, Dehghan F, Azarbayjani MA. Forecast of ameliorating effect of dietary flavonol consumption in white tea with or without aerobic training on type 2 diabetes (T2D) in females. Clinical nutrition ESPEN. 2021;45:134-40.

234. Duncan K, Pozehl B. Effects of an exercise adherence intervention on outcomes in patients with heart failure. Rehabil Nurs. 2003;28(4):117-22.

235. Eguchi Y, Ohta M, Inoue T, Honda T, Morita Y, Konno Y, et al. Effects of transitory stimulation interval exercise on physical function: a randomized controlled pilot study among Japanese subjects. Journal of UOEH. 2012;34(4):297-308.

236. Elliott K, Sale C, Cable N. Effects of resistance training and detraining on muscle strength and blood lipid profiles in postmenopausal women. British journal of sports medicine. 2002;36(5):340-4.

237. Fahlman MM, Boardley D, Lambert CP, Flynn MG. Effects of endurance training and resistance training on plasma lipoprotein profiles in elderly women. The Journals of Gerontology Series A: Biological Sciences and Medical Sciences. 2002;57(2):B54-B60.

238. Fang Y-Y, Huang C-Y, Hsu M-C. Effectiveness of a physical activity program on weight, physical fitness, occupational stress, job satisfaction and quality of life of overweight employees in high-tech industries: a randomized controlled study. International Journal of Occupational Safety and Ergonomics. 2019;25(4):621-9.

239. Farinha C, Santos H, Serrano J, Oliveiros B, Silva FM, Cascante-Rusenhack M, et al. The Impact of Aquatic Exercise Programs on the Intima-Media thickness of the Carotid Arteries, Hemodynamic Parameters, Lipid Profile and Chemokines of Community-Dwelling Older Persons: A Randomized Controlled Trial. International journal of environmental research and public health. 2022;19(6):3377.

240. Fenkci S, Sarsan A, Rota S, Ardic F. Effects of resistance or aerobic exercises on metabolic parameters in obese women who are not on a diet. Advances in therapy. 2006;23:404-13.

241. Finucane F, Sharp S, Purslow L, Horton K, Horton J, Savage D, et al. The effects of aerobic exercise on metabolic risk, insulin sensitivity and intrahepatic lipid in healthy older people from the Hertfordshire Cohort Study: a randomised controlled trial. Diabetologia. 2010;53:624-31.

242. Foulds HJ, Bredin SS, Charlesworth SA, Ivey AC, Warburton DE. Exercise volume and intensity: a dose–response relationship with health benefits. European journal of applied physiology. 2014;114:1563-71.

243. Frank LL, Sorensen BE, Yasui Y, Tworoger SS, Schwartz RS, Ulrich CM, et al. Effects of exercise on metabolic risk variables in overweight postmenopausal women: a randomized clinical trial. Obesity research. 2005;13(3):615-25.

244. Gram B, Christensen R, Christiansen C, Gram J. Effects of nordic walking and exercise in type 2 diabetes mellitus: a randomized controlled trial. Clinical Journal of Sport Medicine. 2010;20(5):355-61.

245. Gram AS, Petersen MB, Quist JS, Rosenkilde M, Stallknecht B, Bladbjerg EM. Effects of 6 Months of Active Commuting and Leisure-Time Exercise on Fibrin Turnover in Sedentary Individuals with Overweight and Obesity: A Randomised Controlled Trial. J Obes. 2018;2018:7140754.

246. Grandjean PW, Oden GL, Crouse SF, Brown JA, Green JS. Lipid and lipoprotein changes in women following 6 months of exercise training in a worksite fitness program. The Journal Of Sports Medicine And Physical Fitness. 1996;36(1):54-9.

247. Ha M-S, Kim J-H, Kim Y-S, Kim D-Y. Effects of aquarobic exercise and burdock intake on serum blood lipids and vascular elasticity in Korean elderly women. Experimental gerontology. 2018;101:63-8.

248. Hagan RD, Upton SJ, Wong L, Whittam J. The effects of aerobic conditioning and/or caloric restriction in overweight men and women. Med Sci Sports Exerc. 1986;18(1):87-94.

249. Hagerman FC, Walsh SJ, Staron RS, Hikida RS, Gilders RM, Murray TF, et al. Effects of high-intensity resistance training on untrained older men. I. Strength, cardiovascular, and metabolic responses. The journals of gerontology series A: Biological Sciences and medical sciences. 2000;55(7):B336-B46.

250. Hallsworth K, Thoma C, Hollingsworth KG, Cassidy S, Anstee QM, Day CP, et al. Modified high-intensity interval training reduces liver fat and improves cardiac function in non-alcoholic fatty liver disease: a randomized controlled trial. Clinical science. 2015;129(12):1097-105.

251. Hellenius ML, de Faire U, Berglund B, Hamsten A, Krakau I. Diet and exercise are equally effective in reducing risk for cardiovascular disease. Results of a randomized controlled study in men with slightly to moderately raised cardiovascular risk factors. Atherosclerosis. 1993;103(1):81-91.

252. Hersey III WC, Graves JE, Pollock ML, Gingerich R, Shireman RB, Heath GW, et al. Endurance exercise training improves body composition and plasma insulin responses in 70-to 79-year-old men and women. Metabolism. 1994;43(7):847-54.

253. Herzig K, Ahola R, Leppäluoto J, Jokelainen J, Jämsä T, Keinänen-Kiukaanniemi S. Light physical activity determined by a motion sensor decreases insulin resistance, improves lipid homeostasis and reduces visceral fat in high-risk subjects: PreDiabEx study RCT. Int J Obes. 2014;38(8):1089-96.

254. Hinkleman L, Nieman D. The effects of a walking program on body composition and serum lipids and lipoproteins in overweight women. The journal of sports medicine and physical fitness. 1993;33(1):49-58.

255. Ho SS, Dhaliwal SS, Hills AP, Pal S. The effect of 12 weeks of aerobic, resistance or combination exercise training on cardiovascular risk factors in the overweight and obese in a randomized trial. BMC public health. 2012;12(1):1-10.

256. Hornstrup T, Løwenstein FT, Larsen MA, Helge EW, Póvoas S, Helge J, et al. Cardiovascular, muscular, and skeletal adaptations to recreational team handball training: a randomized controlled trial with young adult untrained men. European journal of applied physiology. 2019;119:561-73.

257. Houghton D, Thoma C, Hallsworth K, Cassidy S, Hardy T, Burt AD, et al. Exercise reduces liver lipids and visceral adiposity in patients with nonalcoholic steatohepatitis in a randomized controlled trial. Clinical Gastroenterology and Hepatology. 2017;15(1):96-102. e3.

258. Hsieh P-L, Tseng C-H, Tseng YJ, Yang W-S. Resistance training improves muscle function and cardiometabolic risks but not quality of life in older people with type 2 diabetes mellitus: a randomized controlled trial. Journal of Geriatric Physical Therapy. 2018;41(2):65-76.

259. Hurley B, Hagberg J, Goldberg A, Seals D, Ehsani A, Brennan R, et al. Resistive training can reduce coronary risk factors without altering VO2max or percent body fat. Medicine and science in sports and exercise. 1988;20(2):150-4.

260. Huttunen JK, Länsimies E, Voutilainen E, Ehnholm C, Hietanen E, Penttilä I, et al. Effect of moderate physical exercise on serum lipoproteins. A controlled clinical trial with special reference to serum high-density lipoproteins. Circulation. 1979;60(6):1220-9.

261. Isler AK, Kosar S, Korkusuz F. Effects of step aerobics and aerobic dancing on serum lipids and lipoproteins. Journal of sports medicine and physical fitness. 2001;41(3):380-5.

262. Jennings F, Oliveira HA, de Souza MC, da Graça Cruz V, Natour J. Effects of aerobic training in patients with ankylosing spondylitis. The Journal of rheumatology. 2015;42(12):2347-53.

263. Juneau M, Rogers F, De Santos V, Yee M, Evans A, Bohn A, et al. Effectiveness of self-monitored, home-based, moderate-intensity exercise training in middle-aged men and women. The American journal of cardiology. 1987;60(1):66-70.

264. Kantyka J, Herman D, Roczniok R, Kuba L. Effects of aqua aerobics on body composition, body mass, lipid profile, and blood count in middle-aged sedentary women. Human Movement. 2015;16(1):9-14.

265. Kiens B, JORGENSEN I, Lewis S, JENSEN G, LITHELL H, VESSBY B, et al. Increased plasma HDL‐cholesterol and apo A‐1 in sedentary middle‐aged men after physical conditioning. European journal of clinical investigation. 1980;10(3):203-9.

266. Woo-Cheol K, Suk-Lip C, Sung-Woon K, Hae-Ryoung P. The effects of aquarobics on blood pressure, heart rate, and lipid profile in older women with hypertension. Indian J Sci Technol. 2016;9(46):1-7.

267. Knight E, Stuckey MI, Petrella RJ. Prescribing physical activity through primary care: does activity intensity matter? The Physician and Sportsmedicine. 2014;42(3):78-9.

268. Kraus WE, Houmard JA, Duscha BD, Knetzger KJ, Wharton MB, McCartney JS, et al. Effects of the amount and intensity of exercise on plasma lipoproteins. The New England journal of medicine. 2002;347(19):1483-92.

269. Krustrup P, Nielsen JJ, Krustrup BR, Christensen JF, Pedersen H, Randers MB, et al. Recreational soccer is an effective health-promoting activity for untrained men. British journal of sports medicine. 2009;43(11):825-31.

270. Kukkonen‐Harjula K, Laukkanen R, Vuori I, Oja P, Pasanen M, Nenonen A, et al. Effects of walking training on health‐related fitness in healthy middle‐aged adults—a randomized controlled study. Scandinavian journal of medicine & science in sports. 1998;8(4):236-42.

271. Laaksonen D, Atalay M, Niskanen L, Mustonen J, Lakka T, Uusitupa MI. Aerobic exercise and the lipid profile in type 1 diabetic men: a randomized controlled trial. Medicine & Science in Sports & Exercise. 2000;32(9):1541-8.

272. Lamina S, Okoye CG, Hanif SM. Randomised controlled trial: effects of aerobic exercise training programme on indices of adiposity and metabolic markers in hypertension. JPMA The Journal of the Pakistan Medical Association. 2013;63(6):680-7.

273. LeMura LM, von Duvillard SP, Andreacci J, Klebez JM, Chelland SA, Russo J. Lipid and lipoprotein profiles, cardiovascular fitness, body composition, and diet during and after resistance, aerobic and combination training in young women. European journal of applied physiology. 2000;82:451-8.

274. Casal D, Leon A, Moy J, Shaw G, McNally C, Hughes J. Effects of 2000 KCAL per week of treadmill walking and stairclimbing on coronary risk factors. Medicine & Science in Sports & Exercise. 1983;15(2):149.

275. Liao H-c, Zhong S-g, Li P, Chen W-b, Cheng C, Wang Y-g, et al. Effects and mechanism of moderate aerobic exercise on impaired fasting glucose improvement. Lipids in health and disease. 2015;14:1-6.

276. Libardi CA, De Souza GV, Cavaglieri CR, Madruga VA, Chacon-Mikahil M. Effect of resistance, endurance, and concurrent training on TNF-a, IL-6, and CRP. Med Sci Sports Exerc. 2012;44(5):50-6.

277. Magalhães JP, Melo X, Correia IR, Ribeiro RT, Raposo J, Dores H, et al. Effects of combined training with different intensities on vascular health in patients with type 2 diabetes: a 1-year randomized controlled trial. Cardiovascular diabetology. 2019;18(1):1-13.

278. Manning J, Dooly-Manning C, White K, Kampa I, Silas S, Kesselhaut M, et al. Effects of a resistive training program on lipoprotein--lipid levels in obese women. Medicine and science in sports and exercise. 1991;23(11):1222-6.

279. Mashnafi S, Plat J, Mensink R, Joris P, Kleinloog J, Baumgartner S. Effects of an 8-week aerobic exercise program on plasma markers for cholesterol absorption and synthesis in older overweight and obese men. Lipids in Health and Disease. 2021;20(1):1-8.

280. Mendham AE, Duffield R, Marino F, Coutts AJ. A 12-week sports-based exercise programme for inactive Indigenous Australian men improved clinical risk factors associated with type 2 diabetes mellitus. Journal of Science and Medicine in Sport. 2015;18(4):438-43.

281. Miyaki A, Maeda S, Choi Y, Akazawa N, Tanabe Y, Ajisaka R. Habitual aerobic exercise increases plasma pentraxin 3 levels in middle-aged and elderly women. Applied physiology, nutrition, and metabolism. 2012;37(5):907-11.

282. Mohr M, Lindenskov A, Holm P, Nielsen H, Mortensen J, Weihe P, et al. Football training improves cardiovascular health profile in sedentary, premenopausal hypertensive women. Scandinavian journal of medicine & science in sports. 2014;24:36-42.

283. Morey MC, Pieper CF, Edelman DE, Yancy Jr WS, Green JB, Lum H, et al. Enhanced fitness: a randomized controlled trial of the effects of home‐based physical activity counseling on glycemic control in older adults with prediabetes mellitus. Journal of the American Geriatrics Society. 2012;60(9):1655-62.

284. Morgan AL, Tobar DA, Snyder L. Walking toward a new me: the impact of prescribed walking 10,000 steps/day on physical and psychological well-being. Journal of physical activity and health. 2010;7(3):299-307.

285. Motoyama M, Sunami Y, Kinoshita F, Irie T, Sasaki J, Arakawa K, et al. The effects of long-term low intensity aerobic training and detraining on serum lipid and lipoprotein concentrations in elderly men and women. European journal of applied physiology and occupational physiology. 1995;70:126-31.

286. Murphy MH, Murtagh EM, Boreham CA, Hare LG, Nevill AM. The effect of a worksite based walking programme on cardiovascular risk in previously sedentary civil servants [NCT00284479]. BMC public health. 2006;6:1-8.

287. Murtagh EM, Boreham CA, Nevill A, Hare LG, Murphy MH. The effects of 60 minutes of brisk walking per week, accumulated in two different patterns, on cardiovascular risk. Preventive medicine. 2005;41(1):92-7.

288. Musa DI, Adeniran SA, Dikko A, Sayers SP. The effect of a high-intensity interval training program on high-density lipoprotein cholesterol in young men. The journal of strength & conditioning research. 2009;23(2):587-92.

289. Neves LM, Fortaleza AC, Rossi FE, Diniz TA, Codogno JS, Gobbo LA, et al. Functional training reduces body fat and improves functional fitness and cholesterol levels in postmenopausal women: a randomized clinical trial. J Sports Med Phys Fitness. 2017;57(4):448-56.

290. Niederseer D, Ledl‐Kurkowski E, Kvita K, Patsch W, Dela F, Mueller E, et al. Salzburg Skiing for the Elderly Study: changes in cardiovascular risk factors through skiing in the elderly. Scandinavian journal of medicine & science in sports. 2011;21:47-55.

291. Nielsen T-T, Møller TK, Olesen ND, Zebis MK, Ritz C, Nordsborg N, et al. Improved metabolic fitness, but no cardiovascular health effects, of a low-frequency short-term combined exercise programme in 50–70-year-olds with low fitness: A randomized controlled trial. European Journal of Sport Science. 2022;22(3):460-73.

292. Nieman DC, Warren BJ, O'Donnell KA, Dotson RG, Butterworth DE, Henson DA. Physical activity and serum lipids and lipoproteins in elderly women. Journal of the American Geriatrics Society. 1993;41(12):1339-44.

293. Nieman DC, Brock DW, Butterworth D, Utter AC, Nieman CC. Reducing diet and/or exercise training decreases the lipid and lipoprotein risk factors of moderately obese women. Journal of the American College of Nutrition. 2002;21(4):344-50.

294. Nualnim N, Parkhurst K, Dhindsa M, Tarumi T, Vavrek J, Tanaka H. Effects of swimming training on blood pressure and vascular function in adults> 50 years of age. The American journal of cardiology. 2012;109(7):1005-10.

295. Nuttamonwarakul A, Amatyakul S, Suksom D. Twelve weeks of aqua-aerobic exercise improve health-related physical fitness and glycemic control in elderly patients with type 2 diabetes. Journal of Exercise Physiology. 2012;15(2):64-71.

296. Nybo L, Sundstrup E, Jakobsen MD, Mohr M, Hornstrup T, Simonsen L, et al. High-intensity training versus traditional exercise interventions for promoting health. Medicine & Science in Sports & Exercise. 2010;42(10):1951-8.

297. O'Donovan G, Owen A, Bird SR, Kearney EM, Nevill AM, Jones DW, et al. Changes in cardiorespiratory fitness and coronary heart disease risk factors following 24 wk of moderate-or high-intensity exercise of equal energy cost. Journal of applied physiology. 2005;98(5):1619-25.

298. Ohta M, Eguchi Y, Inoue T, Honda T, Morita Y, Konno Y, et al. Effects of bench step exercise intervention on work ability in terms of cardiovascular risk factors and oxidative stress: a randomized controlled study. International Journal of Occupational Safety and Ergonomics. 2015;21(2):141-9.

299. Park S-K, Park J-H, Kwon Y-C, Kim H-S, Yoon M-S, Park H-T. The effect of combined aerobic and resistance exercise training on abdominal fat in obese middle-aged women. Journal of physiological anthropology and applied human science. 2003;22(3):129-35.

300. Park S-Y, Lee I-H. Effects on training and detraining on physical function, control of diabetes and anthropometrics in type 2 diabetes; a randomized controlled trial. Physiotherapy theory and practice. 2015;31(2):83-8.

301. Patterson S, Pattison J, Legg H, Gibson A-M, Brown N. The impact of badminton on health markers in untrained females. Journal of sports sciences. 2017;35(11):1098-106.

302. Pereira R, Krustrup P, Castagna C, Coelho E, Santos R, Martins S, et al. Effects of a 16-week recreational team handball intervention on aerobic performance and cardiometabolic fitness markers in postmenopausal women: A randomized controlled trial. Progress in Cardiovascular Diseases. 2020;63(6):800-6.

303. Prabhakaran B, Dowling EA, Branch JD, Swain DP, Leutholtz BC. Effect of 14 weeks of resistance training on lipid profile and body fat percentage in premenopausal women. British journal of sports medicine. 1999;33(3):190-5.

304. Rahimi A, Shabestari MM, Faryadian K, Safaeinejad V, Moazen JS, Fallah Z. The effect of selecting aerobics exercise program (walking in water and in land) on HDL-C, LDL-C, TC and TG in non-athlete menopausal women. European Journal of Experimental Biology. 2013;3(2):463-8.

305. Ranasinghe C, Devage S, Constantine GR, Katulanda P, Hills AP, King NA. Glycemic and cardiometabolic effects of exercise in South Asian Sri Lankans with type 2 diabetes mellitus: A randomized controlled trial Sri Lanka diabetes aerobic and resistance training study (SL-DARTS). Diabetes & Metabolic Syndrome: Clinical Research & Reviews. 2021;15(1):77-85.

306. Raz I, Rosenblit H, Kark JD. Effect of moderate exercise on serum lipids in young men with low high density lipoprotein cholesterol. Arteriosclerosis: An Official Journal of the American Heart Association, Inc. 1988;8(3):245-51.

307. Ready AE, Naimark B, Ducas J, Sawatzky JV, Boreskie SL, Drinkwater DT, et al. Influence of walking volume on health benefits in women post-menopause. Med Sci Sports Exerc. 1996;28(9):1097-105.

308. Rowland SA, Berg KE, Kupzyk KA, Pullen CH, Cohen MZ, Schulz PS, et al. Feasibility and effect of a peer modeling workplace physical activity intervention for women. Workplace health & safety. 2018;66(9):428-36.

309. Santiago MC, Leon AS, Serfass RC. Failure of 40 weeks of brisk walking to alter blood lipids in normolipemic women. Canadian journal of applied physiology. 1995;20(4):417-28.

310. Sbardelotto ML, Costa RR, Malysz KA, Pedroso GS, Pereira BC, Sorato HR, et al. Improvement in muscular strength and aerobic capacities in elderly people occurs independently of physical training type or exercise model. Clinics. 2019;74.

311. Schroeder EC, Franke WD, Sharp RL, Lee D-c. Comparative effectiveness of aerobic, resistance, and combined training on cardiovascular disease risk factors: A randomized controlled trial. PloS one. 2019;14(1):e0210292.

312. Schuit A, Schouten E, Miles T, Evans W, Saris W, Kok F. The effect of six months training on weight, body fatness and serum lipids in apparently healthy elderly Dutch men and women. Int J Obes. 1998;22(9):847-53.

313. Shaw I, Shaw S, KrasilshchIkov O. Comparison of aerobic and combined aerobic and resistance training on low-density lipoprotein cholesterol concentrations in men: cardiovascular topic. Cardiovascular journal of Africa. 2009;20(5):290-5.

314. Sian TS, Inns TB, Gates A, Doleman B, Bass JJ, Atherton PJ, et al. Equipment-free, unsupervised high intensity interval training elicits significant improvements in the physiological resilience of older adults. BMC geriatrics. 2022;22(1):1-11.

315. Sillanpaa E, Laaksonen DE, Hakkinen A, Karavirta L, Jensen B, Kraemer WJ, et al. Body composition, fitness, and metabolic health during strength and endurance training and their combination in middle-aged and older women. Eur J Appl Physiol. 2009;106(2):285-96.

316. Smith-Ryan AE, Melvin MN, Wingfield HL. High-intensity interval training: Modulating interval duration in overweight/obese men. The Physician and sportsmedicine. 2015;43(2):107-13.

317. Smutok M, Reece C, Kokkinos P, Farmer C, Dawson P, Shulman R, et al. Aerobic versus strength training for risk factor intervention in middle-aged men at high risk for coronary heart disease. Metabolism. 1993;42(2):177-84.

318. So W-y, Song M, Park Y-h, Cho B-l, Lim J-y, Kim S-h, et al. Body composition, fitness level, anabolic hormones, and inflammatory cytokines in the elderly: a randomized controlled trial. Aging clinical and experimental research. 2013;25:167-74.

319. Sousa N, Mendes R, Abrantes C, Sampaio J, Oliveira J. A randomized study on lipids response to different exercise programs in overweight older men. International journal of sports medicine. 2014:1106-11.

320. Stefanick ML, Mackey S, Sheehan M, Ellsworth N, Haskell WL, Wood PD. Effects of diet and exercise in men and postmenopausal women with low levels of HDL cholesterol and high levels of LDL cholesterol. N Engl J Med. 1998;339(1):12-20.

321. Stein RA, Michielli DW, Glantz MD, Sardy H, Cohen A, Goldberg N, et al. Effects of different exercise training intensities on lipoprotein cholesterol fractions in healthy middle-aged men. Am Heart J. 1990;119(2 Pt 1):277-83.

322. Stensel D, Hardman A, Brooke-Wavell K, Vallance D, Jones P, Norgan N, et al. Brisk walking and serum lipoprotein variables in formerly sedentary men aged 42-59 years. Clinical Science (London, England: 1979). 1993;85(6):701-8.

323. Stensvold D, Tjonna AE, Skaug EA, Aspenes S, Stolen T, Wisloff U, et al. Strength training versus aerobic interval training to modify risk factors of metabolic syndrome. J Appl Physiol. 2010;108(4):804-10.

324. Stewart KJ, Bacher AC, Turner K, Lim JG, Hees PS, Shapiro EP, et al. Exercise and risk factors associated with metabolic syndrome in older adults. American journal of preventive medicine. 2005;28(1):9-18.

325. Sunami Y, Motoyama M, Kinoshita F, Mizooka Y, Sueta K, Matsunaga A, et al. Effects of low-intensity aerobic training on the high-density lipoprotein cholesterol concentration in healthy elderly subjects. Metabolism. 1999;48(8):984-8.

326. Suter E, Marti B. Little effect of long-term, self-monitored exercise on serum lipid levels in middle-aged women. The Journal of sports medicine and physical fitness. 1992;32(4):400-11.

327. Suter E, Marti B, Gutzwiller F. Jogging or walking—comparison of health effects. Annals of epidemiology. 1994;4(5):375-81.

328. Swift DL, Nevels TR, Solar CA, Brophy PM, Mcgee JE, Brewer SB, et al. The Effect of Aerobic Training and Increasing Nonexercise Physical Activity on Cardiometabolic Risk Factors. Medicine and Science in Sports and Exercise. 2021;53(10):2152-63.

329. Takeshima N, Rogers ME, Watanabe E, Brechue WF, Okada A, Yamada T, et al. Water-based exercise improves health-related aspects of fitness in older women. Medicine & Science in Sports & Exercise. 2002;34(3):544-51.

330. Takeshima N, Rogers ME, Islam MM, Yamauchi T, Watanabe E, Okada A. Effect of concurrent aerobic and resistance circuit exercise training on fitness in older adults. European journal of applied physiology. 2004;93:173-82.

331. Taniguchi H, Tanisawa K, Sun X, Kubo T, Higuchi M. Endurance exercise reduces hepatic fat content and serum fibroblast growth factor 21 levels in elderly men. The Journal of Clinical Endocrinology. 2016;101(1):191-8.

332. Thomas T, Adeniran S, Etheridge G. Effects of different running programs on VO2 max, percent fat, and plasma lipids. Canadian journal of applied sport sciences Journal canadien des sciences appliquees au sport. 1984;9(2):55-62.

333. Thompson D, Markovitch D, Betts JA, Mazzatti D, Turner J, Tyrrell RM. Time course of changes in inflammatory markers during a 6-mo exercise intervention in sedentary middle-aged men: a randomized-controlled trial. Journal of applied physiology. 2010;108(4):769-79.

334. Tjønna AE, Lee SJ, Rognmo Ø, Stølen TO, Bye A, Haram PM, et al. Aerobic interval training versus continuous moderate exercise as a treatment for the metabolic syndrome: a pilot study. Circulation. 2008;118(4):346-54.

335. Tokudome M, Nagasaki M, Shimaoka K, Sato Y. Effects of home‐based combined resistance training and walking on metabolic profiles in elderly Japanese. Geriatrics & gerontology international. 2004;4(3):157-62.

336. Trajković N, Sporiš G, Krističević T, Bogataj Š. Effects of small-sided recreational volleyball on health markers and physical fitness in middle-aged men. International journal of environmental research and public health. 2020;17(9):3021.

337. Veríssimo MT, Aragão A, Sousa A, Barbosa B, Ribeiro H, Costa D, et al. Effect of physical exercise on lipid metabolism in the elderly. Revista portuguesa de cardiologia: orgao oficial da Sociedade Portuguesa de Cardiologia= Portuguese journal of cardiology: an official journal of the Portuguese Society of Cardiology. 2002;21(10):1099-112.

338. Vesterbekkmo EK, Madssen E, Aamot Aksetøy IL, Follestad T, Nilsen HO, Hegbom K, et al. CENIT (impact of cardiac exercise training on lipid content in coronary atheromatous plaques evaluated by near‐infrared spectroscopy): a randomized trial. Journal of the American Heart Association. 2022;11(10):e024705.

339. Vincent KR, Braith RW, Bottiglieri T, Vincent HK, Lowenthal DT. Homocysteine and lipoprotein levels following resistance training in older adults. Preventive cardiology. 2003;6(4):197-203.

340. Vincent HK, Bourguignon C, Vincent KR. Resistance training lowers exercise‐induced oxidative stress and homocysteine levels in overweight and obese older adults. Obesity. 2006;14(11):1921-30.

341. Vicente-Campos D, Mora J, Castro-Pinero J, González-Montesinos J, Conde-Caveda J, Chicharro J. Impact of a physical activity program on cerebral vasoreactivity in sedentary elderly people. Journal of sports medicine and physical fitness. 2012;52(5):537.

342. Vinetti G, Mozzini C, Desenzani P, Boni E, Bulla L, Lorenzetti I, et al. Supervised exercise training reduces oxidative stress and cardiometabolic risk in adults with type 2 diabetes: a randomized controlled trial. Scientific reports. 2015;5(1):9238.

343. Watkins LL, Sherwood A, Feinglos M, Hinderliter A, Babyak M, Gullette E, et al. Effects of exercise and weight loss on cardiac risk factors associated with syndrome X. Archives of internal medicine. 2003;163(16):1889-95.

344. Wen H, Huang T, Li T, Chong P, Ang B. Effects of short-term step aerobics exercise on bone metabolism and functional fitness in postmenopausal women with low bone mass. Osteoporosis International. 2017;28:539-47.

345. Whitehurst M, Menendez E. Endurance training in older women: lipid and lipoprotein responses. The physician and sportsmedicine. 1991;19(6):95-103.

346. Williams PT, Stefanick ML, Vranizan KM, Wood PD. The effects of weight loss by exercise or by dieting on plasma high-density lipoprotein (HDL) levels in men with low, intermediate, and normal-to-high HDL at baseline. Metabolism. 1994;43(7):917-24.

347. Woolf-May K, Kearney E, Jones D, Davison R, Coleman D, Bird S. The effect of two different 18-week walking programmes on aerobic fitness, selected blood lipids and factor XIIa. Journal of Sports Sciences. 1998;16(8):701-10.

348. Wooten JS, Phillips MD, Mitchell JB, Patrizi R, Pleasant RN, Hein RM, et al. Resistance exercise and lipoproteins in postmenopausal women. International journal of sports medicine. 2010:7-13.

349. Wu Y-T, Hwang C-L, Chen C-N, Chuang L-M. Home-based exercise for middle-aged Chinese at diabetic risk: a randomized controlled trial. Preventive Medicine. 2011;52(5):337-43.

350. Zelber-Sagi S, Buch A, Yeshua H, Vaisman N, Webb M, Harari G, et al. Effect of resistance training on non-alcoholic fatty-liver disease a randomized-clinical trial. World journal of gastroenterology: WJG. 2014;20(15):4382.

351. Zhang H-J, He J, Pan L-L, Ma Z-M, Han C-K, Chen C-S, et al. Effects of moderate and vigorous exercise on nonalcoholic fatty liver disease: a randomized clinical trial. JAMA internal medicine. 2016;176(8):1074-82.
